# An Automatic Ontology-Based Approach to Support Logical Representation of Observable and Measurable Data for Healthy Lifestyle Management: Proof-of-Concept Study

**DOI:** 10.2196/24656

**Published:** 2021-04-09

**Authors:** Ayan Chatterjee, Andreas Prinz, Martin Gerdes, Santiago Martinez

**Affiliations:** 1 Department of Information and Communication Technologies Centre for e-Health University of Agder Grimstad Norway; 2 Department of Health and Nursing Science Centre for e-Health University of Agder Grimstad Norway

**Keywords:** activity, nutrition, sensor, questionnaire, SSN, ontology, SNOMED CT, eCoach, personalized, recommendation, automated, CDSS, healthy lifestyle, interoperability, eHealth, goal setting, semantics, simulation, proposition

## Abstract

**Background:**

Lifestyle diseases, because of adverse health behavior, are the foremost cause of death worldwide. An eCoach system may encourage individuals to lead a healthy lifestyle with early health risk prediction, personalized recommendation generation, and goal evaluation. Such an eCoach system needs to collect and transform distributed heterogenous health and wellness data into meaningful information to train an artificially intelligent health risk prediction model. However, it may produce a data compatibility dilemma. Our proposed eHealth ontology can increase interoperability between different heterogeneous networks, provide situation awareness, help in data integration, and discover inferred knowledge. This “proof-of-concept” study will help sensor, questionnaire, and interview data to be more organized for health risk prediction and personalized recommendation generation targeting obesity as a study case.

**Objective:**

The aim of this study is to develop an OWL-based ontology (UiA eHealth Ontology/UiAeHo) model to annotate personal, physiological, behavioral, and contextual data from heterogeneous sources (sensor, questionnaire, and interview), followed by structuring and standardizing of diverse descriptions to generate meaningful, practical, personalized, and contextual lifestyle recommendations based on the defined rules.

**Methods:**

We have developed a simulator to collect dummy personal, physiological, behavioral, and contextual data related to artificial participants involved in health monitoring. We have integrated the concepts of “Semantic Sensor Network Ontology” and “Systematized Nomenclature of Medicine—Clinical Terms” to develop our proposed eHealth ontology. The ontology has been created using Protégé (version 5.x). We have used the Java-based “Jena Framework” (version 3.16) for building a semantic web application that includes resource description framework (RDF) application programming interface (API), OWL API, native tuple store (tuple database), and the SPARQL (Simple Protocol and RDF Query Language) query engine. The logical and structural consistency of the proposed ontology has been evaluated with the “HermiT 1.4.3.x” ontology reasoner available in Protégé 5.x.

**Results:**

The proposed ontology has been implemented for the study case “obesity.” However, it can be extended further to other lifestyle diseases. “UiA eHealth Ontology” has been constructed using logical axioms, declaration axioms, classes, object properties, and data properties. The ontology can be visualized with “Owl Viz,” and the formal representation has been used to infer a participant’s health status using the “HermiT” reasoner. We have also developed a module for ontology verification that behaves like a rule-based decision support system to predict the probability for health risk, based on the evaluation of the results obtained from SPARQL queries. Furthermore, we discussed the potential lifestyle recommendation generation plan against adverse behavioral risks.

**Conclusions:**

This study has led to the creation of a meaningful, context-specific ontology to model massive, unintuitive, raw, unstructured observations for health and wellness data (eg, sensors, interviews, questionnaires) and to annotate them with semantic metadata to create a compact, intelligible abstraction for health risk predictions for individualized recommendation generation.

## Introduction

### Overview

Lifestyle diseases are an economic burden to an individual, household, employer, and government, and lead to financial and productivity risks for poor and rich countries alike [1-3]. The key risk factors behind lifestyle diseases are the excessive use of alcohol, inappropriate food plan, physical inactivity, excessive salt intake, saturated fat consumption, and tobacco use [1-3]. These result in excess weight gain, elevated blood glucose, high blood pressure (BP), elevated total cholesterol in the blood, and social isolation. Obesity is one of the foremost lifestyle diseases that lead to other noncommunicable diseases such as cardiovascular diseases, chronic obstructive pulmonary disease, cancer, diabetes type II, hypertension, and depression [1-3]. eHealth monitoring has become increasingly popular, providing information and communications technology (ICT)–based remote, timely care support to patients and health care providers [1-3]. An eHealth virtual coaching recommendation system can guide people and convey the appropriate recommendations in context with enough time to prevent and improve living with lifestyle diseases. It requires capturing physiological (vital signs such as BP, pulse, lipid profile, glycemic response, BMI), behavioral (sleep, diet, exercise), and contextual data (position, and weather) from secure wearable sensors, manual interactions, feedback, and customized questionnaires over time, to train an artificial intelligence (AI) model for behavior analysis and early prediction of wellness trends and risks [4-6]. However, data collection from heterogenous sources may lead to data interoperability, annotation, and semantization problem.

### Background and Problem Description

Health and wellness data collected from heterogeneous sources (eg, multimodal sensors, interviews, questionnaires) are of different format and lead to well-known problems in health informatics, which are related to logical data representation, aggregation, data analysis, data standardization, and data interoperability [7,8]. Targeted personal, habitual, physiological, activity, and nutrition data are generally collected via secure wearable sensors, manual interactions, interviews, web-based interactions, smartphone apps, customized questionnaires, and feedback forms over time. Weather application programming interfaces (APIs) and external weather sensors are useful for the collection of contextual weather data over time. The wearable activity monitors need to connect to a personal smartphone via Bluetooth nearfield communication technology (Bluetooth low energy [BLE]) [9,10]. The device can seamlessly measure and transfer high-resolution raw acceleration data and multiple activity parameters to a secure storage to process the data further with a machine intelligence module [11]. High-end, time-dependent activity data collection with wearable BLE devices has become accessible and feasible for ubiquitous monitoring. Some of the activity data, such as nonwear time or intensive activity details, are questionnaire dependent.

Physiological data are collected either invasively (eg, glycemic response, cholesterol level) or noninvasively (eg, weight, BP, heart rate, body assessment data). The questionnaire-dependent nutrition data are collected either daily or on an alternate day or on a weekly basis. The assessment of nutrition data helps to determine the type of food, amount of food, conceptual information (temporal/spatial), dietary pattern, and intake of alcohol or energy drinks. Some baseline data (medical history, habit, preference, personal details, initial weight and height, initial BP, and initial body assessment data) are collected during the initial recruitment of the participant or every month for either demographic statistics or population clustering or individual goal assessment. Each data have their unit and range following a standard guideline based on the context and domain (eg, data on temperature are applicable for both health and environment domain with a different range, meaning, and context). Therefore, each measurement process owns separate challenges related to logical or semantic data representation, proper usage of data, and improving data reusability. The data usability involves the transformation of data into an understandable computer format. It creates a challenge to systematically and syntactically analyze health and wellness data in aggregation with other clinical data. Incorporation of physical activity, diet as a care procedure, or investigating how it afflicts healthy outcomes involves a more detailed and diverse representation of participant’s behavioral level and physiological condition [7,8,12,13].

Furthermore, the challenges of reusing the existing physiological and behavioral data of a participant within the electronic health record remain and include concerns related to opacity and semantic inconsistency [7,8]. Besides, these health and wellness data are still mostly hidden in clinical narratives with highly variable forms of expression. In this regard, ontology can provide a framework to allow the mentioned heterogeneous health and wellness data to be organized, compact, structured, consistent, machine understandable, and queried through high-level specifications. Ontology helps to annotate diverse health and wellness data with semantic metadata to increase interoperability among heterogeneous networks, data integration, discovery, and situation awareness. An eHealth ontology can reuse the concept of existing, proven, well-accepted ontologies (eg, semantic sensor network [SSN] ontology [14], Systematized Nomenclature of Medicine—Clinical Terms [SNOMED CT] ontology [15]) to enhance its vocabularies and better semantic representation.

A rule-based decision support system (DSS) can use such an eHealth ontology model to measure and predict health risks, and to generate useful personalized recommendations following proven clinical rules. If the collected health and wellness data are not annotated accurately with semantic metadata in the medical domain, then the DSS may fail to deliver accurate decisions to both physicians and patients or participants in the form of incorrect recommendation plan, goal setting, and goal evaluation. DSS decision inaccuracy may appear primarily due to the following effects—improper design of knowledge base (KB), the inadequacy of tools or technologies applied in the execution of DSS, problems related to the ontology reasoning engine, and issues associated with inferring new knowledge.

### Aim of the Study

After studying existing ontology models, we found that many ontologies and regulated terminologies cover aspects of obesity and related chronic illness domains, but concept analysis remains incomplete. After reviewing relevant ontologies, we proposed a freshly created OWL-based ontology to deal with different data inputs (internet of things [IoT] sensors, interviews, and questionnaires) and annotate them with semantic data. The proposed ontology will support data interoperability, logical representation of collected health and wellness data in context, and to build a rule-based DSS for health risk prediction related to obesity and afterward generation of lifestyle recommendations for a healthy lifestyle.

We have not evaluated the impact of the suggested recommendations on participants as we executed the complete scenario under a simulated environment. Still, we evaluated the performance of the proposed ontology model. In the proposed ontology, we annotated every participant’s data with semantic web language rules and stored the generated OWL file in a triple-store format for better readability (Multimedia Appendix 1). The proposed ontology model allows automatic inferencing, efficient knowledge representation, balancing a trade-off between complexity and eloquence, and reasoning about formal knowledge. The entire study is divided into the following 2 segments: (1) ontology design and development and (2) its verification. This study addresses the following identified research questions:

*(RQ-1) How to annotate distributed, heterogenous health and wellness data received from sensors, questionnaires, and interviews into meaningful information to build a future machine learning model for health risk prediction for obesity?*

*(RQ-2) How to integrate existing IoT and medical ontologies to design and develop proposed eHealth ontology for obesity study case?*

*(RQ-3) How to verify the proposed ontology with rule-based behavioral recommendation generation?*

For this set of semantic data, which will be considered as asserted true facts, the primary goal of the paper is to trigger logical rules of the shape (A IMPLIES B) or trigger rules in a logically equivalent way, that is, (NOT(A) OR B). If some specific variables are inferred to be true, then some recommendations shall be provided to the user from whom the semantic data are originating.

### Related Work

This section offers existing background knowledge applicable for this research. It includes (1) a discussion of existing, relevant eHealth ontology models for chronic illness, health monitoring, and ontology-based DSS, (2) ontologies in the IoT domain for modeling sensor data, and (3) ontologies in the medical domain.

#### Existing eHealth Ontology Models

Different research groups have conducted different studies on eHealth ontology modeling for chronic illness, health monitoring, and ontology-based clinical decision support system (CDSS). For example, Kim et al [16] developed an ontology model for obesity management with the nursing process in the mobile device domain for spontaneous participant engagement and continuous weight monitoring. The scope of the obesity management included behavioral interventions, dietary recommendations, and physical activity, and for this purpose, the study included assessment data (BMI, sex, and hip-to-waist circumference), inferred data for representing diagnosis results, evaluations (cause of obesity, success, or failure of behavioral modifications), and implementation (education, suggestion, intervention). Sojic et al [17] modeled an obesity domain–specific ontology with OWL to design inference patterns to individualize health condition assessment as age and gender specific. The ontology helped classify personal profiles based on the changes of personal behavior or feature over time and infer personal health status automatically, which are important for obesity evaluation and prevention. The ontology rules were written in semantic web rule language (SWRL). Kim et al [18] proposed an ontology model for physical activity (PACO) to support physical activity data interoperability. The ontology was developed in Protégé (version 4.x), and the FaCT++ reasoner verified its structural consistency. Lasierra et al [19] developed an automatic ontology–based approach to manage information in home-based scenarios for telemonitoring services based on the automatic computing paradigm, namely, MAPE (monitor, analyze, plan, and execute). They proposed another 3-stage ontology-driven solution [20] (stage 1: ontology design and implementation; stage 2: ontology application to study personalization issues; and stage 3: software prototype implementation) for giving personalized care to chronic patients at home. The proposed ontology was designed in OWL DL language in Protégé-OWL version 4.0.2 ontology editor and was verified using FACT++ reasoner. The ontology development involved data from heterogeneous sources, such as clinical knowledge, data from medical devices, and patient’s contextual data. Yao and Kumar [21] proposed a novel CONFlexFlow (Clinical cONtext based Flexible workFlow) approach using ontology modeling for incorporating flexible and adaptive clinical pathways into CDSS. They developed 18 SWRL rules for practical explanation of heart failure. The model was verified with the Pellet Reasoner Plug-in for Protégé version 3.4. Additionally, they developed a “proof-of-concept” prototype of the proposed approach using the Drools framework. Chi et al [22] constructed a chronic disease dietary consultation system using web ontology language (OWL) and SWRL. The KB involved heterogeneous sources of data and interaction of factors, such as the illness stage, the physical condition of the patient, the activity level, the quantity of food intake, and the critical nutrient constraints. Rhayem et al [23] proposed an ontology-based system (HealthIoT) for patient monitoring with sensors, radiofrequency identification devices, and actuators. They claimed that data obtained from medically connected devices are enormous, and thereby lack repressibility and understandability, and are manipulated by other systems and devices. Therefore, they proposed an ontology model to represent both the connected medical devices and their data based on a semantic rule, followed by model evaluation with the proposed IoT Medicare system that supports decision making after analyzing the vital signs of the patients. Galopin et al [24] proposed an ontology-based prototype CDSS to manage patients with multiple chronic disorders following clinical practice guidelines. The KB decision rules were based on the “if–then” rules following clinical practice guidelines and patient observation data. Sherimon et al [25] proposed an ontology system (OntoDiabetic) using OWL2 language to support a CDSS for patients with cardiovascular disease, diabetic nephropathy, and hypertension following clinical guidelines and “if–then” decision rules. Hristoskova et al [26] proposed another ontology-driven ambient intelligence framework to support personalized medical detection and alert generation based on the analysis of vital signs collected from the patients diagnosed with congestive heart failure. The DSS system can classify personalized congestive heart failure risk stages, and thereby, notify patients through ambient intelligence’s inference engine. Riaño et al [27] proposed an ontology-based CDSS for monitoring and intervening chronically ill patients to prevent critical conditions, such as incorrect diagnoses, undetected comorbidities, missing information, and unobserved related diseases. Jin and Kim [7] designed and implemented an eHealth system using the IETF YANG ontology based on the SSN concept. The approach assisted in the autoconfiguration of eHealth sensors (responsible for collecting body temperature, BP, electromyography, and galvanic skin response) with the help of internet and communication technologies and querying the sensor network with semantic interoperability support for the proposed eHealth system. The proposed eHealth system consisted of 3 main components: SSN (eHealth sensors, patient, unified resource identifier [URI]), internet (eHealth server, KB), and eHealth clients (patient, and professionals). The proposed semantic model used a “YANG to JSON translator” to convert YANG semantic model data to JSON semantic model data for semantic interoperability before storing them in the database (KB). Ganguly et al [28] proposed an ontology-based model to manage semantic interoperability problems in eHealth in the context of diet management for diabetes. The development of the framework included rules of dialogue games, DSS with KB (rule base and database), a dialogue model based on decision mechanism, the syntax of dialogue game, decision mechanism, and translational rules.

#### Ontologies in the Internet of Things Domain

Ontology [29] provides a framework for describing sensors. SSN-XG (W3C Semantic Sensor Network Incubator Group) developed the SSN ontology to model sensor devices, systems, processes, and observations. SSN annotates sensor data with semantic metadata (semantic sensor web) to increase interoperability among diverse networks, data integration, discovery, and situation awareness. The Sensor Model Language (SensorML) was developed by the Open Geospatial Consortium (OGC), which provides syntactic descriptions using XML to describe sensors, observations, and measurements. While SensorML provides an XML schema for defining sensors, it lacks the repressibility provided by ontology languages such as OWL [30-32]. Semantic sensor web, a combination of sensor and semantic web technologies, helps to annotate spatial, temporal, and thematic semantic metadata for the more artistic representation of sensor data, advanced access, formal analysis of sensor resources, and data standardization. SSN ontology is used to describe sensor devices; sensing; sensor measurement capabilities; and sensor observations, process, and systems [30-32]. SSN allows its network, sensor devices, and data to be installed, structured, managed, queried, and controlled through high-level specifications. Sensors Annotation and Semantic Mapping Language offers XML schema to transfer sensor data and sources into the instances of SSN ontology based on a predefined XML-based document (resource description framework [RDF]), which is achieved automatically with sensor data to RDF mapping algorithm [33]. “M3 Ontology” (machine-to-machine) was developed based on the “SenML” protocol (designed for simple sensor measurement), which is an extension of SSN, to enable the interoperable design of domain-specific or cross-domain-specific applications which are termed as Semantic Web of Things [13]. AeroDAML, KIM, M3 Semantic Annotator, MnM, and SemTag are different available semantic annotators for sensor observations for their corresponding semantic models (DAML, KIMO, M3, Kmi, and TAP) [34]. Like SSN, there are other IoT-based contextual ontologies, such as IoT-Ontology, IoT-Lite, and IoT-O [35]. SCUPA, CoBrA-Ont, CoDAMoS, PalSPOT, the delivery context ontology, and Fuzzy-Onto are different IoT-based ontologies for activity recognition [34]. URI, HTTP, HTML5, REST, SOAP, Web Socket, Web feed, MQTT, CoAP, and AMQP are some standard IoT protocols applicable to Web of Things [14,34,36,37]. In this study, we integrated the concept of SSN ontology to model sensor observations.

#### Ontologies in Medical Domain

SNOMED CT, 11th edition of the International Classification of Diseases (ICD-11), Unified Medical Lexicon System (UMLS semantic network), Foundational Model of Anatomy, OpenEHR, Gene Ontology, DOLCE, Basic Formal Ontology, Cyc’s upper ontology, Sowa’s top-level ontology, the top level of GALEN, and Logical Observation Identifiers Names and Codes (LOINC) are biomedical ontologies introduced to deliver semantic interoperability and complete knowledge related to the specific biological and medical domains [38]. Most laboratory and clinical systems send out data using the HL7 (version 2) protocol and in an HL7 message, the LOINC codes represent the “question” for a laboratory test or experiment and the SNOMED CT code represents the “answer.” In this study, we have reused the SNOMED CT ontology for modeling the health condition based on health and wellness data, and recommendation generation [8]. SNOMED CT was designed in 1965 as a controlled medical vocabulary licensed and supported by the International Health Terminology SDO. It is an organized list of a wide variety of clinical terminology defined with unique codes (ICD). It covers a wide range of medical terminologies for disorders and findings (what were observed!), procedures (what was done!), events (what happened!), substance/medication (what was consumed or administered!), and anything related to medical data. It offers a shared language that enables a reliable way of indexing, storing, reclaiming, and accumulating clinical data across fields and care sites. It is a complete, multilingual clinical terminology that gives clinical content and clarity for clinical documentation and reporting [8,38,39].

As described above, most studies have developed ontologies using OWL to solve the data interoperability problem. Still, integration among the electronic health data, semantic rules, semantic annotation, clinical guidelines, health risk prediction, and personalized recommendation generation remains an issue in eHealth. This study addresses it and proposes a prototype ontology model for obesity as a case study, to integrate data from heterogeneous sources (eg, sensor, questionnaire, and interview) in order to enable data interoperability, information search and recovery, and automatic interference. We integrated SSN and SNOMED CT ontologies into our proposed eHealth ontology because of their vast vocabularies, appropriateness, and semantic capabilities as discussed above [40-43].

## Methods

### Basics of Ontology

Ontology commenced as a philosophical discipline studying the existence and being and expanded into information technologies. Ontology is a formalized model for specific domains with the following essential elements: individuals/objects, classes, attributes, relations, and axioms. A class diagram of a program written in object-oriented programming [44] is a visual representation of an ontology. Ontology is a philosophy that has been around for thousands of years, and it allows for design flexibility by reusing existing ontologies [45]. It follows the open world assumption knowledge representation style using OWL, RDF, and RDF schema (RDFS) syntaxes. It can be optimized with ontology patterns, and its logical and structural consistency is verified with ontology reasoners.

### Overview

**Figure 1:**
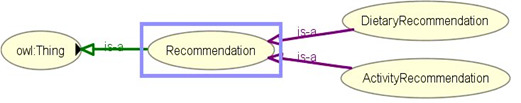
Asserted hierarchy for lifestyle recommendation for obesity management.

The proposed eHealth ontology encompasses the following steps: (1) ontology design approaches and used vocabularies; (2) ontology modeling in Protégé; (3) defining the scope; (4) integrating existing IoT and medical ontologies in the proposed ontology to annotate sensor and clinical observations; (5) ontology implementation (mapping the concepts to the proposed ontology classes and their properties in Protégé); and (6) rule expression (rule base) and basic SPARQL queries as a part of ontology verification. We further discuss how rule-based lifestyle recommendation messages (regarding activity and nutrition) could be delivered to the participants following an asserted hierarchy in the proposed eHealth ontology model, as depicted in Figure 1.

### Ontology Design Approaches and Used Terminologies

Ontology design approaches can be classified into the following 5 categories: inspirational, inductive, deductive, synthetic, and collaborative [46]. We adopted a combination of inspirational and deductive approaches in our ontology design and development. The inspirational approach helped us identify the need for the ontology (what to design?) and obtain expert views to create the ontology (how to design?). The deductive approach helped us to adopt and adapt general principles to create the intended ontology tailored toward obesity as a study case. It includes the general notions being filtered and refined to be personalized to a specific domain subset (obesity). The overall approaches are divided into 5 phases as follows: in phase 1, we performed a systematic literature review to understand the need for an ontology to support the logical representation of observable and measurable data for healthy lifestyle management targeting obesity as a case study. In phase 2, we consulted experts with a research background in ICT, eHealth, nursing, and nutrition for designing the ontology. In phase 3, we developed the ontology to model and annotate health and wellness data observations with semantic metadata to create a lightweight, intelligible abstraction for health risk predictions for the personalized generation of recommendations based on rule-based decision making. In phase 4, we created rules for SPARQL queries and personalized recommendation generation (rule-based deduction). In phase 5, we verified the ontology with simulated data based on rule-based decision support.

The semantic web is W3C recommended, and it allows the specification of metadata that permit automatic reasoning [47,48]. The W3C-maintained specifications related to this study are XML, URI, RDF, turtle, RDFS, ontology web language (OWL), SPARQL Protocol and RDF Query Language (SPARQL), and SWRL. The following terminologies are relevant for our eHealth ontology representation and processing: propositional variable (an atomic name of a truth value that may change from one model to another), constant (the unique propositional variables TRUE and FALSE such that their truth value cannot be changed), and operators (the set of logical connectors in each logic). Besides, in this case, we use the operators (NOT, AND, OR, IMPLIES, and EQUIV); quantifiers (the set of logical quantifiers in a given logic; FORALL for the universal quantifier and EXISTS for the existential quantifier); quantified clause (a set of propositional variables linked together by operators and quantifiers); clause (a quantified clause without any quantifiers); formula (a collection of clauses and quantified clauses related together by logical operators); and model of the procedure (a group of assignments for each propositional variable, such that when simplified, it leads the procedure to the constant TRUE).

Protégé, TopBraid Composer ($), NeOn Toolkit, FOAF editor, WebOnto, OntoEdit, Ontolingua Server, Ontosaurus, and WebODE are some popular ontology editors [49]. These ontology editors are open-source ontology development tools with OWL support. A reasoner is a crucial component for working with OWL ontologies. It derives new truths about the concepts that are being modeled with OWL ontology. Practically all querying of an OWL ontology (and its import closure) can be done using a reasoner [50,51]. That is why knowledge in an ontology might not be explicit, and a reasoner is required to deduce implicit knowledge so that the correct query results are obtained. The OWL API includes various interfaces for accessing OWL reasoners. For accessing reasoner via the API, a reasoner implementation is necessary. Reasoners can be classified into the following groups: OWL DL (Pellet 2.0*, HermiT, FaCT++, RacerPro), OWL EL [CEL, SHER, snorocket ($), ELLY], OWL RL [OWLIM, Jena, Oracle OWL Reasoner ($)], and OWL QL (Owlgres, QuOnto, Quill) [50-57]. In this study, we utilized Protégé ontology editor and HermiT reasoner to create and validate the structure of the ontology.

Apache Jena is a Java-based framework used for building semantic web applications. It provides an API to extract data from and write to RDF graphs. A Jena framework includes the following: (1) RDF API to parse, create, and search RDF models in XML, N-triple, N3, and Turtle formats. Triples can be stored in memory or database; (2) ARQ Engine/SPARQL API, which is a query engine for querying and updating RDF models using the SPARQL standards; (3) tuple database engine as a high-performance RDF store on a single machine; (4) ontology API for handling OWL and RDFS ontologies; and (5) Apache Jena Fuseki, which is the SPARQL server for supporting query and update. It is tightly integrated with tuple database to deliver a robust, transactional persistent storage layer. The framework has internal reasoners and an OWL API [58,59]. In this study, we used Apache Jena Fuseki for SPARQL processing with triple database.

Knowledge representation in computer-understandable form is well accepted among AI communities. Knowledge representation with symbols facilitates inferencing and the creation of new elements of knowledge. By contrast, the KB is a database for knowledge management. It provides a means for information to be collected, organized, shared, queried, and utilized for inferring new information. Knowledge engineering helps to obtain specific knowledge about some subject and represents it in a quantifiable form. KB consists of terminology models or TBox (atomic and complex) and assertions model instance or ABox (asserted and inferred). Inferred statements come as a logical outcome of the asserted statements and logical rules [35,60,61]. A KB is a pair (T, A) where T is a TBox and A is an ABox. The idea behind this paper is that the TBox concepts and relations are coming from the freshly created ontology and the ABox is a list of clauses assigning truth values to some variables. The TBox is coming from integration with the SSN Ontology and the SNOMED CT ontology plus additional concepts specific to the recommendation test case considered. The ABox is the semantic data, coming from the different data inputs (IoT sensors, interviews, and questionnaires). The satisfiability of the KB, and thus the model output, is obtained by using the hyper-tableau-based [62] reasoning solver HermiT [55]. The whole approach has been tested with 4 generated test cases to ensure that the whole mechanism can indeed set the propositional variables to true and thus send the corresponding recommendation message when needed.

### Ontology Modeling

An ontology can be modeled with the following 2 ways in Protégé: frame based and OWL based. The Protégé frame editor ensures ontology development following the Open Knowledge Base Connectivity Protocol with the help of classes, properties, relationships, and instances of classes (objects). By contrast, the Protégé OWL editor (applied in this study) enables ontology development for the Semantic Web with the help of classes, properties, instances, and reasoning. We have used the steps detailed in Textbox 1 to model our proposed OWL-based eHealth ontology using the Protégé OWL editor.

**Steps to model the proposed OWL-based eHealth ontology.**

**Step 1**

Create a new empty OWL project in Protégé and save it as a local file with “owl” or “ttl” extension (“ttl” signifies the turtle resource description framework [RDF] format).

**Step 2**

Create named classes under the “owl:Thing” super class following consistency

- Create a group of meaningful and required classes
- Define disjoint classes
- Define subclasses and disjoint subclasses

**Step 3**

Create OWL properties

- Object properties (associates object to object)
- Data properties (relates object to XML schema datatype or rdf:literal)
- Annotation properties (to add annotation information to classes, individuals, and properties)

**Step 4**

Define object properties if they are subproperties, inverse properties, functional properties, inverse functional properties, transitive properties, symmetric properties, and reflexive properties.

**Step 5**

Define property domain and ranges for both object and data properties (it is used as axioms in reasoning).

**Step 6**

Define property restrictions as follows:

- Quantifier restrictions (existential and universal)
- Cardinality restrictions (one or many)
- hasValue restrictions (eg,, string/integer/double)

**Step 7**

Ontology processing with a reasoner to check consistency in OWL DL, and to compute the inferred ontology class hierarchy.

- Blue color class in the inferred hierarchy signifies that the class has been reclassified.
- Red color class in the inferred hierarchy signifies an inconsistent class.

**Step 8**

Remove inconsistencies before importing the ontology file in Apache Jena for further processing, querying (Simple Protocol and RDF Query Language [SPARQL]), and storing it into tuple database for persistence. Tuple database supports the full range of Jena application programming interfaces. It can be used as a high-performance RDF store on a single machine.

### Scope of the Proposed Ontology

**Figure 2:**
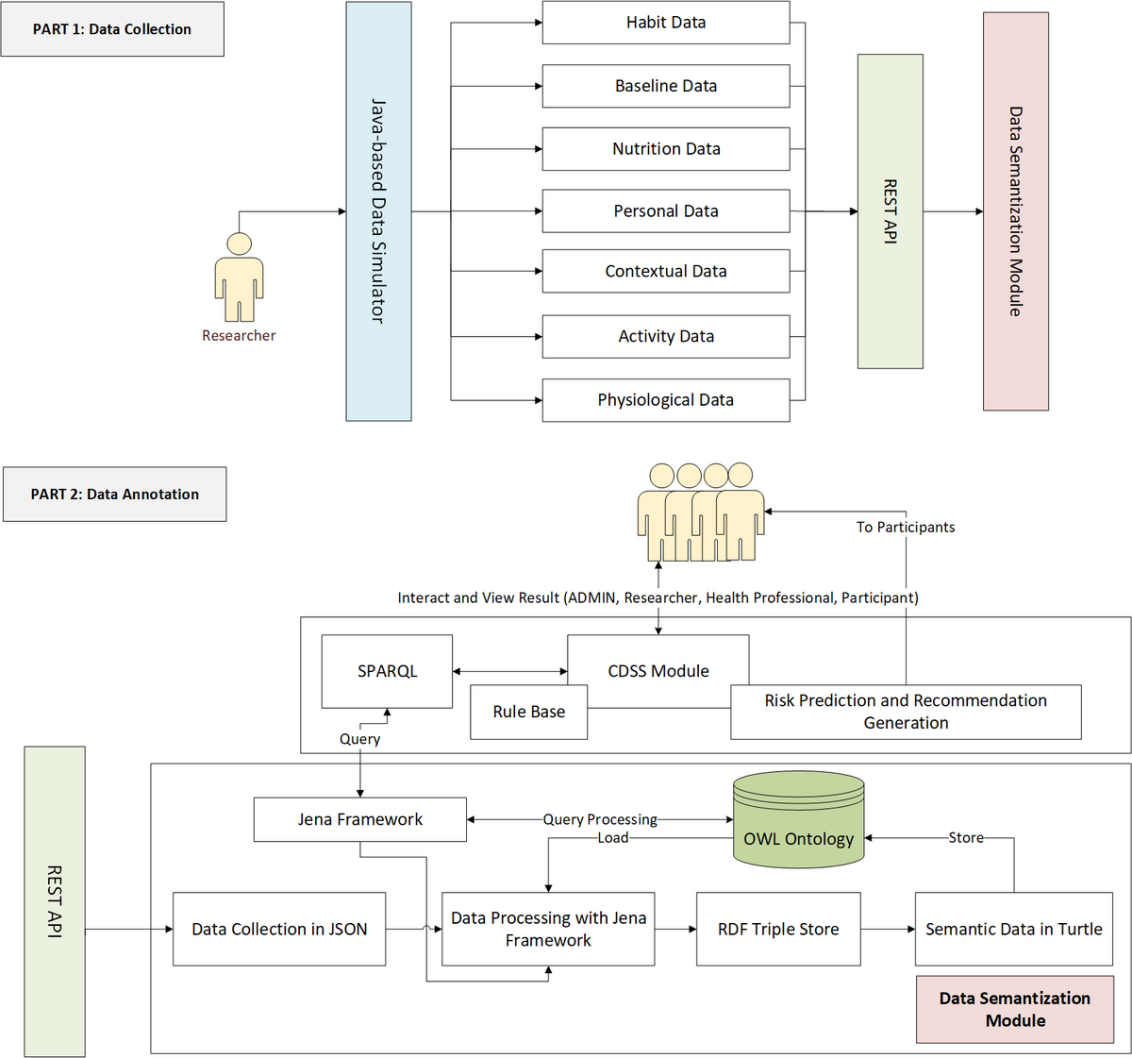
Proposed eCoach system architecture for data semantization.

**Figure 3:**
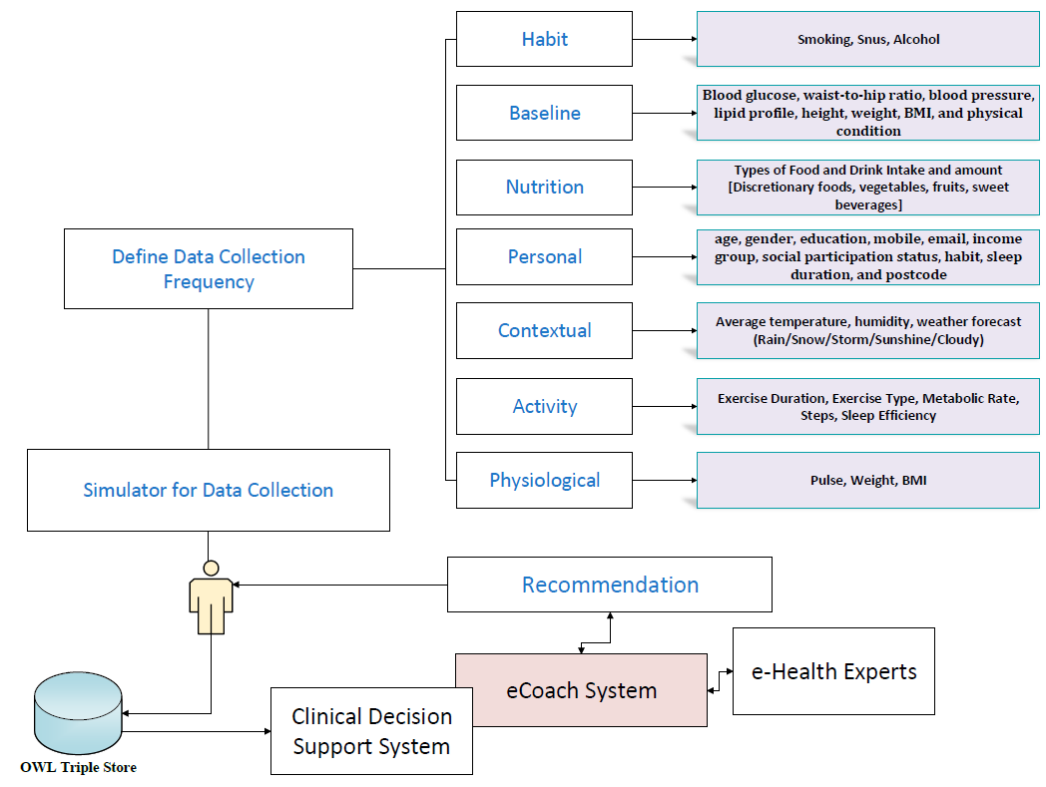
Types of data to be collected from participants.

We have planned to integrate the proposed eHealth ontology into a simulated eCoach system used for automatic rule-based recommendation generation to inspire individuals to manage healthy lifestyles with early health risk predictions. The planned system will have 2 main modules, as depicted in Figure 2: a data collection module and a data annotation module. The data collection module will collect identified fabricated set of habit, baseline, nutrition, personal, contextual, activity, and physiological data over time via a simulator, as depicted in Figure 3.

**Figure 4:**
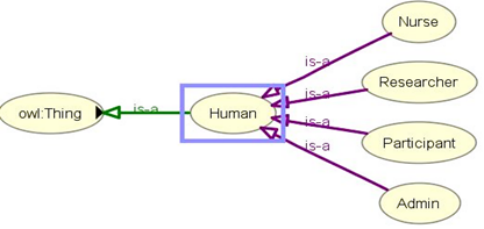
Different types of users involved in the eCoach System.

The accumulated data were annotated with semantic metadata (RDF triple store graph) and stored in tuple database in turtle format. The DSS, rule base, SPARQL, risk prediction, and recommendation generation modules are not the core, and they are used for ontology verification as a test engine. The scopes of DSS are as follows: (1) periodic querying of the ontology with Jena framework using preset SPARQL queries [63-65] to assess the health condition; and (2) mapping the query result to preset clinical rules in “rule base” to generate lifestyle recommendations. This study involves 4 different user types: administrator, researcher, participants, and health professionals (eg, nurses; Figure 4). The ontology is protected from personal identity disclosure as no unique identifiers (eg, national identifiers) of participants were collected and stored in the simulated environment in accordance with the Norwegian Centre for Research Data guidelines [66]. Core eCoach and DSS concepts, AI integration for health and wellness data (activity and nutrition) analysis, real-world data collection from actual participants through web applications/mobile apps, real-life personalized recommendation generation, goal evaluation, pregnancy, genetics, child obesity, and obesity in older adults are beyond the scope of this study. This study’s primary focus is to design and develop an eHealth ontology for the obesity case and to verify it with artificial data and behavioral recommendation generation with a rule-based DSS. Defined rules for test setup may vary with change in the context and is not the key focus of this paper.

We simulated habit, nutrition, contextual, activity, and physiological data for 4 dummy participants (2 healthy weight [N] and 2 overweight [O] participants aged between 18 and 40) for the very first day (day-n; n>0); see Multimedia Appendix 2. We assumed all the dummy participants are from the same region, so the contextual information is the same. Rule-based recommendations based on data analysis on “day-n” will be carried out by targeted participants on “day-(n+1).” Recommendations inform individual participants about their daily activity (sedentary or not), dietary intake, and activity/dietary plans. For dietary assessment, we have relied on the daily self-reported questionnaire, rather than on direct calorie calculation for basal metabolic rate. Baseline data help to compare (at the end of each month until the process ends) whether any improvement or deterioration occurred as a result of behavior change based on lifestyle recommendations. For example, reduction in BMI and BP for a person who is obese/overweight, and maintaining safe BMI and BP for a person with healthy weight upon following the behavioral recommendations is a good indication of maintaining a healthy lifestyle. We consulted with 5 experts with a research background in ICT, eHealth, nursing, and nutrition for simulating activity and nutrition data. Obesity-related information and guidelines were obtained from the World Health Organization (WHO) [67], the National Institute for Health and Care Excellence (NICE) [68], and the Norwegian Dietary Guidelines [69].

### Integration With SSN Ontology and SNOMED CT

**Figure 5:**
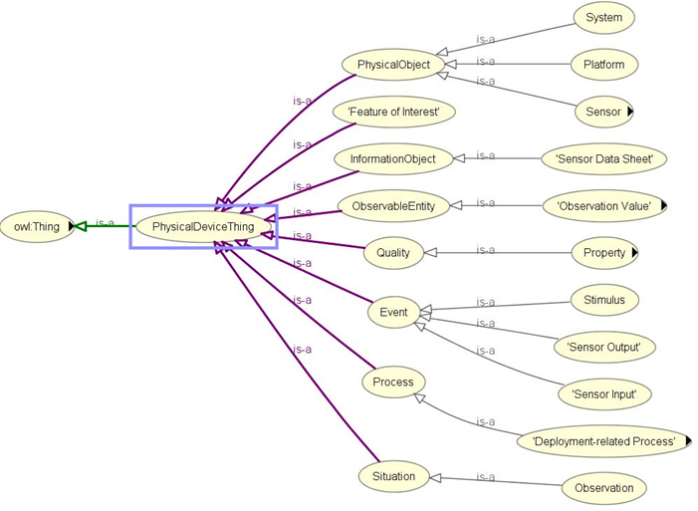
Asserted hierarchy for sensor-based data collection with OWLViz.

We integrated the SSN ontology [30,36,70-72] into our proposed eHealth ontology to describe sensors (activity sensors and external weather sensors), their observations, and methods adopted for sensing individual activities and context (Figure 5). Observation data related to activity and external weather are annotated with SSN ontology concepts and object properties.

Concepts and object properties in the ontology are commented and connected with “rdfs:label,” “rdfs:isDefinedBy,” “rdfs:seeAlso,” “rdfs:comment,” “dc:source,” “isProxyFor,” “has value,” “is produced by,” “has property,” “hasTimeStamp,” “isRegionFor,” “attached system,” “in deployment,” “has measurement capability,” “detects,” “hasOutput,” “observes,” “implements,” “has deployment,” “has operating range,” “has subsytem,” “has survival range,” “on platform,” “deployment process part,” “deployed on platform,” “deployed system,” “is property of,” “feature of interest,” “observation result time,” “observation sampling time,” “observed property,” “quality of observation,” “sensing method used,” “includesEvent,” and “observedBy.” The SSN ontology is constructed on the foundation of a central ontology design pattern, so-called the stimulus–sensor–observation pattern to describe relationships between sensors, stimulus, and observations [30], and the same concept is reused in our proposed eHealth ontology model. The perspectives of SSN ontology can be classified as follows [30]: a sensor perspective, an observation perspective, a system perspective, and a feature and property perspective. Namespaces for the SSN and DUL ontologies are reused in our ontology prefixing concepts and properties as ssn: and dul:, respectively. “PhysicalDeviceThing” (a class), which behaves as a superclass of classes related to sensor-based observations, is a subclass of “owl:Thing,” the universal ontology superclass.

**Figure 6:**
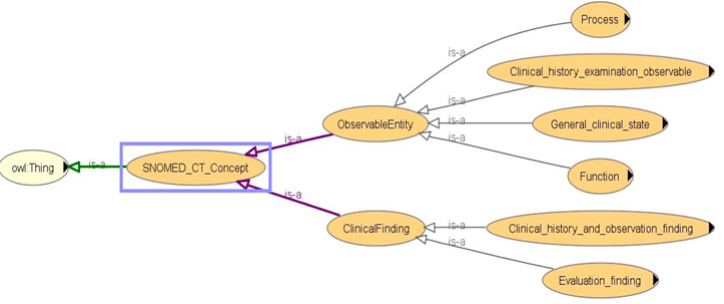
Asserted hierarchy of SNOMED_CT concept with OWLViz.

We incorporated selected concepts from SNOMED CT [73] into our proposed ontology model to define how information about the participant’s state is to be structured and processed. The SNOMED CT ontology combines hierarchical “is-a” relationships and other related relationships for vital signs, process, body measurements, and observations to describe clinical attributes as depicted in Figure 6. SNOMED CT simplifies the search for respective diseases, process, function, clinical state, measurements, and vital signs, and every concept is identified with an SCTID or SNOMED CT identifier with an object property “hasSCTID” (eg, Obese_finding hasSCTID value “414915002”^^xsd:long) [74].

**Figure 7:**
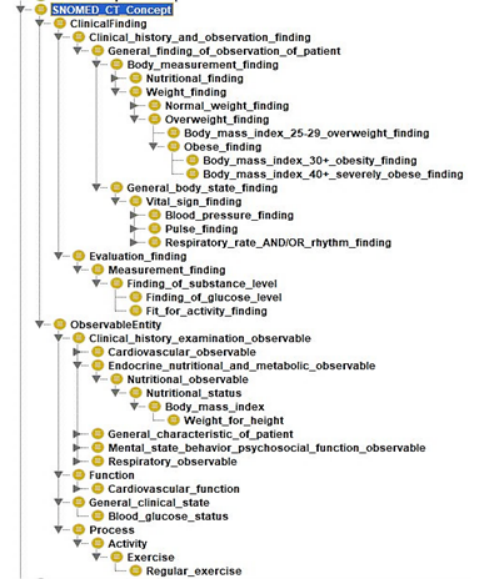
SNOMED CT class hierarchy based on selected concepts.

**Figure 8:**
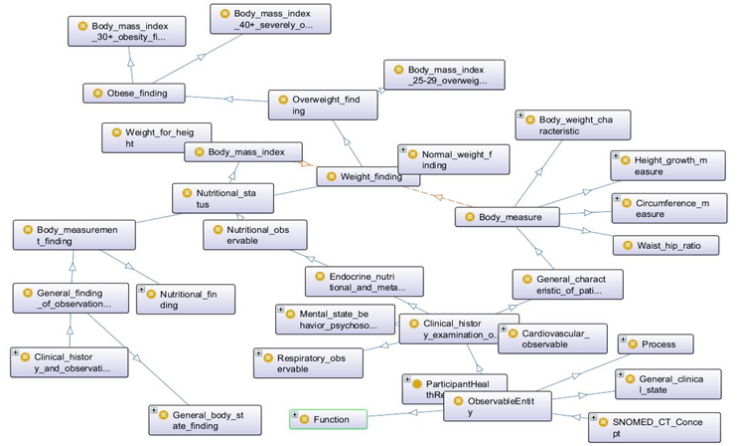
SNOMED CT ontology visualization with OntoGraf based on selected concepts.

**Figure 9:**
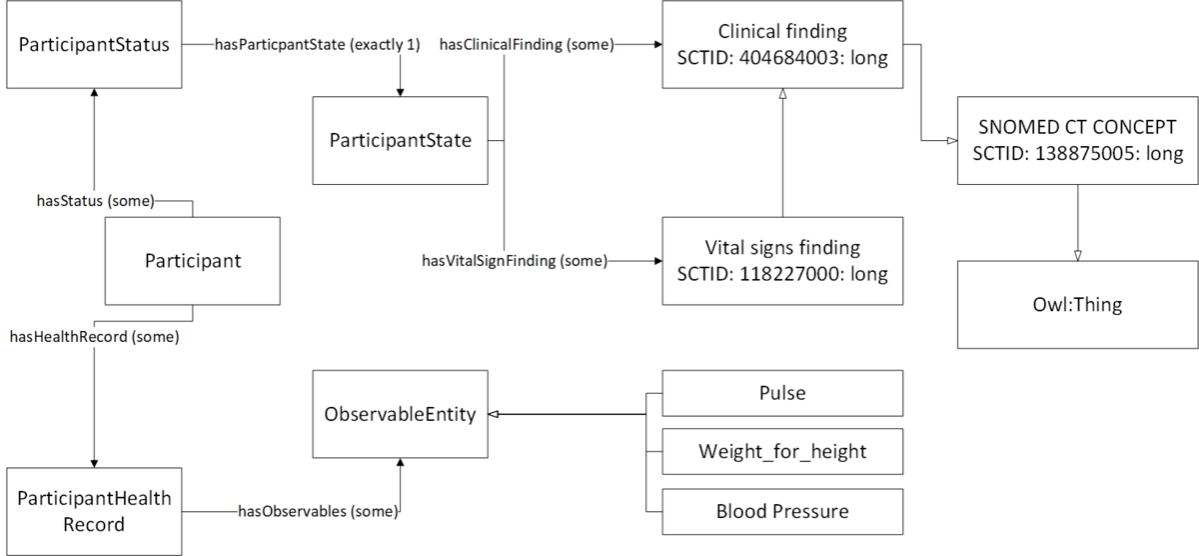
Selected concepts from SNOMED CT Ontology for vital signs, body measurement, and observations.

Figures 7-9 describe the class hierarchy to process participant’s clinical information using the SNOMED CT hierarchy for the vital signs (eg, BP, pulse) and body measurement (eg, obese or overweight) based on the observable entities [75-79]. Observable entities and clinical findings are linked with the objectProperty: isFoundBy. The proposed ontology model can be extended for additional clinical findings [73,74].

### Ontology Implementation

**Figure 10:**
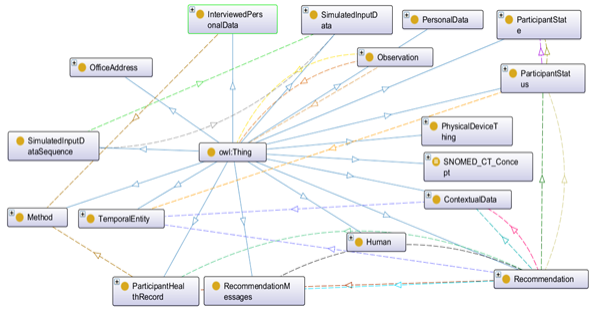
Proposed eHealth Ontology implementation in Protégé 5.x.

**Figure 11:**
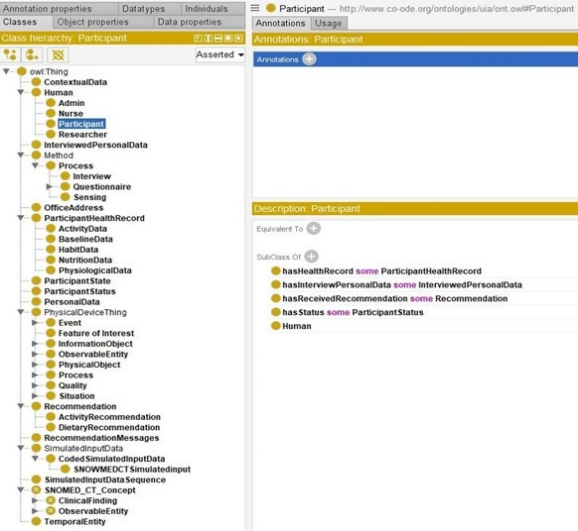
The class hierarchy of the proposed eHealth ontology and the description of participant class.

**Figure 12:**
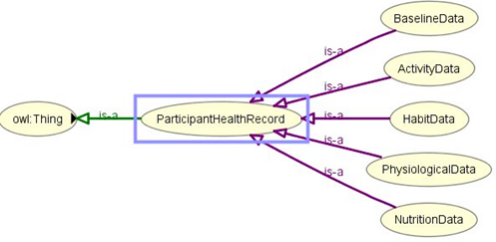
The asserted class hierarchy of participant’s health record with OWLViz.

**Figure 13:**
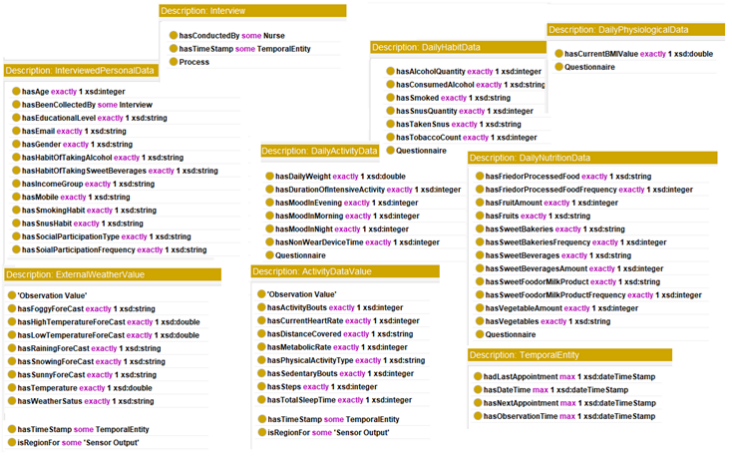
Data properties related to data collection.

In Figure 10 we describe how we implemented the proposed eHealth ontology for our future eCoach system with required classes, object properties, and data properties to annotate collected data. The administrator, health professionals, researchers, and participants are subclasses of the “Human” class. They have their designated role, password, and userId to authorize themselves in the system with the following associated objectProperties: hasRole, hasPassword, and hasUserId, respectively. Administrator, health professionals, and researchers have their office address (hasOfficeAddress), and personal data (hasPersonalData) to describe themselves. Their office address consists of a phone number, a postcode, and a room number with the following associated dataProperties: hasOfficePhone, hasOfficePostCode, and hasRoomNo, respectively. Their personal data include age, designation, email, first name, last name, gender, and mobile number with the corresponding dataProperties hasAge, hasDesignation, hasEmail, hasFirstName, hasLastName, hasGender, and hasMobile. The “Participant” is an important class and participants are at the core of the system. Participants have their health record, personal data obtained through interview process by trained health professionals, status (active/inactive), and recommendation with the associated objectProperties hasHealthRecord, hasInterviewPersonalData, hasStatus, and hasReceivedRecommendation as depicted in Figure 11. “ActivityData,” “BaselineData,” “HabitData,” “NutritionData,” “PhysiologicalData” are subclasses of the “ParticipantHealthRecord” class as depicted in Figure 11. Activity data are an observable entity and are planned to be collected via activity sensors (activity bouts, steps, sleep time, activity duration, sedentary bouts, metabolic rate, nonwear time) and questionnaire (duration of intensive activity and nonwear sensor time) daily. Intensive activities are running, weightlifting, cycling, swimming, and skiing. Based on the activity type, participants can be classified into the following 4 groups: sedentary, light active, moderate active, and active. Baseline data (blood glucose, waist-to-hip ratio, BP, lipid profile, height, weight, BMI, and physical condition) are planned to be collected by trained health professionals at the time of recruitment of participants and on a monthly basis following an interview process. Habit data (smoking, snus, and alcohol consumption) and nutrition data (types of foods and drinks with amount) are planned to be collected daily with a pre-set questionnaire. Physiological data (pulse, weight, and BMI) are planned to be collected daily via activity sensors and pre-set questionnaire, as depicted in Figure 12. Personal data (age, gender, education, mobile, email, income group, social participation status, habit, sleep duration, and postcode) of healthy participants are planned to be collected following an interview process by trained health professionals during recruitment. Gender, education, income range, and social participation are essential for demographic classifications. The data properties related to data collection are depicted in Figure 13.

**Figure 14:**
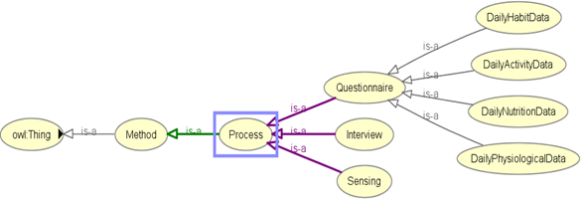
The asserted class hierarchy of participant’s data collection methods with OWLViz.

**Figure 15:**
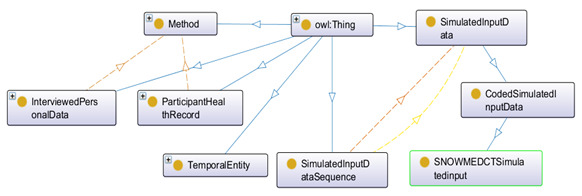
Ontology for data collection from simulated input.

The asserted class hierarchy of the methods used for participant’s data collection is depicted in Figure 14. Each method ensures a collection of simulated data sequences, maintaining a timestamp as depicted in Figure 15. Contextual data are observable weather-related data (weather status, current temperature, rain forecast, snow forecast, storm forecast, sunny forecast, high and low temperature forecast, fog forecast), which are planned to be collected daily via sensing devices. The relationship between data and data collection methods are linked with the objectProperty: hasBeenCollectedBy and hasConductedBy (for interview).

**Figure 16:**
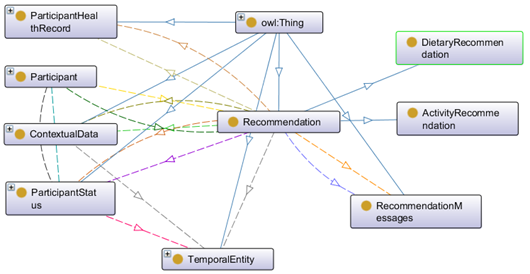
Ontology for recommendation generation.

Behavioral recommendations for a healthy lifestyle can be classified in the following 2 categories: activity (A) and dietary (D). Each recommendation is personalized and contextual. Therefore, the recommendation generation depends on evaluating participants’ health status (health risk, vital signs, body measurement data) and contextual information. Each generated recommendation consists of a message and the corresponding timestamp (Figure 16). A bad habit (H) has a significant impact on healthy dietary practice. Activities are related to the context (C). Contextual data help recommend participants to plan for indoor/outdoor activities based on the following day’s external weather conditions. The data properties of “RecommendationMessages” for activities are “hasActivityMessages” and “hasContextualMessages,” whereas those for the diet are “hasDietaryMessages” and “hasHabitRelatedMessages.” The identified set of recommendation messages for test setup (ontology verification) is presented in Multimedia Appendix 3, and is prepared based on the positive psychology [79] and the persuasion [80] concept.

Description logic [35,81] is a formal knowledge representation of the ontology language that offers a good trade-off between expressivity, complexity, and efficiency in knowledge representation and reasoning about structured knowledge. To ensure that the paper is perfectly understood, we have the propositional variables with their linked recommendation messages. Now, we need a set of clauses such that some models will assign these variables to true and thus trigger the sending of a recommendation. The description logic SROIQ [82,83], which is logic providing a formal underpinning of OWL2, has been used as the formal logic to reason in this paper (Multimedia Appendix 4).

### Rule Creation for Querying, Recommendation Generation, and Ensuring Satisfiability

**Figure 17:**
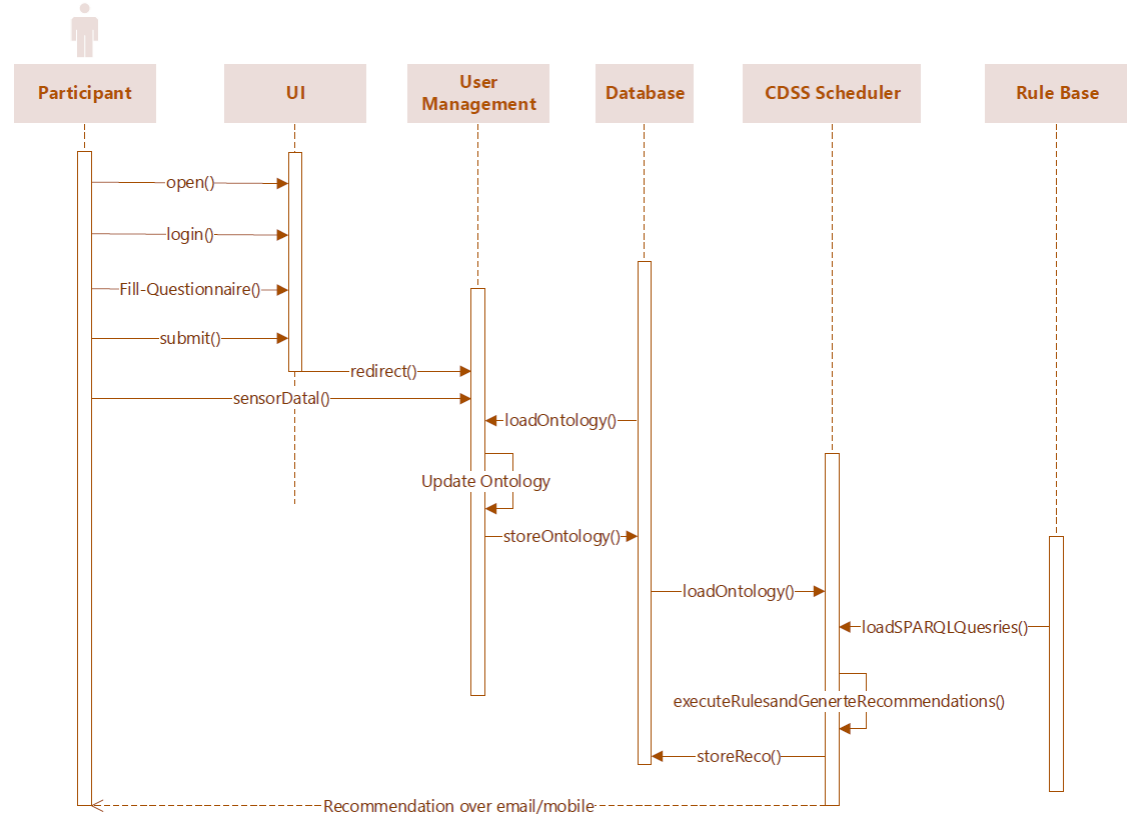
UML sequence diagram for recommendation generation and delivery.

A rule consists of a premise (antecedent) and a conclusion (premise). For every condition mentioned in Multimedia Appendix 3, DSS executes SPARQL queries daily to determine what type of recommendation message is to be delivered to each participant as depicted in the unified modeling language sequence diagram (Figure 17). The execution of every predefined semantic rule as specified in Multimedia Appendix 4 relies on the SPARQL query execution, and the rules are created following clinical guidelines, as stated in Multimedia Appendix 5 [62,84-92]. In this study, 20 semantic rules are subdivided into activity-level classification (8), habit-related classification (3), dietary classification (4), weather-level classification (1), obesity-level classification (3), and satisfiability (1) (please also see Multimedia Appendix 4). Moreover, except for the already-existing ontologies used, to ensure some consistency regarding what a participant is, what are the participant health records, etc., the concepts and the rules added are relatively easy to follow, and therefore they will be relatively easy to use.

The observable and measurable parameters associated with activities, habit, nutrition, and context (as described in Multimedia Appendix 4) for individual participants on a timestamp are obtained based on the execution of SPARQL queries by DSS on a daily scheduled interval as specified in Multimedia Appendix 6. The rules 17-19 in Multimedia Appendix 4 assign truth values to variables that ensure consistency with concepts already existing in the SNOMED CT ontology, where the body measurement is defined. We have confirmed with HermiT that for 4 specific cases the correct recommendation messages are triggered. However, one would need to ensure that there is not a combination of variables such that the whole formula is unsatisfiable (ie, no model can satisfy the procedure). One would also need to ensure that only 1 message can be triggered at a time. In this study, we have a formal guarantee that 2 “once-a-day” messages can neither be triggered simultaneously nor for every possible combination of variables, there is, every time, a model output by HermiT. If we put the different variables used in the first 19 rules (Multimedia Appendix 4) into propositional variables, we would have an exponential number of “possible participants.” One formal way to ensure a model’s existence is to negate all our rules and ensure the same. Then, the formula is indeed unsatisfiable. As 2 messages cannot be triggered at the same time, and to satisfy the same, we added a rule (rule 20) on the variables used in the recommendations started “once-a-day.” If rule 20 is false, then the whole set of rules (considered as a large conjunction) will be set to false. It will result in “no execution” of the proposition (see Multimedia Appendix 3) and will help us to debug our defined semantic rules (rules 1-19) as defined in Multimedia Appendix 4. If it is set to true, we have a formal guarantee that no 2 “once-a-day” messages can be triggered at the same time, no matter the truth values we put into our ABox.

## Results

**Figure 18:**
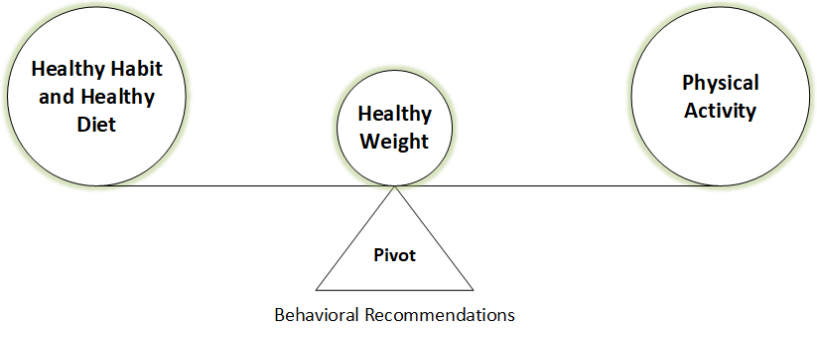
Behavioral recommendation generation (pivot) for the management of healthy lifestyle (a trade-off between physical activity, healthy habit, and healthy diet).

The test setup to verify the proposed eHealth ontology’s performance and reliability consisted of a DSS module (health risk prediction and recommendation generation for a healthy lifestyle), SPARQL, and rule base. As an outcome of ontology verification, we generated personalized and contextual recommendations (behavioral) following semantic rules to balance individual weight change with adopting healthy behavior to balance a trade-off between physical activity, healthy habit, and a healthy diet as depicted in Figure 18. We executed all the semantic rules as stated in Multimedia Appendix 4 in the form of SPARQL queries using the Jena ARQ engine on each participant’s simulated data as mentioned in Multimedia Appendix 2. We then determined what type of recommendation messages would be required to be delivered for each participant to manage his/her healthy lifestyle. These findings are detailed in Table 1.

**Table 1:** Recommendation generation for participants for Day-(n+1) [n>0].

Participant	Profile	SCTID	Healthy habit on Day-n	Healthy diet on Day-n	Physically active on Day-n	Recommendation(s) for Day-(n+1)
Individual_1	Normal weight	43664005	No	No	Yes	H-1, D-2, D-3, A-4, C-1
Individual_2	Normal weight	43664005	No	Yes	No	H-1, H-2, D-4, A-3, C-1
Individual_3	Overweight	162863004	No	No	No	H-2, D-1, D-2, D-3, A-2, A-5, C-1
Individual_4	Overweight	162863004	Yes	No	No	D-1, D-2, A-1, C-1

## Discussion

### Principal Findings

According to Table 1, “Individual_1” and” Individual_2” are healthy weight participants, and “Individual_3” and “Individual_4” are overweight participants as assessed based on their daily (“Day-n”) BMI (weight/height^2^) value. According to Figure 1, a healthy weight is a trade-off between healthy habits, healthy diet, and physical activity. On “Day-n” (n>0), “Individual_1” has been physically active, and this is the reason he has been encouraged to keep up the same activity level (A-4). By contrast, he has shown some addiction toward “snus,” sweet beverages, and fried/processed foods, which might grow negative behavior in the participant and increase his weight. Therefore, he has been recommended to reduce tobacco consumptions (H-1) and to refrain from discretionary food items (D-2 and D-3). The simulated data for “Individual_2” has demonstrated that she is inclined to a healthy diet (D-4), but growing some negative behavior with consumption of alcohol and tobacco (H-1, H-2). She is just one step behind to become physically active (A-3). Hence, she has been recommended to take a healthy dietary plan, refrain from tobacco and alcohol, and increase activity level to become active. “Individual_3” is neither physically active nor adhered to healthy habits or healthy dietary plans. He is addicted to alcohol, fried/processed foods, sweet beverages, sweet food/milk products. His consumed number of vegetables and fruits is not adequate for a healthy diet (<400 g). Therefore, he has been recommended to reduce alcohol consumption (H-1), to follow a healthy dietary habit (D-1, D-2, D-3), and to become more physically active (A-2) with adequate sleeping (A-5). The fabricated data for “Individual_4” has shown that she has an unhealthy diet plan, and she is mostly leading a sedentary lifestyle. Therefore, she has been recommended to stay away from discretionary food items (D-2), to incline on “core-foods” (D-1), and to increase activity level by one step (A-1). The analysis of contextual data reveals that the weather on “Day-(n+1)” is suitable for outdoor activities. The purpose of the individualized recommendation generation is to guide and encourage individual participants to keep up a healthy lifestyle by maintaining a balance between healthy habit, healthy diet, and physical activity. It encourages people with a normal weight to maintain their healthy weight, and those with obesity/overweight to reduce their weight.

**Figure 19:**
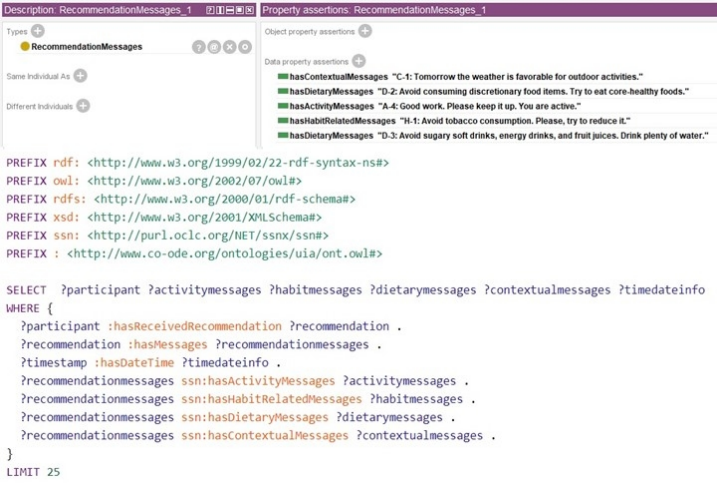
Sample SPARQL query for recommendation finding (e.g., “Individual_1”).

**Figure 20:**
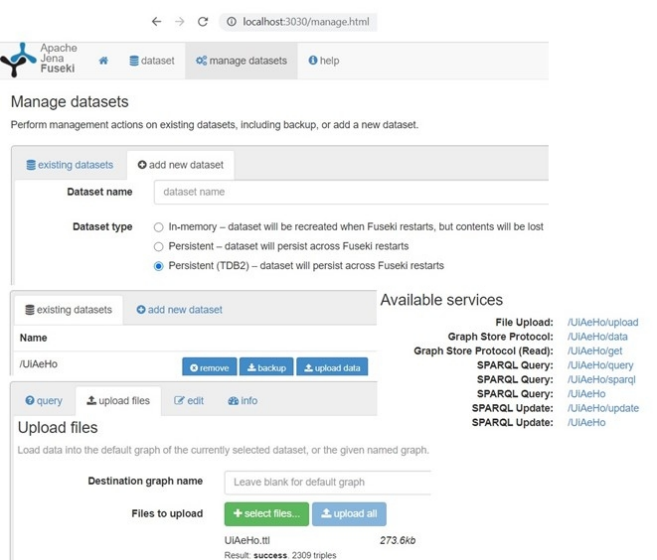
Integration of TDB with Jena Fuseki for ontology store in the “ttl” format and querying.

The rule-based decision support has generated personalized and contextual recommendations (Table 1) using SPARQL queries, as depicted in Figure 19, based on the proposed ontology without any “false-positive” case. The proposed ontology’s reasoning time has been measured as <30.0 seconds in Protégé with HermiT reasoner without reporting any inconsistencies. The reading time of the ontology after loading it in the Jena workspace was about 2.0-3.5 seconds with the “OWL_MEM_MICRO_RULE_INF” ontology specification in the “TTL” format (OWL full), “in-memory” storage, and “optimized rule-based reasoner with OWL rules.” Then, we queried ontology classes, ontologies, “predicate, subject, and object” of every statement using Jena in <1.5 seconds, <0.5 seconds, and <3.5 seconds, respectively. Each ontology model (complete RDF graph) is related to a document manager (default global document manager: “OntDocumentManager”) to assist with the processing and handling of ontology documents. All the classes in the ontology API that represent ontology values have “OntResource” as a common super-class with attributes (versionInfo, comment, label, seeAlso, isDefinedBy, sameAs, and differentFrom) and methods (add, set, list, get, has, and remove). We used the implementation of the RDF interface, provided by Jena, to store the modeled ontology and its instances persistently in the tuple database and load it back to process further. Jena Fuseki is tightly integrated with tuple database to provide a robust, transactional persistent storage layer (Figure 20).

In the future study, the recommendation process can be automated with the amalgamation of a hybrid DSS system (rule based and data driven) and AI algorithms. The scope of the proposed ontology can be enhanced with the integration of (1) real sensor activity devices; (2) mood assessment of participants; (3) collection of nutrition data on a detailed level through multiple questionnaires (daily, on every alternative day, and weekly); (4) semantic annotation of the recommended messages; (5) weekly suggestion generation after evaluating daily generated recommendations, and followed by a ranking of participants based on their weekly performances; (6) help-desk management for technical support; (7) assessment of baseline data; (8) trend analysis of health risks as a function of habit, diet, and activity with machine intelligence; and (9) automated interview management by trained health professionals (nurses).

### Conclusions

In health care, with the research advancement on the IoT domain, an increasing number of sensors, actuators, mobile, and web-based health monitoring devices are deployed into our daily life for remote health monitoring. It produces enormous personalized health and wellness observable and measurable data with hidden patterns. Data collected by multichannel sensors or devices demonstrate significant differences in data formats, types, and domains, which might lead to a problem in machine understandability. Therefore, a semantic representation of collected health and wellness data from heterogeneous sources is necessary, and the ontology serves the purpose. In this pilot study, we have proposed an eHealth ontology model in association with SSN and SNOMED CT, to support a semantic representation of collected observable and measurable data to manage a healthy lifestyle focusing on obesity as a case study. The ontology represents collected data with OWL-based web language in RDF triple-store format. The performance of the proposed ontology has been evaluated with the simulated data (eg, sensor, interview, and questionnaire) of 4 dummy participants. The proposed ontology’s structural and logical consistency has been evaluated with a Protégé reasoner (HermiT 1.4.3.x). The proposed ontology model has been used by a rule-based DSS to generate personalized and contextual recommendations with the execution of SPARQL queries against a preset rule base (with the help of Apache Jena library) to promote a healthy lifestyle for obesity management. In the future study, we will recruit real participants following inclusion and exclusion criteria and provide them real activity devices to replicate the whole scenario and evaluate the efficacy of the recommendation generation plan. The proposed ontology can be extended to annotate observable and measurable data for other related lifestyle diseases, such as diabetes type II, chronic obstructive pulmonary diseases, cardiovascular diseases, and mental health.

## Appendix

Multimedia Appendix 1 Proposed ontology model's OWL file with annotated participant data.

Multimedia Appendix 2 Simulated data for 4 participants.

Multimedia Appendix 3 Propositional variables with their linked recommendation messages.

Multimedia Appendix 4 Scoped recommendation conditions, and corresponding rules (rule-base) for test set-up.

Multimedia Appendix 5 Health parameters and corresponding clinical rules [70-78].

Multimedia Appendix 6 Prefixes and queries.

## Abbreviations

AI: artificial intelligence
API: application programming interface
BLE: Bluetooth low energy
BP: blood pressure
CDSS: clinical decision support system
DSS: decision support system
ICD-11: International Classification of Diseases (11th edition)
ICT: information and communications technology
KB: knowledge base
LOINC: Logical Observation Identifiers Names and Codes
NICE: National Institute for Health and Care Excellence
RDF: resource description framework
RDF: resource description framework
RDFS: RDF schema
SNOMED CT: Systematized Nomenclature of Medicine—Clinical Terms
SPARQL: Simple Protocol and RDF Query Language
SSN: semantic sensor network
SWRL: semantic web rule language
UMLS: Unified Medical Lexicon System
URI: unified resource identifier
WHO: World Health Organization
