# Supplementary material for: An Automatic Ontology-Based Approach to Support Logical Representation of Observable and Measurable Data for Healthy Lifestyle Management: Proof-of-Concept Study
Source: J Med Internet Res. 2021 Apr 9;23(4):e24656. doi: 10.2196/24656 (PMC8065560; doi:10.2196/24656)
Supplement: Multimedia Appendix 1 [file jmir_v23i4e24656_app1.docx]

@prefix : <http://purl.oclc.org/NET/ssnx/ssn> .

@prefix cc: <http://creativecommons.org/ns#> .

@prefix dc: <http://purl.org/dc/elements/1.1/> .

@prefix DUL: <http://www.loa-cnr.it/ontologies/DUL.owl#> .

@prefix dct: <http://purl.org/dc/terms/> .

@prefix owl: <http://www.w3.org/2002/07/owl#> .

@prefix rdf: <http://www.w3.org/1999/02/22-rdf-syntax-ns#> .

@prefix ssn: <http://purl.oclc.org/NET/ssnx/ssn#> .

@prefix xml: <http://www.w3.org/XML/1998/namespace> .

@prefix xsd: <http://www.w3.org/2001/XMLSchema#> .

@prefix obda: <https://w3id.org/obda/vocabulary#> .

@prefix rdfs: <http://www.w3.org/2000/01/rdf-schema#> .

@prefix skos: <http://www.w3.org/2004/02/skos/core#> .

@base <http://purl.oclc.org/NET/ssnx/ssn> .

<http://purl.oclc.org/NET/ssnx/ssn> rdf:type owl:Ontology ;

cc:license <http://www.w3.org/Consortium/Legal/2002/copyright-software-20021231.html> ;

dc:creator "W3C Semantic Sensor Network Incubator Group" ;

dc:identifier "http://purl.oclc.org/NET/ssnx/ssn" ;

dc:rights "Copyright 2009 - 2011 W3C." ;

dc:title "Semantic Sensor Network Ontology" ;

dct:created "2009-12-02" ;

dct:modified "2011-06-20" ;

rdfs:comment " Please report any errors to the Semantic Sensor Network Incubator Activity via the public W3C list public-xg-ssn@w3.org" ,

"This ontology describes sensors and observations, and related concepts. It does not describe domain concepts, time, locations, etc. these are intended to be included from other ontologies via OWL imports." ,

"This ontology is developed by the W3C Semantic Sensor Networks Incubator Group (SSN-XG). The concepts and structure of the ontology were discussed in the group's meetings and on the mailing list. For more information on the group's activities see: http://www.w3.org/2005/Incubator/ssn/" ;

rdfs:seeAlso "http://www.w3.org/2005/Incubator/ssn/" .

#################################################################

# Annotation properties

#################################################################

### http://creativecommons.org/ns#license

cc:license rdf:type owl:AnnotationProperty .

### http://purl.org/dc/elements/1.1/creator

dc:creator rdf:type owl:AnnotationProperty .

### http://purl.org/dc/elements/1.1/date

dc:date rdf:type owl:AnnotationProperty .

### http://purl.org/dc/elements/1.1/identifier

dc:identifier rdf:type owl:AnnotationProperty .

### http://purl.org/dc/elements/1.1/rights

dc:rights rdf:type owl:AnnotationProperty .

### http://purl.org/dc/elements/1.1/source

dc:source rdf:type owl:AnnotationProperty .

### http://purl.org/dc/elements/1.1/title

dc:title rdf:type owl:AnnotationProperty .

### http://purl.org/dc/terms/created

dct:created rdf:type owl:AnnotationProperty .

### http://purl.org/dc/terms/modified

dct:modified rdf:type owl:AnnotationProperty .

### http://www.w3.org/2000/01/rdf-schema#comment

rdfs:comment rdf:type owl:AnnotationProperty .

### http://www.w3.org/2000/01/rdf-schema#isDefinedBy

rdfs:isDefinedBy rdf:type owl:AnnotationProperty .

### http://www.w3.org/2000/01/rdf-schema#label

rdfs:label rdf:type owl:AnnotationProperty .

### http://www.w3.org/2000/01/rdf-schema#seeAlso

rdfs:seeAlso rdf:type owl:AnnotationProperty .

#################################################################

# Object Properties

#################################################################

### http://purl.oclc.org/NET/ssnx/ssn#attachedSystem

ssn:attachedSystem rdf:type owl:ObjectProperty ;

rdfs:subPropertyOf DUL:isLocationOf ;

owl:inverseOf ssn:onPlatform ;

rdfs:comment "Relation between a Platform and any Systems (e.g., Sensors) that are attached to the Platform." ;

rdfs:isDefinedBy "http://purl.oclc.org/NET/ssnx/ssn" ;

rdfs:label "attached system" ;

rdfs:seeAlso "http://www.w3.org/2005/Incubator/ssn/wiki/SSN_Deploy#PlatformSite" .

### http://purl.oclc.org/NET/ssnx/ssn#deployedOnPlatform

ssn:deployedOnPlatform rdf:type owl:ObjectProperty ;

rdfs:subPropertyOf DUL:hasParticipant ;

owl:inverseOf ssn:inDeployment ;

rdfs:comment "Relation between a deployment and the platform on which the system was deployed." ;

rdfs:isDefinedBy "http://purl.oclc.org/NET/ssnx/ssn" ;

rdfs:label "deployed on platform" ;

rdfs:seeAlso "http://www.w3.org/2005/Incubator/ssn/wiki/SSN_Deploy#Deployment" .

### http://purl.oclc.org/NET/ssnx/ssn#deployedSystem

ssn:deployedSystem rdf:type owl:ObjectProperty ;

rdfs:subPropertyOf DUL:hasParticipant ;

owl:inverseOf ssn:hasDeployment ;

rdfs:comment "Relation between a deployment and the deployed system." ;

rdfs:isDefinedBy "http://purl.oclc.org/NET/ssnx/ssn" ;

rdfs:label "deployed system" ;

rdfs:seeAlso "http://www.w3.org/2005/Incubator/ssn/wiki/SSN_Deploy#Deployment" .

### http://purl.oclc.org/NET/ssnx/ssn#deploymentProcessPart

ssn:deploymentProcessPart rdf:type owl:ObjectProperty ;

rdfs:subPropertyOf DUL:hasPart ;

rdfs:comment "Has part relation between a deployment process and its constituent processes." ;

rdfs:isDefinedBy "http://purl.oclc.org/NET/ssnx/ssn" ;

rdfs:label "deployment process part" ;

rdfs:seeAlso "http://www.w3.org/2005/Incubator/ssn/wiki/SSN_Deploy#Deployment" .

### http://purl.oclc.org/NET/ssnx/ssn#detects

ssn:detects rdf:type owl:ObjectProperty ;

rdfs:comment """A relation from a sensor to the Stimulus that the sensor can detect.

The Stimulus itself will be serving as a proxy for (see isProxyOf) some observable property.""" ;

rdfs:isDefinedBy "http://purl.oclc.org/NET/ssnx/ssn" ;

rdfs:label "detects" ;

rdfs:seeAlso "http://www.w3.org/2005/Incubator/ssn/wiki/SSN_Skeleton#Skeleton" .

### http://purl.oclc.org/NET/ssnx/ssn#endTime

ssn:endTime rdf:type owl:ObjectProperty ;

rdfs:subPropertyOf DUL:hasRegion ;

rdfs:isDefinedBy "http://purl.oclc.org/NET/ssnx/ssn" ;

rdfs:label "end time" ;

rdfs:seeAlso "http://www.w3.org/2005/Incubator/ssn/wiki/SSN_Base#Time" .

### http://purl.oclc.org/NET/ssnx/ssn#featureOfInterest

ssn:featureOfInterest rdf:type owl:ObjectProperty ;

rdfs:subPropertyOf DUL:isSettingFor ;

dc:source """skos:exactMatch 'featureOfInterest' [O&M - ISO/DIS 19156]

http://portal.opengeospatial.org/files/?artifact_id=41579""" ;

rdfs:comment "A relation between an observation and the entity whose quality was observed. For example, in an observation of the weight of a person, the feature of interest is the person and the quality is weight." ;

rdfs:isDefinedBy "http://purl.oclc.org/NET/ssnx/ssn" ;

rdfs:label "feature of interest" ;

rdfs:seeAlso "http://www.w3.org/2005/Incubator/ssn/wiki/SSN_Skeleton#Skeleton" .

### http://purl.oclc.org/NET/ssnx/ssn#forProperty

ssn:forProperty rdf:type owl:ObjectProperty ;

rdfs:comment "A relation between some aspect of a sensing entity and a property. For example, from a sensor to the properties it can observe, or from a deployment to the properties it was installed to observe. Also from a measurement capability to the property the capability is described for. (Used in conjunction with ofFeature)." ;

rdfs:isDefinedBy "http://purl.oclc.org/NET/ssnx/ssn" ;

rdfs:label "for property" ;

rdfs:seeAlso "http://www.w3.org/2005/Incubator/ssn/wiki/SSN_Skeleton#Skeleton" .

### http://purl.oclc.org/NET/ssnx/ssn#hasBeenHandledBy

ssn:hasBeenHandledBy rdf:type owl:ObjectProperty ;

owl:inverseOf ssn:manages .

### http://purl.oclc.org/NET/ssnx/ssn#hasClinicalFinding

ssn:hasClinicalFinding rdf:type owl:ObjectProperty ;

rdfs:domain <http://www.co-ode.org/ontologies/uia/ont.owl#ParticipantState> ;

rdfs:range <http://www.co-ode.org/ontologies/uia/ont.owl#ClinicalFinding> .

### http://purl.oclc.org/NET/ssnx/ssn#hasContextData

ssn:hasContextData rdf:type owl:ObjectProperty ;

rdfs:domain <http://www.co-ode.org/ontologies/uia/ont.owl#Recommendation> ;

rdfs:range <http://www.co-ode.org/ontologies/uia/ont.owl#ContextualData> .

### http://purl.oclc.org/NET/ssnx/ssn#hasDeployment

ssn:hasDeployment rdf:type owl:ObjectProperty ;

rdfs:subPropertyOf DUL:isParticipantIn ;

rdfs:comment "Relation between a System and a Deployment, recording that the System/Sensor was deployed in that Deployment." ;

rdfs:isDefinedBy "http://purl.oclc.org/NET/ssnx/ssn" ;

rdfs:label "has deployment" ;

rdfs:seeAlso "http://www.w3.org/2005/Incubator/ssn/wiki/SSN_Deploy#Deployment" .

### http://purl.oclc.org/NET/ssnx/ssn#hasInput

ssn:hasInput rdf:type owl:ObjectProperty ;

rdfs:isDefinedBy "http://purl.oclc.org/NET/ssnx/ssn" ;

rdfs:label "has input" ;

rdfs:seeAlso "http://www.w3.org/2005/Incubator/ssn/wiki/SSN_Model#Process" .

### http://purl.oclc.org/NET/ssnx/ssn#hasMeasurementCapability

ssn:hasMeasurementCapability rdf:type owl:ObjectProperty ;

rdfs:subPropertyOf ssn:hasProperty ;

rdfs:comment "Relation from a Sensor to a MeasurementCapability describing the measurement properties of the sensor." ;

rdfs:isDefinedBy "http://purl.oclc.org/NET/ssnx/ssn" ;

rdfs:label "has measurement capability" ;

rdfs:seeAlso "http://www.w3.org/2005/Incubator/ssn/wiki/SSN_Sensor#MeasuringCapability" .

### http://purl.oclc.org/NET/ssnx/ssn#hasMeasurementProperty

ssn:hasMeasurementProperty rdf:type owl:ObjectProperty ;

rdfs:subPropertyOf ssn:hasProperty ;

rdfs:comment "Relation from a MeasurementCapability to a MeasurementProperty. For example, to an accuracy (see notes at MeasurementCapability)." ;

rdfs:isDefinedBy "http://purl.oclc.org/NET/ssnx/ssn" ;

rdfs:label "has measurement property" ;

rdfs:seeAlso "http://www.w3.org/2005/Incubator/ssn/wiki/SSN_Sensor#MeasuringCapability" .

### http://purl.oclc.org/NET/ssnx/ssn#hasObservables

ssn:hasObservables rdf:type owl:ObjectProperty ;

rdfs:domain <http://www.co-ode.org/ontologies/uia/ont.owl#ParticipantHealthRecord> ;

rdfs:range <http://www.co-ode.org/ontologies/uia/ont.owl#ObservableEntity> .

### http://purl.oclc.org/NET/ssnx/ssn#hasOperatingProperty

ssn:hasOperatingProperty rdf:type owl:ObjectProperty ;

rdfs:subPropertyOf ssn:hasProperty ;

rdfs:comment "Relation from an OperatingRange to a Property. For example, to a battery lifetime." ;

rdfs:isDefinedBy "http://purl.oclc.org/NET/ssnx/ssn" ;

rdfs:label "has operating property" ;

rdfs:seeAlso "http://www.w3.org/2005/Incubator/ssn/wiki/SSN_Deploy#OperatingRestriction" .

### http://purl.oclc.org/NET/ssnx/ssn#hasOperatingRange

ssn:hasOperatingRange rdf:type owl:ObjectProperty ;

rdfs:subPropertyOf ssn:hasProperty ;

rdfs:comment "Relation from a System to an OperatingRange describing the normal operating environment of the System." ;

rdfs:isDefinedBy "http://purl.oclc.org/NET/ssnx/ssn" ;

rdfs:label "has operating range" ;

rdfs:seeAlso "http://www.w3.org/2005/Incubator/ssn/wiki/SSN_Deploy#OperatingRestriction" .

### http://purl.oclc.org/NET/ssnx/ssn#hasOutput

ssn:hasOutput rdf:type owl:ObjectProperty ;

rdfs:isDefinedBy "http://purl.oclc.org/NET/ssnx/ssn" ;

rdfs:label "has output" ;

rdfs:seeAlso "http://www.w3.org/2005/Incubator/ssn/wiki/SSN_Model#Process" .

### http://purl.oclc.org/NET/ssnx/ssn#hasProperty

ssn:hasProperty rdf:type owl:ObjectProperty ;

rdfs:subPropertyOf DUL:hasQuality ;

owl:inverseOf ssn:isPropertyOf ;

rdfs:comment "A relation between a FeatureOfInterest and a Property of that feature." ;

rdfs:isDefinedBy "http://purl.oclc.org/NET/ssnx/ssn" ;

rdfs:label "has property" ;

rdfs:seeAlso "http://www.w3.org/2005/Incubator/ssn/wiki/SSN_Skeleton#Skeleton" .

### http://purl.oclc.org/NET/ssnx/ssn#hasRegisteredComplaint

ssn:hasRegisteredComplaint rdf:type owl:ObjectProperty ;

rdfs:domain <http://www.co-ode.org/ontologies/uia/ont.owl#Participant> .

### http://purl.oclc.org/NET/ssnx/ssn#hasSubSystem

ssn:hasSubSystem rdf:type owl:ObjectProperty ;

rdfs:subPropertyOf DUL:hasPart ;

rdfs:comment "Haspart relation between a system and its parts." ;

rdfs:isDefinedBy "http://purl.oclc.org/NET/ssnx/ssn" ;

rdfs:label "has subsystem" ;

rdfs:seeAlso "http://www.w3.org/2005/Incubator/ssn/wiki/SSN_Model#System" .

### http://purl.oclc.org/NET/ssnx/ssn#hasSurvivalProperty

ssn:hasSurvivalProperty rdf:type owl:ObjectProperty ;

rdfs:subPropertyOf ssn:hasProperty ;

rdfs:comment "Relation from a SurvivalRange to a Property describing the survial range of a system. For example, to the temperature extreme that a system can withstand before being considered damaged." ;

rdfs:isDefinedBy "http://purl.oclc.org/NET/ssnx/ssn" ;

rdfs:label "has survival property" ;

rdfs:seeAlso "http://www.w3.org/2005/Incubator/ssn/wiki/SSN_Deploy#OperatingRestriction" .

### http://purl.oclc.org/NET/ssnx/ssn#hasSurvivalRange

ssn:hasSurvivalRange rdf:type owl:ObjectProperty ;

rdfs:subPropertyOf ssn:hasProperty ;

rdfs:comment "A Relation from a System to a SurvivalRange." ;

rdfs:isDefinedBy "http://purl.oclc.org/NET/ssnx/ssn" ;

rdfs:label "has survival range" ;

rdfs:seeAlso "http://www.w3.org/2005/Incubator/ssn/wiki/SSN_Deploy#OperatingRestriction" .

### http://purl.oclc.org/NET/ssnx/ssn#hasValue

ssn:hasValue rdf:type owl:ObjectProperty ;

rdfs:subPropertyOf DUL:hasRegion ;

rdfs:isDefinedBy "http://purl.oclc.org/NET/ssnx/ssn" ;

rdfs:label "has value" ;

rdfs:seeAlso "http://www.w3.org/2005/Incubator/ssn/wiki/SSN_Base#Data" .

### http://purl.oclc.org/NET/ssnx/ssn#hasVitalSignFinding

ssn:hasVitalSignFinding rdf:type owl:ObjectProperty ;

rdfs:domain <http://www.co-ode.org/ontologies/uia/ont.owl#ParticipantState> ;

rdfs:range <http://www.co-ode.org/ontologies/uia/ont.owl#Vital_sign_finding> .

### http://purl.oclc.org/NET/ssnx/ssn#implementedBy

ssn:implementedBy rdf:type owl:ObjectProperty ;

rdfs:subPropertyOf DUL:describes ;

owl:inverseOf ssn:implements ;

rdfs:comment "A relation between the description of an algorithm, procedure or method and an entity that implements that method in some executable way. For example, between a scientific measuring method and a sensor the senses via that method." ;

rdfs:isDefinedBy "http://purl.oclc.org/NET/ssnx/ssn" ;

rdfs:label "implemented by" ;

rdfs:seeAlso "http://www.w3.org/2005/Incubator/ssn/wiki/SSN_Skeleton#Skeleton" .

### http://purl.oclc.org/NET/ssnx/ssn#implements

ssn:implements rdf:type owl:ObjectProperty ;

rdfs:subPropertyOf DUL:isDescribedBy ;

rdfs:comment "A relation between an entity that implements a method in some executable way and the description of an algorithm, procedure or method. For example, between a Sensor and the scientific measuring method that the Sensor uses to observe a Property." ;

rdfs:isDefinedBy "http://purl.oclc.org/NET/ssnx/ssn" ;

rdfs:label "implements" ;

rdfs:seeAlso "http://www.w3.org/2005/Incubator/ssn/wiki/SSN_Skeleton#Skeleton" .

### http://purl.oclc.org/NET/ssnx/ssn#inCondition

ssn:inCondition rdf:type owl:ObjectProperty ;

rdfs:comment "Describes the prevailing environmental conditions for MeasurementCapabilites, OperatingConditions and SurvivalRanges. Used for example to say that a sensor has a particular accuracy in particular conditions. (see also MeasurementCapability)" ;

rdfs:isDefinedBy "http://purl.oclc.org/NET/ssnx/ssn" ;

rdfs:label "in condition" ;

rdfs:seeAlso "http://www.w3.org/2005/Incubator/ssn/wiki/SSN_Base#ConstraintBlock" .

### http://purl.oclc.org/NET/ssnx/ssn#inDeployment

ssn:inDeployment rdf:type owl:ObjectProperty ;

rdfs:subPropertyOf DUL:isParticipantIn ;

rdfs:comment "Relation between a Platform and a Deployment, recording that the object was used as a platform for a system/sensor for a particular deployment: as in this PhysicalObject is acting as a Platform inDeployment Deployment." ;

rdfs:isDefinedBy "http://purl.oclc.org/NET/ssnx/ssn" ;

rdfs:label "in deployment" ;

rdfs:seeAlso "http://www.w3.org/2005/Incubator/ssn/wiki/SSN_Deploy#Deployment" .

### http://purl.oclc.org/NET/ssnx/ssn#isFoundBy

ssn:isFoundBy rdf:type owl:ObjectProperty .

### http://purl.oclc.org/NET/ssnx/ssn#isProducedBy

ssn:isProducedBy rdf:type owl:ObjectProperty ;

owl:inverseOf <http://www.co-ode.org/ontologies/uia/ont.owl#hasOutput> ;

rdfs:comment "Relation between a producer and a produced entity: for example, between a sensor and the produced output." ;

rdfs:isDefinedBy "http://purl.oclc.org/NET/ssnx/ssn" ;

rdfs:label "is produced by" ;

rdfs:seeAlso "http://www.w3.org/2005/Incubator/ssn/wiki/SSN_Model#Process" .

### http://purl.oclc.org/NET/ssnx/ssn#isPropertyOf

ssn:isPropertyOf rdf:type owl:ObjectProperty ;

rdfs:subPropertyOf DUL:isQualityOf ;

rdfs:comment "Relation between a FeatureOfInterest and a Property (a Quality observable by a sensor) of that feature." ;

rdfs:isDefinedBy "http://purl.oclc.org/NET/ssnx/ssn" ;

rdfs:label "is property of" ;

rdfs:seeAlso "http://www.w3.org/2005/Incubator/ssn/wiki/SSN_Skeleton#Skeleton" .

### http://purl.oclc.org/NET/ssnx/ssn#isProxyFor

ssn:isProxyFor rdf:type owl:ObjectProperty ;

rdfs:comment "A relation from a Stimulus to the Property that the Stimulus is serving as a proxy for. For example, the expansion of the quicksilver is a stimulus that serves as a proxy for temperature, or an increase or decrease in the spinning of cups on a wind sensor is serving as a proxy for wind speed." ;

rdfs:isDefinedBy "http://purl.oclc.org/NET/ssnx/ssn" ;

rdfs:label "isProxyFor" ;

rdfs:seeAlso "http://www.w3.org/2005/Incubator/ssn/wiki/SSN_Skeleton#Skeleton" .

### http://purl.oclc.org/NET/ssnx/ssn#madeObservation

ssn:madeObservation rdf:type owl:ObjectProperty ;

rdfs:subPropertyOf DUL:isObjectIncludedIn ;

owl:inverseOf ssn:observedBy ;

rdfs:comment "Relation between a Sensor and Observations it has made." ;

rdfs:isDefinedBy "http://purl.oclc.org/NET/ssnx/ssn" ;

rdfs:label "made observation" ;

rdfs:seeAlso "http://www.w3.org/2005/Incubator/ssn/wiki/SSN_Observation#Observation" .

### http://purl.oclc.org/NET/ssnx/ssn#manages

ssn:manages rdf:type owl:ObjectProperty .

### http://purl.oclc.org/NET/ssnx/ssn#observationResult

ssn:observationResult rdf:type owl:ObjectProperty ;

rdfs:subPropertyOf DUL:isSettingFor ;

dc:source """skos:closeMatch 'result' [O&M - ISO/DIS 19156]

http://portal.opengeospatial.org/files/?artifact_id=41579""" ;

rdfs:comment "Relation linking an Observation (i.e., a description of the context, the Situation, in which the observatioin was made) and a Result, which contains a value representing the value associated with the observed Property." ;

rdfs:isDefinedBy "http://purl.oclc.org/NET/ssnx/ssn" ;

rdfs:label "observation result" ;

rdfs:seeAlso "http://www.w3.org/2005/Incubator/ssn/wiki/SSN_Skeleton#Skeleton" .

### http://purl.oclc.org/NET/ssnx/ssn#observationResultTime

ssn:observationResultTime rdf:type owl:ObjectProperty ;

rdfs:subPropertyOf DUL:hasRegion ;

dc:source "http://www.opengeospatial.org/standards/om" ;

rdfs:comment "The result time is the time when the procedure associated with the observation act was applied." ,

"The result time shall describe the time when the result became available, typically when the procedure associated with the observation was completed For some observations this is identical to the phenomenonTime. However, there are important cases where they differ.[O&M]" ;

rdfs:isDefinedBy "http://purl.oclc.org/NET/ssnx/ssn" ;

rdfs:label "observation result time" ;

rdfs:seeAlso "http://www.w3.org/2005/Incubator/ssn/wiki/SSN_Observation#Observation" .

### http://purl.oclc.org/NET/ssnx/ssn#observationSamplingTime

ssn:observationSamplingTime rdf:type owl:ObjectProperty ;

rdfs:subPropertyOf DUL:hasRegion ;

dc:source "http://www.opengeospatial.org/standards/om" ;

rdfs:comment "Rebadged as phenomenon time in [O&M]. The phenomenon time shall describe the time that the result applies to the property of the feature-of-interest. This is often the time of interaction by a sampling procedure or observation procedure with a real-world feature." ,

"The sampling time is the time that the result applies to the feature-of-interest. This is the time usually required for geospatial analysis of the result." ;

rdfs:isDefinedBy "http://purl.oclc.org/NET/ssnx/ssn" ;

rdfs:label "observation sampling time" ;

rdfs:seeAlso "http://www.w3.org/2005/Incubator/ssn/wiki/SSN_Observation#Observation" .

### http://purl.oclc.org/NET/ssnx/ssn#observedBy

ssn:observedBy rdf:type owl:ObjectProperty ;

rdfs:subPropertyOf DUL:includesObject ;

rdfs:isDefinedBy "http://purl.oclc.org/NET/ssnx/ssn" ;

rdfs:seeAlso "http://www.w3.org/2005/Incubator/ssn/wiki/SSN_Skeleton#Skeleton" .

### http://purl.oclc.org/NET/ssnx/ssn#observedProperty

ssn:observedProperty rdf:type owl:ObjectProperty ;

rdfs:subPropertyOf DUL:isSettingFor ;

dc:source """skos:exactMatch 'observedProperty' [O&M - ISO/DIS 19156]

http://portal.opengeospatial.org/files/?artifact_id=41579""" ;

rdfs:comment "Relation linking an Observation to the Property that was observed. The observedProperty should be a Property (hasProperty) of the FeatureOfInterest (linked by featureOfInterest) of this observation." ;

rdfs:isDefinedBy "http://purl.oclc.org/NET/ssnx/ssn" ;

rdfs:label "observed property" ;

rdfs:seeAlso "http://www.w3.org/2005/Incubator/ssn/wiki/SSN_Skeleton#Skeleton" .

### http://purl.oclc.org/NET/ssnx/ssn#observes

ssn:observes rdf:type owl:ObjectProperty ;

owl:propertyChainAxiom ( ssn:hasMeasurementCapability

ssn:forProperty

) ,

( ssn:madeObservation

ssn:observedProperty

) ;

rdfs:comment """Relation between a Sensor and a Property that the sensor can observe.

Note that, given the DUL modelling of Qualities, a sensor defined with 'observes only Windspeed' technically links the sensor to particular instances of Windspeed, not to the concept itself - OWL can't express concept-concept relations, only individual-individual. The property composition ensures that if an observation is made of a particular quality then one can infer that the sensor observes that quality.""" ;

rdfs:isDefinedBy "http://purl.oclc.org/NET/ssnx/ssn" ;

rdfs:label "observes" ;

rdfs:seeAlso "http://www.w3.org/2005/Incubator/ssn/wiki/SSN_Sensor#Measuring" .

### http://purl.oclc.org/NET/ssnx/ssn#ofFeature

ssn:ofFeature rdf:type owl:ObjectProperty ;

rdfs:comment "A relation between some aspect of a sensing entity and a feature. For example, from a sensor to the features it can observe properties of, or from a deployment to the features it was installed to observe. Also from a measurement capability to the feature the capability is described for. (Used in conjunction with forProperty)." ;

rdfs:isDefinedBy "http://purl.oclc.org/NET/ssnx/ssn" ;

rdfs:label "of feature" ;

rdfs:seeAlso "http://www.w3.org/2005/Incubator/ssn/wiki/SSN_Skeleton#Skeleton" .

### http://purl.oclc.org/NET/ssnx/ssn#onPlatform

ssn:onPlatform rdf:type owl:ObjectProperty ;

rdfs:subPropertyOf DUL:hasLocation ;

rdfs:comment "Relation between a System (e.g., a Sensor) and a Platform. The relation locates the sensor relative to other described entities entities: i.e., the Sensor s1's location is Platform p1. More precise locations for sensors in space (relative to other entities, where attached to another entity, or in 3D space) are made using DOLCE's Regions (SpaceRegion)." ;

rdfs:isDefinedBy "http://purl.oclc.org/NET/ssnx/ssn" ;

rdfs:label "on platform" ;

rdfs:seeAlso "http://www.w3.org/2005/Incubator/ssn/wiki/SSN_Deploy#PlatformSite" .

### http://purl.oclc.org/NET/ssnx/ssn#qualityOfObservation

ssn:qualityOfObservation rdf:type owl:ObjectProperty ;

rdfs:subPropertyOf ssn:hasProperty ;

dc:source """skos:exactMatch 'resultQuality' [O&M - ISO/DIS 19156]

http://portal.opengeospatial.org/files/?artifact_id=41579""" ;

rdfs:comment "Relation linking an Observation to the adjudged quality of the result. This is of course complimentary to the MeasurementCapability information recorded for the Sensor that made the Observation." ;

rdfs:isDefinedBy "http://purl.oclc.org/NET/ssnx/ssn" ;

rdfs:label "quality of observation" ;

rdfs:seeAlso "http://www.w3.org/2005/Incubator/ssn/wiki/SSN_Observation#Observation" .

### http://purl.oclc.org/NET/ssnx/ssn#sensingMethodUsed

ssn:sensingMethodUsed rdf:type owl:ObjectProperty ;

rdfs:subPropertyOf DUL:satisfies ;

dc:source "http://www.bipm.org/en/committees/jc/jcgm/wg2.html" ;

rdfs:comment "A (measurement) procedure is a detailed description of a measurement according to one or more measurement principles and to a given measurement method, based on a measurement model and including any calculation to obtain a measurement result [VIM 2.6]" ;

rdfs:isDefinedBy "http://purl.oclc.org/NET/ssnx/ssn" ;

rdfs:label "sensing method used" ;

rdfs:seeAlso "http://www.w3.org/2005/Incubator/ssn/wiki/SSN_Skeleton#Skeleton" .

### http://purl.oclc.org/NET/ssnx/ssn#startTime

ssn:startTime rdf:type owl:ObjectProperty ;

rdfs:subPropertyOf DUL:hasRegion ;

rdfs:isDefinedBy "http://purl.oclc.org/NET/ssnx/ssn" ;

rdfs:label "start time" ;

rdfs:seeAlso "http://www.w3.org/2005/Incubator/ssn/wiki/SSN_Base#Time" .

### http://www.co-ode.org/ontologies/uia/ont.owl#hasAppointmentTime

<http://www.co-ode.org/ontologies/uia/ont.owl#hasAppointmentTime> rdf:type owl:ObjectProperty ;

rdfs:domain <http://www.co-ode.org/ontologies/uia/ont.owl#ParticipantStatus> ;

rdfs:range <http://www.co-ode.org/ontologies/uia/ont.owl#TemporalEntity> .

### http://www.co-ode.org/ontologies/uia/ont.owl#hasBPData

<http://www.co-ode.org/ontologies/uia/ont.owl#hasBPData> rdf:type owl:ObjectProperty ;

rdfs:domain <http://www.co-ode.org/ontologies/uia/ont.owl#Blood_pressure> ;

rdfs:range <http://www.co-ode.org/ontologies/uia/ont.owl#BaselineData> .

### http://www.co-ode.org/ontologies/uia/ont.owl#hasBeenCollectedBy

<http://www.co-ode.org/ontologies/uia/ont.owl#hasBeenCollectedBy> rdf:type owl:ObjectProperty ;

rdfs:domain <http://www.co-ode.org/ontologies/uia/ont.owl#InterviewedPersonalData> ,

<http://www.co-ode.org/ontologies/uia/ont.owl#ParticipantHealthRecord> ;

rdfs:range DUL:Method .

### http://www.co-ode.org/ontologies/uia/ont.owl#hasBeenSetBy

<http://www.co-ode.org/ontologies/uia/ont.owl#hasBeenSetBy> rdf:type owl:ObjectProperty ;

rdfs:range <http://www.co-ode.org/ontologies/uia/ont.owl#Nurse> .

### http://www.co-ode.org/ontologies/uia/ont.owl#hasConductedBy

<http://www.co-ode.org/ontologies/uia/ont.owl#hasConductedBy> rdf:type owl:ObjectProperty ;

rdfs:domain <http://www.co-ode.org/ontologies/uia/ont.owl#Interview> ;

rdfs:range <http://www.co-ode.org/ontologies/uia/ont.owl#Nurse> .

### http://www.co-ode.org/ontologies/uia/ont.owl#hasHealthRecord

<http://www.co-ode.org/ontologies/uia/ont.owl#hasHealthRecord> rdf:type owl:ObjectProperty ;

rdfs:domain <http://www.co-ode.org/ontologies/uia/ont.owl#Participant> ;

rdfs:range <http://www.co-ode.org/ontologies/uia/ont.owl#ParticipantHealthRecord> .

### http://www.co-ode.org/ontologies/uia/ont.owl#hasHeartRate

<http://www.co-ode.org/ontologies/uia/ont.owl#hasHeartRate> rdf:type owl:ObjectProperty ;

rdfs:domain <http://www.co-ode.org/ontologies/uia/ont.owl#Pulse_rate_finding> ;

rdfs:range <http://www.co-ode.org/ontologies/uia/ont.owl#Pulse,function> .

### http://www.co-ode.org/ontologies/uia/ont.owl#hasInterviewPersonalData

<http://www.co-ode.org/ontologies/uia/ont.owl#hasInterviewPersonalData> rdf:type owl:ObjectProperty ;

rdfs:domain <http://www.co-ode.org/ontologies/uia/ont.owl#Participant> ;

rdfs:range <http://www.co-ode.org/ontologies/uia/ont.owl#InterviewedPersonalData> .

### http://www.co-ode.org/ontologies/uia/ont.owl#hasMessages

<http://www.co-ode.org/ontologies/uia/ont.owl#hasMessages> rdf:type owl:ObjectProperty ;

rdfs:domain <http://www.co-ode.org/ontologies/uia/ont.owl#Recommendation> ;

rdfs:range <http://www.co-ode.org/ontologies/uia/ont.owl#RecommendationMessages> .

### http://www.co-ode.org/ontologies/uia/ont.owl#hasOfficeAddress

<http://www.co-ode.org/ontologies/uia/ont.owl#hasOfficeAddress> rdf:type owl:ObjectProperty ;

rdfs:domain <http://www.co-ode.org/ontologies/uia/ont.owl#Admin> ,

<http://www.co-ode.org/ontologies/uia/ont.owl#Nurse> ,

<http://www.co-ode.org/ontologies/uia/ont.owl#Researcher> ;

rdfs:range <http://www.co-ode.org/ontologies/uia/ont.owl#OfficeAddress> .

### http://www.co-ode.org/ontologies/uia/ont.owl#hasOutput

<http://www.co-ode.org/ontologies/uia/ont.owl#hasOutput> rdf:type owl:ObjectProperty ;

rdfs:domain ssn:Sensor ;

rdfs:range ssn:SensorOutput .

### http://www.co-ode.org/ontologies/uia/ont.owl#hasParticipantState

<http://www.co-ode.org/ontologies/uia/ont.owl#hasParticipantState> rdf:type owl:ObjectProperty ;

rdfs:domain <http://www.co-ode.org/ontologies/uia/ont.owl#ParticipantStatus> ;

rdfs:range <http://www.co-ode.org/ontologies/uia/ont.owl#ParticipantState> .

### http://www.co-ode.org/ontologies/uia/ont.owl#hasParticipantStatus

<http://www.co-ode.org/ontologies/uia/ont.owl#hasParticipantStatus> rdf:type owl:ObjectProperty ;

rdfs:domain <http://www.co-ode.org/ontologies/uia/ont.owl#Recommendation> ;

rdfs:range <http://www.co-ode.org/ontologies/uia/ont.owl#ParticipantStatus> .

### http://www.co-ode.org/ontologies/uia/ont.owl#hasPersonalData

<http://www.co-ode.org/ontologies/uia/ont.owl#hasPersonalData> rdf:type owl:ObjectProperty ;

rdfs:domain <http://www.co-ode.org/ontologies/uia/ont.owl#Admin> ,

<http://www.co-ode.org/ontologies/uia/ont.owl#Nurse> ,

<http://www.co-ode.org/ontologies/uia/ont.owl#Researcher> ;

rdfs:range <http://www.co-ode.org/ontologies/uia/ont.owl#PersonalData> .

### http://www.co-ode.org/ontologies/uia/ont.owl#hasReceivedRecommendation

<http://www.co-ode.org/ontologies/uia/ont.owl#hasReceivedRecommendation> rdf:type owl:ObjectProperty ;

rdfs:domain <http://www.co-ode.org/ontologies/uia/ont.owl#Participant> ;

rdfs:range <http://www.co-ode.org/ontologies/uia/ont.owl#Recommendation> .

### http://www.co-ode.org/ontologies/uia/ont.owl#hasSimulatedInputData

<http://www.co-ode.org/ontologies/uia/ont.owl#hasSimulatedInputData> rdf:type owl:ObjectProperty ;

rdfs:domain <http://www.co-ode.org/ontologies/uia/ont.owl#SimulatedInputDataSequence> ;

rdfs:range <http://www.co-ode.org/ontologies/uia/ont.owl#SimulatedInputData> .

### http://www.co-ode.org/ontologies/uia/ont.owl#hasStatus

<http://www.co-ode.org/ontologies/uia/ont.owl#hasStatus> rdf:type owl:ObjectProperty ;

rdfs:domain <http://www.co-ode.org/ontologies/uia/ont.owl#Participant> ;

rdfs:range <http://www.co-ode.org/ontologies/uia/ont.owl#ParticipantStatus> .

### http://www.co-ode.org/ontologies/uia/ont.owl#hasTimeStamp

<http://www.co-ode.org/ontologies/uia/ont.owl#hasTimeStamp> rdf:type owl:ObjectProperty ;

rdfs:domain <http://www.co-ode.org/ontologies/uia/ont.owl#Interview> ,

<http://www.co-ode.org/ontologies/uia/ont.owl#Questionnaire> ;

rdfs:range <http://www.co-ode.org/ontologies/uia/ont.owl#TemporalEntity> .

### http://www.co-ode.org/ontologies/uia/ont.owl#hasUsedHealthRecord

<http://www.co-ode.org/ontologies/uia/ont.owl#hasUsedHealthRecord> rdf:type owl:ObjectProperty ;

rdfs:subPropertyOf owl:topObjectProperty ;

rdfs:domain <http://www.co-ode.org/ontologies/uia/ont.owl#Recommendation> ;

rdfs:range <http://www.co-ode.org/ontologies/uia/ont.owl#ParticipantHealthRecord> .

### http://www.loa-cnr.it/ontologies/DUL.owl#describes

DUL:describes rdf:type owl:ObjectProperty .

### http://www.loa-cnr.it/ontologies/DUL.owl#hasLocation

DUL:hasLocation rdf:type owl:ObjectProperty .

### http://www.loa-cnr.it/ontologies/DUL.owl#hasPart

DUL:hasPart rdf:type owl:ObjectProperty .

### http://www.loa-cnr.it/ontologies/DUL.owl#hasParticipant

DUL:hasParticipant rdf:type owl:ObjectProperty .

### http://www.loa-cnr.it/ontologies/DUL.owl#hasQuality

DUL:hasQuality rdf:type owl:ObjectProperty .

### http://www.loa-cnr.it/ontologies/DUL.owl#hasRegion

DUL:hasRegion rdf:type owl:ObjectProperty .

### http://www.loa-cnr.it/ontologies/DUL.owl#includesEvent

DUL:includesEvent rdf:type owl:ObjectProperty .

### http://www.loa-cnr.it/ontologies/DUL.owl#includesObject

DUL:includesObject rdf:type owl:ObjectProperty .

### http://www.loa-cnr.it/ontologies/DUL.owl#isDescribedBy

DUL:isDescribedBy rdf:type owl:ObjectProperty .

### http://www.loa-cnr.it/ontologies/DUL.owl#isLocationOf

DUL:isLocationOf rdf:type owl:ObjectProperty .

### http://www.loa-cnr.it/ontologies/DUL.owl#isObjectIncludedIn

DUL:isObjectIncludedIn rdf:type owl:ObjectProperty .

### http://www.loa-cnr.it/ontologies/DUL.owl#isParticipantIn

DUL:isParticipantIn rdf:type owl:ObjectProperty .

### http://www.loa-cnr.it/ontologies/DUL.owl#isQualityOf

DUL:isQualityOf rdf:type owl:ObjectProperty .

### http://www.loa-cnr.it/ontologies/DUL.owl#isRegionFor

DUL:isRegionFor rdf:type owl:ObjectProperty .

### http://www.loa-cnr.it/ontologies/DUL.owl#isSettingFor

DUL:isSettingFor rdf:type owl:ObjectProperty .

### http://www.loa-cnr.it/ontologies/DUL.owl#satisfies

DUL:satisfies rdf:type owl:ObjectProperty .

#################################################################

# Data properties

#################################################################

### http://purl.oclc.org/NET/ssnx/ssn#hasActivityMessages

ssn:hasActivityMessages rdf:type owl:DatatypeProperty .

### http://purl.oclc.org/NET/ssnx/ssn#hasAlcoholQuantity

ssn:hasAlcoholQuantity rdf:type owl:DatatypeProperty .

### http://purl.oclc.org/NET/ssnx/ssn#hasConsumedAlcohol

ssn:hasConsumedAlcohol rdf:type owl:DatatypeProperty .

### http://purl.oclc.org/NET/ssnx/ssn#hasContextualMessages

ssn:hasContextualMessages rdf:type owl:DatatypeProperty .

### http://purl.oclc.org/NET/ssnx/ssn#hasDietaryMessages

ssn:hasDietaryMessages rdf:type owl:DatatypeProperty .

### http://purl.oclc.org/NET/ssnx/ssn#hasFastingBloodGlucose

ssn:hasFastingBloodGlucose rdf:type owl:DatatypeProperty .

### http://purl.oclc.org/NET/ssnx/ssn#hasFriedorProcessedFood

ssn:hasFriedorProcessedFood rdf:type owl:DatatypeProperty .

### http://purl.oclc.org/NET/ssnx/ssn#hasFriedorProcessedFoodFrequency

ssn:hasFriedorProcessedFoodFrequency rdf:type owl:DatatypeProperty .

### http://purl.oclc.org/NET/ssnx/ssn#hasFruitAmount

ssn:hasFruitAmount rdf:type owl:DatatypeProperty .

### http://purl.oclc.org/NET/ssnx/ssn#hasHabitRelatedMessages

ssn:hasHabitRelatedMessages rdf:type owl:DatatypeProperty .

### http://purl.oclc.org/NET/ssnx/ssn#hasInitialBMI

ssn:hasInitialBMI rdf:type owl:DatatypeProperty .

### http://purl.oclc.org/NET/ssnx/ssn#hasInitialWeight

ssn:hasInitialWeight rdf:type owl:DatatypeProperty .

### http://purl.oclc.org/NET/ssnx/ssn#hasObservationTime

ssn:hasObservationTime rdf:type owl:DatatypeProperty ;

rdfs:subPropertyOf owl:topDataProperty ;

rdfs:domain <http://www.co-ode.org/ontologies/uia/ont.owl#TemporalEntity> ;

rdfs:range xsd:dateTimeStamp .

### http://purl.oclc.org/NET/ssnx/ssn#hasSmoked

ssn:hasSmoked rdf:type owl:DatatypeProperty .

### http://purl.oclc.org/NET/ssnx/ssn#hasSnusQuantity

ssn:hasSnusQuantity rdf:type owl:DatatypeProperty .

### http://purl.oclc.org/NET/ssnx/ssn#hasSweetBakeries

ssn:hasSweetBakeries rdf:type owl:DatatypeProperty .

### http://purl.oclc.org/NET/ssnx/ssn#hasSweetBakeriesFrequency

ssn:hasSweetBakeriesFrequency rdf:type owl:DatatypeProperty .

### http://purl.oclc.org/NET/ssnx/ssn#hasSweetBeverages

ssn:hasSweetBeverages rdf:type owl:DatatypeProperty .

### http://purl.oclc.org/NET/ssnx/ssn#hasSweetBeveragesAmount

ssn:hasSweetBeveragesAmount rdf:type owl:DatatypeProperty .

### http://purl.oclc.org/NET/ssnx/ssn#hasSweetFoodorMilkProduct

ssn:hasSweetFoodorMilkProduct rdf:type owl:DatatypeProperty .

### http://purl.oclc.org/NET/ssnx/ssn#hasSweetFoodorMilkProductFrequency

ssn:hasSweetFoodorMilkProductFrequency rdf:type owl:DatatypeProperty .

### http://purl.oclc.org/NET/ssnx/ssn#hasTakenSnus

ssn:hasTakenSnus rdf:type owl:DatatypeProperty .

### http://purl.oclc.org/NET/ssnx/ssn#hasTobaccoCount

ssn:hasTobaccoCount rdf:type owl:DatatypeProperty .

### http://purl.oclc.org/NET/ssnx/ssn#hasUserId

ssn:hasUserId rdf:type owl:DatatypeProperty .

### http://purl.oclc.org/NET/ssnx/ssn#hasVegetableAmount

ssn:hasVegetableAmount rdf:type owl:DatatypeProperty .

### http://purl.oclc.org/NET/ssnx/ssn#hasWaistHipRatio

ssn:hasWaistHipRatio rdf:type owl:DatatypeProperty .

### http://www.co-ode.org/ontologies/uia/ont.owl#hadLastAppointment

<http://www.co-ode.org/ontologies/uia/ont.owl#hadLastAppointment> rdf:type owl:DatatypeProperty ;

rdfs:domain <http://www.co-ode.org/ontologies/uia/ont.owl#TemporalEntity> ;

rdfs:range xsd:dateTimeStamp .

### http://www.co-ode.org/ontologies/uia/ont.owl#hasAccuracy

<http://www.co-ode.org/ontologies/uia/ont.owl#hasAccuracy> rdf:type owl:DatatypeProperty ;

rdfs:domain ssn:Accuracy ;

rdfs:range xsd:string .

### http://www.co-ode.org/ontologies/uia/ont.owl#hasActivityBouts

<http://www.co-ode.org/ontologies/uia/ont.owl#hasActivityBouts> rdf:type owl:DatatypeProperty ;

rdfs:domain <http://www.co-ode.org/ontologies/uia/ont.owl#ActivityDataValue> ;

rdfs:range xsd:integer .

### http://www.co-ode.org/ontologies/uia/ont.owl#hasAge

<http://www.co-ode.org/ontologies/uia/ont.owl#hasAge> rdf:type owl:DatatypeProperty ;

rdfs:domain <http://www.co-ode.org/ontologies/uia/ont.owl#InterviewedPersonalData> ;

rdfs:range xsd:integer .

### http://www.co-ode.org/ontologies/uia/ont.owl#hasCondition

<http://www.co-ode.org/ontologies/uia/ont.owl#hasCondition> rdf:type owl:DatatypeProperty ;

rdfs:domain ssn:Condition ;

rdfs:range xsd:string .

### http://www.co-ode.org/ontologies/uia/ont.owl#hasCurrentBMIValue

<http://www.co-ode.org/ontologies/uia/ont.owl#hasCurrentBMIValue> rdf:type owl:DatatypeProperty .

### http://www.co-ode.org/ontologies/uia/ont.owl#hasCurrentBloodSugarLevel

<http://www.co-ode.org/ontologies/uia/ont.owl#hasCurrentBloodSugarLevel> rdf:type owl:DatatypeProperty .

### http://www.co-ode.org/ontologies/uia/ont.owl#hasCurrentDiastolicBP

<http://www.co-ode.org/ontologies/uia/ont.owl#hasCurrentDiastolicBP> rdf:type owl:DatatypeProperty .

### http://www.co-ode.org/ontologies/uia/ont.owl#hasCurrentHDLLevel

<http://www.co-ode.org/ontologies/uia/ont.owl#hasCurrentHDLLevel> rdf:type owl:DatatypeProperty .

### http://www.co-ode.org/ontologies/uia/ont.owl#hasCurrentHeartRate

<http://www.co-ode.org/ontologies/uia/ont.owl#hasCurrentHeartRate> rdf:type owl:DatatypeProperty ;

rdfs:domain <http://www.co-ode.org/ontologies/uia/ont.owl#ActivityDataValue> ;

rdfs:range xsd:integer .

### http://www.co-ode.org/ontologies/uia/ont.owl#hasCurrentLDLLevel

<http://www.co-ode.org/ontologies/uia/ont.owl#hasCurrentLDLLevel> rdf:type owl:DatatypeProperty .

### http://www.co-ode.org/ontologies/uia/ont.owl#hasCurrentSystolicBP

<http://www.co-ode.org/ontologies/uia/ont.owl#hasCurrentSystolicBP> rdf:type owl:DatatypeProperty .

### http://www.co-ode.org/ontologies/uia/ont.owl#hasDailyWeight

<http://www.co-ode.org/ontologies/uia/ont.owl#hasDailyWeight> rdf:type owl:DatatypeProperty .

### http://www.co-ode.org/ontologies/uia/ont.owl#hasDateTime

<http://www.co-ode.org/ontologies/uia/ont.owl#hasDateTime> rdf:type owl:DatatypeProperty ;

rdfs:domain <http://www.co-ode.org/ontologies/uia/ont.owl#TemporalEntity> ;

rdfs:range xsd:dateTimeStamp .

### http://www.co-ode.org/ontologies/uia/ont.owl#hasDepartment

<http://www.co-ode.org/ontologies/uia/ont.owl#hasDepartment> rdf:type owl:DatatypeProperty ;

rdfs:domain <http://www.co-ode.org/ontologies/uia/ont.owl#PersonalData> ;

rdfs:range xsd:string .

### http://www.co-ode.org/ontologies/uia/ont.owl#hasDesignation

<http://www.co-ode.org/ontologies/uia/ont.owl#hasDesignation> rdf:type owl:DatatypeProperty ;

rdfs:domain <http://www.co-ode.org/ontologies/uia/ont.owl#PersonalData> ;

rdfs:range xsd:string .

### http://www.co-ode.org/ontologies/uia/ont.owl#hasDetectionLimit

<http://www.co-ode.org/ontologies/uia/ont.owl#hasDetectionLimit> rdf:type owl:DatatypeProperty ;

rdfs:domain ssn:DetectionLimit ;

rdfs:range xsd:string .

### http://www.co-ode.org/ontologies/uia/ont.owl#hasDistanceCovered

<http://www.co-ode.org/ontologies/uia/ont.owl#hasDistanceCovered> rdf:type owl:DatatypeProperty .

### http://www.co-ode.org/ontologies/uia/ont.owl#hasDrift

<http://www.co-ode.org/ontologies/uia/ont.owl#hasDrift> rdf:type owl:DatatypeProperty ;

rdfs:domain ssn:Drift ;

rdfs:range xsd:string .

### http://www.co-ode.org/ontologies/uia/ont.owl#hasDurationOfIntensiveActivity

<http://www.co-ode.org/ontologies/uia/ont.owl#hasDurationOfIntensiveActivity> rdf:type owl:DatatypeProperty .

### http://www.co-ode.org/ontologies/uia/ont.owl#hasEducationalLevel

<http://www.co-ode.org/ontologies/uia/ont.owl#hasEducationalLevel> rdf:type owl:DatatypeProperty .

### http://www.co-ode.org/ontologies/uia/ont.owl#hasEmail

<http://www.co-ode.org/ontologies/uia/ont.owl#hasEmail> rdf:type owl:DatatypeProperty ;

rdfs:domain <http://www.co-ode.org/ontologies/uia/ont.owl#PersonalData> ;

rdfs:range xsd:string .

### http://www.co-ode.org/ontologies/uia/ont.owl#hasFirstName

<http://www.co-ode.org/ontologies/uia/ont.owl#hasFirstName> rdf:type owl:DatatypeProperty ;

rdfs:domain <http://www.co-ode.org/ontologies/uia/ont.owl#PersonalData> ;

rdfs:range xsd:string .

### http://www.co-ode.org/ontologies/uia/ont.owl#hasFoggyForeCast

<http://www.co-ode.org/ontologies/uia/ont.owl#hasFoggyForeCast> rdf:type owl:DatatypeProperty ;

rdfs:domain <http://www.co-ode.org/ontologies/uia/ont.owl#ExternalWeatherValue> ;

rdfs:range xsd:string .

### http://www.co-ode.org/ontologies/uia/ont.owl#hasFrequency

<http://www.co-ode.org/ontologies/uia/ont.owl#hasFrequency> rdf:type owl:DatatypeProperty ;

rdfs:domain ssn:Frequency ;

rdfs:range xsd:string .

### http://www.co-ode.org/ontologies/uia/ont.owl#hasFruits

<http://www.co-ode.org/ontologies/uia/ont.owl#hasFruits> rdf:type owl:DatatypeProperty .

### http://www.co-ode.org/ontologies/uia/ont.owl#hasGender

<http://www.co-ode.org/ontologies/uia/ont.owl#hasGender> rdf:type owl:DatatypeProperty ;

rdfs:domain <http://www.co-ode.org/ontologies/uia/ont.owl#PersonalData> ;

rdfs:range xsd:string .

### http://www.co-ode.org/ontologies/uia/ont.owl#hasHeartRate

<http://www.co-ode.org/ontologies/uia/ont.owl#hasHeartRate> rdf:type owl:DatatypeProperty .

### http://www.co-ode.org/ontologies/uia/ont.owl#hasHeight

<http://www.co-ode.org/ontologies/uia/ont.owl#hasHeight> rdf:type owl:DatatypeProperty .

### http://www.co-ode.org/ontologies/uia/ont.owl#hasHighTemperatureForeCast

<http://www.co-ode.org/ontologies/uia/ont.owl#hasHighTemperatureForeCast> rdf:type owl:DatatypeProperty .

### http://www.co-ode.org/ontologies/uia/ont.owl#hasIncomeGroup

<http://www.co-ode.org/ontologies/uia/ont.owl#hasIncomeGroup> rdf:type owl:DatatypeProperty .

### http://www.co-ode.org/ontologies/uia/ont.owl#hasKommuneName

<http://www.co-ode.org/ontologies/uia/ont.owl#hasKommuneName> rdf:type owl:DatatypeProperty ;

rdfs:domain <http://www.co-ode.org/ontologies/uia/ont.owl#OfficeAddress> ;

rdfs:range xsd:string .

### http://www.co-ode.org/ontologies/uia/ont.owl#hasLastName

<http://www.co-ode.org/ontologies/uia/ont.owl#hasLastName> rdf:type owl:DatatypeProperty ;

rdfs:domain <http://www.co-ode.org/ontologies/uia/ont.owl#PersonalData> ;

rdfs:range xsd:string .

### http://www.co-ode.org/ontologies/uia/ont.owl#hasLatency

<http://www.co-ode.org/ontologies/uia/ont.owl#hasLatency> rdf:type owl:DatatypeProperty ;

rdfs:domain ssn:Latency ;

rdfs:range xsd:string .

### http://www.co-ode.org/ontologies/uia/ont.owl#hasLowTemperatureForeCast

<http://www.co-ode.org/ontologies/uia/ont.owl#hasLowTemperatureForeCast> rdf:type owl:DatatypeProperty .

### http://www.co-ode.org/ontologies/uia/ont.owl#hasMeasurementRange

<http://www.co-ode.org/ontologies/uia/ont.owl#hasMeasurementRange> rdf:type owl:DatatypeProperty ;

rdfs:domain ssn:MeasurementRange ;

rdfs:range xsd:string .

### http://www.co-ode.org/ontologies/uia/ont.owl#hasMetabolicRate

<http://www.co-ode.org/ontologies/uia/ont.owl#hasMetabolicRate> rdf:type owl:DatatypeProperty ;

rdfs:domain <http://www.co-ode.org/ontologies/uia/ont.owl#ActivityDataValue> ;

rdfs:range xsd:integer .

### http://www.co-ode.org/ontologies/uia/ont.owl#hasMobile

<http://www.co-ode.org/ontologies/uia/ont.owl#hasMobile> rdf:type owl:DatatypeProperty ;

rdfs:domain <http://www.co-ode.org/ontologies/uia/ont.owl#PersonalData> ;

rdfs:range xsd:string .

### http://www.co-ode.org/ontologies/uia/ont.owl#hasMoodInEvening

<http://www.co-ode.org/ontologies/uia/ont.owl#hasMoodInEvening> rdf:type owl:DatatypeProperty .

### http://www.co-ode.org/ontologies/uia/ont.owl#hasMoodInMorning

<http://www.co-ode.org/ontologies/uia/ont.owl#hasMoodInMorning> rdf:type owl:DatatypeProperty .

### http://www.co-ode.org/ontologies/uia/ont.owl#hasMoodInNight

<http://www.co-ode.org/ontologies/uia/ont.owl#hasMoodInNight> rdf:type owl:DatatypeProperty .

### http://www.co-ode.org/ontologies/uia/ont.owl#hasName

<http://www.co-ode.org/ontologies/uia/ont.owl#hasName> rdf:type owl:DatatypeProperty ;

rdfs:domain <http://www.co-ode.org/ontologies/uia/ont.owl#SimulatedInputDataSequence> ;

rdfs:range xsd:string .

### http://www.co-ode.org/ontologies/uia/ont.owl#hasNextAppointment

<http://www.co-ode.org/ontologies/uia/ont.owl#hasNextAppointment> rdf:type owl:DatatypeProperty ;

rdfs:domain <http://www.co-ode.org/ontologies/uia/ont.owl#TemporalEntity> ;

rdfs:range xsd:dateTimeStamp .

### http://www.co-ode.org/ontologies/uia/ont.owl#hasNonWearDeviceTime

<http://www.co-ode.org/ontologies/uia/ont.owl#hasNonWearDeviceTime> rdf:type owl:DatatypeProperty .

### http://www.co-ode.org/ontologies/uia/ont.owl#hasOfficePhone

<http://www.co-ode.org/ontologies/uia/ont.owl#hasOfficePhone> rdf:type owl:DatatypeProperty ;

rdfs:subPropertyOf owl:topDataProperty ;

rdfs:domain <http://www.co-ode.org/ontologies/uia/ont.owl#OfficeAddress> ;

rdfs:range xsd:string .

### http://www.co-ode.org/ontologies/uia/ont.owl#hasOfficePostCode

<http://www.co-ode.org/ontologies/uia/ont.owl#hasOfficePostCode> rdf:type owl:DatatypeProperty ;

rdfs:subPropertyOf owl:topDataProperty ;

rdfs:domain <http://www.co-ode.org/ontologies/uia/ont.owl#OfficeAddress> ;

rdfs:range xsd:integer .

### http://www.co-ode.org/ontologies/uia/ont.owl#hasOperatingPowerRange

<http://www.co-ode.org/ontologies/uia/ont.owl#hasOperatingPowerRange> rdf:type owl:DatatypeProperty ;

rdfs:subPropertyOf owl:topDataProperty ;

rdfs:domain ssn:OperatingPowerRange ;

rdfs:range xsd:string .

### http://www.co-ode.org/ontologies/uia/ont.owl#hasParticipationStatus

<http://www.co-ode.org/ontologies/uia/ont.owl#hasParticipationStatus> rdf:type owl:DatatypeProperty ;

rdfs:subPropertyOf owl:topDataProperty ;

rdfs:domain <http://www.co-ode.org/ontologies/uia/ont.owl#ParticipantStatus> ;

rdfs:range xsd:string .

### http://www.co-ode.org/ontologies/uia/ont.owl#hasPassword

<http://www.co-ode.org/ontologies/uia/ont.owl#hasPassword> rdf:type owl:DatatypeProperty ;

rdfs:subPropertyOf owl:topDataProperty ;

rdfs:domain <http://www.co-ode.org/ontologies/uia/ont.owl#Human> ;

rdfs:range xsd:string .

### http://www.co-ode.org/ontologies/uia/ont.owl#hasPhysicalActivityIntensity

<http://www.co-ode.org/ontologies/uia/ont.owl#hasPhysicalActivityIntensity> rdf:type owl:DatatypeProperty ;

rdfs:subPropertyOf owl:topDataProperty ;

rdfs:domain <http://www.co-ode.org/ontologies/uia/ont.owl#ActivityDataValue> ;

rdfs:range xsd:integer .

### http://www.co-ode.org/ontologies/uia/ont.owl#hasPhysicalActivityType

<http://www.co-ode.org/ontologies/uia/ont.owl#hasPhysicalActivityType> rdf:type owl:DatatypeProperty ;

rdfs:subPropertyOf owl:topDataProperty ;

rdfs:domain <http://www.co-ode.org/ontologies/uia/ont.owl#ActivityDataValue> ;

rdfs:range xsd:string .

### http://www.co-ode.org/ontologies/uia/ont.owl#hasPostCode

<http://www.co-ode.org/ontologies/uia/ont.owl#hasPostCode> rdf:type owl:DatatypeProperty .

### http://www.co-ode.org/ontologies/uia/ont.owl#hasPrecisionValue

<http://www.co-ode.org/ontologies/uia/ont.owl#hasPrecisionValue> rdf:type owl:DatatypeProperty ;

rdfs:subPropertyOf owl:topDataProperty ;

rdfs:domain ssn:Precision ;

rdfs:range xsd:string .

### http://www.co-ode.org/ontologies/uia/ont.owl#hasRainingForeCast

<http://www.co-ode.org/ontologies/uia/ont.owl#hasRainingForeCast> rdf:type owl:DatatypeProperty ;

rdfs:domain <http://www.co-ode.org/ontologies/uia/ont.owl#ExternalWeatherValue> ;

rdfs:range xsd:string .

### http://www.co-ode.org/ontologies/uia/ont.owl#hasResolution

<http://www.co-ode.org/ontologies/uia/ont.owl#hasResolution> rdf:type owl:DatatypeProperty ;

rdfs:domain ssn:Resolution ;

rdfs:range xsd:string .

### http://www.co-ode.org/ontologies/uia/ont.owl#hasResponseTime

<http://www.co-ode.org/ontologies/uia/ont.owl#hasResponseTime> rdf:type owl:DatatypeProperty ;

rdfs:domain ssn:ResponseTime ;

rdfs:range xsd:string .

### http://www.co-ode.org/ontologies/uia/ont.owl#hasRole

<http://www.co-ode.org/ontologies/uia/ont.owl#hasRole> rdf:type owl:DatatypeProperty ;

rdfs:domain <http://www.co-ode.org/ontologies/uia/ont.owl#Human> ;

rdfs:range xsd:string .

### http://www.co-ode.org/ontologies/uia/ont.owl#hasRoomNo

<http://www.co-ode.org/ontologies/uia/ont.owl#hasRoomNo> rdf:type owl:DatatypeProperty ;

rdfs:domain <http://www.co-ode.org/ontologies/uia/ont.owl#OfficeAddress> ;

rdfs:range xsd:string .

### http://www.co-ode.org/ontologies/uia/ont.owl#hasSCTID

<http://www.co-ode.org/ontologies/uia/ont.owl#hasSCTID> rdf:type owl:DatatypeProperty ;

rdfs:domain <http://www.co-ode.org/ontologies/uia/ont.owl#SNOMED_CT_Concept> ;

rdfs:range xsd:long .

### http://www.co-ode.org/ontologies/uia/ont.owl#hasSedentaryBouts

<http://www.co-ode.org/ontologies/uia/ont.owl#hasSedentaryBouts> rdf:type owl:DatatypeProperty ;

rdfs:domain <http://www.co-ode.org/ontologies/uia/ont.owl#ActivityDataValue> ;

rdfs:range xsd:integer .

### http://www.co-ode.org/ontologies/uia/ont.owl#hasSelectivity

<http://www.co-ode.org/ontologies/uia/ont.owl#hasSelectivity> rdf:type owl:DatatypeProperty ;

rdfs:domain ssn:Selectivity ;

rdfs:range xsd:string .

### http://www.co-ode.org/ontologies/uia/ont.owl#hasSensitivity

<http://www.co-ode.org/ontologies/uia/ont.owl#hasSensitivity> rdf:type owl:DatatypeProperty ;

rdfs:domain ssn:Sensitivity ;

rdfs:range xsd:string .

### http://www.co-ode.org/ontologies/uia/ont.owl#hasSmokingHabit

<http://www.co-ode.org/ontologies/uia/ont.owl#hasSmokingHabit> rdf:type owl:DatatypeProperty .

### http://www.co-ode.org/ontologies/uia/ont.owl#hasSnowingForeCast

<http://www.co-ode.org/ontologies/uia/ont.owl#hasSnowingForeCast> rdf:type owl:DatatypeProperty ;

rdfs:domain <http://www.co-ode.org/ontologies/uia/ont.owl#ExternalWeatherValue> ;

rdfs:range xsd:string .

### http://www.co-ode.org/ontologies/uia/ont.owl#hasSnusHabit

<http://www.co-ode.org/ontologies/uia/ont.owl#hasSnusHabit> rdf:type owl:DatatypeProperty .

### http://www.co-ode.org/ontologies/uia/ont.owl#hasSocialParticipationType

<http://www.co-ode.org/ontologies/uia/ont.owl#hasSocialParticipationType> rdf:type owl:DatatypeProperty .

### http://www.co-ode.org/ontologies/uia/ont.owl#hasSoialParticipationFrequency

<http://www.co-ode.org/ontologies/uia/ont.owl#hasSoialParticipationFrequency> rdf:type owl:DatatypeProperty .

### http://www.co-ode.org/ontologies/uia/ont.owl#hasSteps

<http://www.co-ode.org/ontologies/uia/ont.owl#hasSteps> rdf:type owl:DatatypeProperty ;

rdfs:domain <http://www.co-ode.org/ontologies/uia/ont.owl#ActivityDataValue> ;

rdfs:range xsd:integer .

### http://www.co-ode.org/ontologies/uia/ont.owl#hasSunnyForeCast

<http://www.co-ode.org/ontologies/uia/ont.owl#hasSunnyForeCast> rdf:type owl:DatatypeProperty ;

rdfs:domain <http://www.co-ode.org/ontologies/uia/ont.owl#ExternalWeatherValue> ;

rdfs:range xsd:string .

### http://www.co-ode.org/ontologies/uia/ont.owl#hasTemperature

<http://www.co-ode.org/ontologies/uia/ont.owl#hasTemperature> rdf:type owl:DatatypeProperty .

### http://www.co-ode.org/ontologies/uia/ont.owl#hasTotalSleepTime

<http://www.co-ode.org/ontologies/uia/ont.owl#hasTotalSleepTime> rdf:type owl:DatatypeProperty ;

rdfs:domain <http://www.co-ode.org/ontologies/uia/ont.owl#ActivityDataValue> ;

rdfs:range xsd:integer .

### http://www.co-ode.org/ontologies/uia/ont.owl#hasVegetables

<http://www.co-ode.org/ontologies/uia/ont.owl#hasVegetables> rdf:type owl:DatatypeProperty .

### http://www.co-ode.org/ontologies/uia/ont.owl#hasWeatherSatus

<http://www.co-ode.org/ontologies/uia/ont.owl#hasWeatherSatus> rdf:type owl:DatatypeProperty ;

rdfs:domain <http://www.co-ode.org/ontologies/uia/ont.owl#ExternalWeatherValue> ;

rdfs:range xsd:string .

### http://www.co-ode.org/ontologies/uia/ont.owl#sCTID

<http://www.co-ode.org/ontologies/uia/ont.owl#sCTID> rdf:type owl:DatatypeProperty ;

rdfs:domain <http://www.co-ode.org/ontologies/uia/ont.owl#SNOWMEDCTSimulatedinput> ;

rdfs:range xsd:string .

#################################################################

# Classes

#################################################################

### http://purl.oclc.org/NET/ssnx/ssn#Accuracy

ssn:Accuracy rdf:type owl:Class ;

owl:equivalentClass [ rdf:type owl:Restriction ;

owl:onProperty <http://www.co-ode.org/ontologies/uia/ont.owl#hasAccuracy> ;

owl:maxQualifiedCardinality "1"^^xsd:nonNegativeInteger ;

owl:onDataRange xsd:string

] ;

rdfs:subClassOf ssn:MeasurementProperty ;

dc:source """skos:exactMatch 'measurement accuracy/accuracy' [VIM 2.13]

http://www.bipm.org/utils/common/documents/jcgm/JCGM_200_2008.pdf""" ;

rdfs:comment "The closeness of agreement between the value of an observation and the true value of the observed quality." ;

rdfs:isDefinedBy "http://purl.oclc.org/NET/ssnx/ssn" ;

rdfs:label "Accuracy" ;

rdfs:seeAlso "http://www.w3.org/2005/Incubator/ssn/wiki/SSN_Sensor#MeasuringCapability" .

### http://purl.oclc.org/NET/ssnx/ssn#BatteryLifetime

ssn:BatteryLifetime rdf:type owl:Class ;

rdfs:subClassOf ssn:SurvivalProperty ;

rdfs:comment "Total useful life of a battery." ;

rdfs:isDefinedBy "http://purl.oclc.org/NET/ssnx/ssn" ;

rdfs:label "Battery Lifetime" ;

rdfs:seeAlso "http://www.w3.org/2005/Incubator/ssn/wiki/SSN_Energy#EnergyRestriction" .

### http://purl.oclc.org/NET/ssnx/ssn#Condition

ssn:Condition rdf:type owl:Class ;

owl:equivalentClass [ rdf:type owl:Restriction ;

owl:onProperty <http://www.co-ode.org/ontologies/uia/ont.owl#hasCondition> ;

owl:qualifiedCardinality "1"^^xsd:nonNegativeInteger ;

owl:onDataRange xsd:string

] ;

rdfs:subClassOf ssn:Property ;

rdfs:comment "Used to specify ranges for qualities that act as conditions on a system/sensor's operation. For example, wind speed of 10-60m/s is expressed as a condition linking a quality, wind speed, a unit of measurement, metres per second, and a set of values, 10-60, and may be used as the condition on a MeasurementProperty, for example, to state that a sensor has a particular accuracy in that condition." ;

rdfs:isDefinedBy "http://purl.oclc.org/NET/ssnx/ssn" ;

rdfs:label "Condition" ;

rdfs:seeAlso "http://www.w3.org/2005/Incubator/ssn/wiki/SSN_Base#ConstraintBlock" .

### http://purl.oclc.org/NET/ssnx/ssn#DailyActivityData

ssn:DailyActivityData rdf:type owl:Class ;

rdfs:subClassOf <http://www.co-ode.org/ontologies/uia/ont.owl#Questionnaire> ,

[ rdf:type owl:Restriction ;

owl:onProperty <http://www.co-ode.org/ontologies/uia/ont.owl#hasDailyWeight> ;

owl:qualifiedCardinality "1"^^xsd:nonNegativeInteger ;

owl:onDataRange xsd:double

] ,

[ rdf:type owl:Restriction ;

owl:onProperty <http://www.co-ode.org/ontologies/uia/ont.owl#hasDurationOfIntensiveActivity> ;

owl:qualifiedCardinality "1"^^xsd:nonNegativeInteger ;

owl:onDataRange xsd:integer

] ,

[ rdf:type owl:Restriction ;

owl:onProperty <http://www.co-ode.org/ontologies/uia/ont.owl#hasMoodInEvening> ;

owl:qualifiedCardinality "1"^^xsd:nonNegativeInteger ;

owl:onDataRange xsd:integer

] ,

[ rdf:type owl:Restriction ;

owl:onProperty <http://www.co-ode.org/ontologies/uia/ont.owl#hasMoodInMorning> ;

owl:qualifiedCardinality "1"^^xsd:nonNegativeInteger ;

owl:onDataRange xsd:integer

] ,

[ rdf:type owl:Restriction ;

owl:onProperty <http://www.co-ode.org/ontologies/uia/ont.owl#hasMoodInNight> ;

owl:qualifiedCardinality "1"^^xsd:nonNegativeInteger ;

owl:onDataRange xsd:integer

] ,

[ rdf:type owl:Restriction ;

owl:onProperty <http://www.co-ode.org/ontologies/uia/ont.owl#hasNonWearDeviceTime> ;

owl:qualifiedCardinality "1"^^xsd:nonNegativeInteger ;

owl:onDataRange xsd:integer

] .

### http://purl.oclc.org/NET/ssnx/ssn#DailyHabitData

ssn:DailyHabitData rdf:type owl:Class ;

rdfs:subClassOf <http://www.co-ode.org/ontologies/uia/ont.owl#Questionnaire> ,

[ rdf:type owl:Restriction ;

owl:onProperty ssn:hasAlcoholQuantity ;

owl:qualifiedCardinality "1"^^xsd:nonNegativeInteger ;

owl:onDataRange xsd:integer

] ,

[ rdf:type owl:Restriction ;

owl:onProperty ssn:hasConsumedAlcohol ;

owl:qualifiedCardinality "1"^^xsd:nonNegativeInteger ;

owl:onDataRange xsd:string

] ,

[ rdf:type owl:Restriction ;

owl:onProperty ssn:hasSmoked ;

owl:qualifiedCardinality "1"^^xsd:nonNegativeInteger ;

owl:onDataRange xsd:string

] ,

[ rdf:type owl:Restriction ;

owl:onProperty ssn:hasSnusQuantity ;

owl:qualifiedCardinality "1"^^xsd:nonNegativeInteger ;

owl:onDataRange xsd:integer

] ,

[ rdf:type owl:Restriction ;

owl:onProperty ssn:hasTakenSnus ;

owl:qualifiedCardinality "1"^^xsd:nonNegativeInteger ;

owl:onDataRange xsd:string

] ,

[ rdf:type owl:Restriction ;

owl:onProperty ssn:hasTobaccoCount ;

owl:qualifiedCardinality "1"^^xsd:nonNegativeInteger ;

owl:onDataRange xsd:integer

] .

### http://purl.oclc.org/NET/ssnx/ssn#DailyNutritionData

ssn:DailyNutritionData rdf:type owl:Class ;

rdfs:subClassOf <http://www.co-ode.org/ontologies/uia/ont.owl#Questionnaire> ,

[ rdf:type owl:Restriction ;

owl:onProperty ssn:hasFriedorProcessedFood ;

owl:qualifiedCardinality "1"^^xsd:nonNegativeInteger ;

owl:onDataRange xsd:string

] ,

[ rdf:type owl:Restriction ;

owl:onProperty ssn:hasFriedorProcessedFoodFrequency ;

owl:qualifiedCardinality "1"^^xsd:nonNegativeInteger ;

owl:onDataRange xsd:integer

] ,

[ rdf:type owl:Restriction ;

owl:onProperty ssn:hasFruitAmount ;

owl:qualifiedCardinality "1"^^xsd:nonNegativeInteger ;

owl:onDataRange xsd:integer

] ,

[ rdf:type owl:Restriction ;

owl:onProperty ssn:hasSweetBakeries ;

owl:qualifiedCardinality "1"^^xsd:nonNegativeInteger ;

owl:onDataRange xsd:string

] ,

[ rdf:type owl:Restriction ;

owl:onProperty ssn:hasSweetBakeriesFrequency ;

owl:qualifiedCardinality "1"^^xsd:nonNegativeInteger ;

owl:onDataRange xsd:integer

] ,

[ rdf:type owl:Restriction ;

owl:onProperty ssn:hasSweetBeverages ;

owl:qualifiedCardinality "1"^^xsd:nonNegativeInteger ;

owl:onDataRange xsd:string

] ,

[ rdf:type owl:Restriction ;

owl:onProperty ssn:hasSweetBeveragesAmount ;

owl:qualifiedCardinality "1"^^xsd:nonNegativeInteger ;

owl:onDataRange xsd:integer

] ,

[ rdf:type owl:Restriction ;

owl:onProperty ssn:hasSweetFoodorMilkProduct ;

owl:qualifiedCardinality "1"^^xsd:nonNegativeInteger ;

owl:onDataRange xsd:string

] ,

[ rdf:type owl:Restriction ;

owl:onProperty ssn:hasSweetFoodorMilkProductFrequency ;

owl:qualifiedCardinality "1"^^xsd:nonNegativeInteger ;

owl:onDataRange xsd:integer

] ,

[ rdf:type owl:Restriction ;

owl:onProperty ssn:hasVegetableAmount ;

owl:qualifiedCardinality "1"^^xsd:nonNegativeInteger ;

owl:onDataRange xsd:integer

] ,

[ rdf:type owl:Restriction ;

owl:onProperty <http://www.co-ode.org/ontologies/uia/ont.owl#hasFruits> ;

owl:qualifiedCardinality "1"^^xsd:nonNegativeInteger ;

owl:onDataRange xsd:string

] ,

[ rdf:type owl:Restriction ;

owl:onProperty <http://www.co-ode.org/ontologies/uia/ont.owl#hasVegetables> ;

owl:qualifiedCardinality "1"^^xsd:nonNegativeInteger ;

owl:onDataRange xsd:string

] .

### http://purl.oclc.org/NET/ssnx/ssn#DailyPhysiologicalData

ssn:DailyPhysiologicalData rdf:type owl:Class ;

rdfs:subClassOf <http://www.co-ode.org/ontologies/uia/ont.owl#Questionnaire> ,

[ rdf:type owl:Restriction ;

owl:onProperty <http://www.co-ode.org/ontologies/uia/ont.owl#hasCurrentBMIValue> ;

owl:qualifiedCardinality "1"^^xsd:nonNegativeInteger ;

owl:onDataRange xsd:double

] .

### http://purl.oclc.org/NET/ssnx/ssn#Deployment

ssn:Deployment rdf:type owl:Class ;

rdfs:subClassOf ssn:DeploymentRelatedProcess ,

[ rdf:type owl:Restriction ;

owl:onProperty ssn:deployedOnPlatform ;

owl:allValuesFrom ssn:Platform

] ,

[ rdf:type owl:Restriction ;

owl:onProperty ssn:deployedSystem ;

owl:allValuesFrom ssn:System

] ;

dc:source """skos:closeMatch 'Deployment' [MMI Dev]

http://marinemetadata.org/community/teams/ontdevices""" ;

rdfs:comment "The ongoing Process of Entities (for the purposes of this ontology, mainly sensors) deployed for a particular purpose. For example, a particular Sensor deployed on a Platform, or a whole network of Sensors deployed for an observation campaign. The deployment may have sub processes, such as installation, maintenance, addition, and decomissioning and removal." ;

rdfs:isDefinedBy "http://purl.oclc.org/NET/ssnx/ssn" ;

rdfs:label "Deployment" ;

rdfs:seeAlso "http://www.w3.org/2005/Incubator/ssn/wiki/SSN_Deploy#Deployment" .

### http://purl.oclc.org/NET/ssnx/ssn#DeploymentRelatedProcess

ssn:DeploymentRelatedProcess rdf:type owl:Class ;

rdfs:subClassOf DUL:Process ,

[ rdf:type owl:Restriction ;

owl:onProperty ssn:deploymentProcessPart ;

owl:allValuesFrom ssn:DeploymentRelatedProcess

] ;

dc:source "http://www.w3.org/2005/Incubator/ssn/" ;

rdfs:comment "Place to group all the various Processes related to Deployment. For example, as well as Deplyment, installation, maintenance, deployment of further sensors and the like would all be classified under DeploymentRelatedProcess." ;

rdfs:isDefinedBy "http://purl.oclc.org/NET/ssnx/ssn" ;

rdfs:label "Deployment-related Process" ;

rdfs:seeAlso "http://www.w3.org/2005/Incubator/ssn/wiki/SSN_Deploy#Deployment" .

### http://purl.oclc.org/NET/ssnx/ssn#DetectionLimit

ssn:DetectionLimit rdf:type owl:Class ;

owl:equivalentClass [ rdf:type owl:Restriction ;

owl:onProperty <http://www.co-ode.org/ontologies/uia/ont.owl#hasDetectionLimit> ;

owl:maxQualifiedCardinality "1"^^xsd:nonNegativeInteger ;

owl:onDataRange xsd:string

] ;

rdfs:subClassOf ssn:MeasurementProperty ;

dc:source """skos:exactMatch 'detection limit' [VIM 4.18]

http://www.bipm.org/utils/common/documents/jcgm/JCGM_200_2008.pdf""" ;

rdfs:comment "An observed value for which the probability of falsely claiming the absence of a component in a material is Î², given a probability Î± of falsely claiming its presence." ;

rdfs:isDefinedBy "http://purl.oclc.org/NET/ssnx/ssn" ;

rdfs:label "detection limit" ;

rdfs:seeAlso "http://www.w3.org/2005/Incubator/ssn/wiki/SSN_Sensor#MeasuringCapability" .

### http://purl.oclc.org/NET/ssnx/ssn#Drift

ssn:Drift rdf:type owl:Class ;

owl:equivalentClass [ rdf:type owl:Restriction ;

owl:onProperty <http://www.co-ode.org/ontologies/uia/ont.owl#hasDrift> ;

owl:maxQualifiedCardinality "1"^^xsd:nonNegativeInteger ;

owl:onDataRange xsd:string

] ;

rdfs:subClassOf ssn:MeasurementProperty ;

dc:source """skos:exactMatch 'instrumental drift' [VIM 4.21]

http://www.bipm.org/utils/common/documents/jcgm/JCGM_200_2008.pdf""" ;

rdfs:comment "A, continuous or incremental, change in the reported values of observations over time for an unchanging quality." ;

rdfs:isDefinedBy "http://purl.oclc.org/NET/ssnx/ssn" ;

rdfs:label "Drift" ;

rdfs:seeAlso "http://www.w3.org/2005/Incubator/ssn/wiki/SSN_Sensor#MeasuringCapability" .

### http://purl.oclc.org/NET/ssnx/ssn#FeatureOfInterest

ssn:FeatureOfInterest rdf:type owl:Class ;

rdfs:subClassOf <http://www.co-ode.org/ontologies/uia/ont.owl#PhysicalDeviceThing> ,

[ rdf:type owl:Restriction ;

owl:onProperty ssn:hasProperty ;

owl:someValuesFrom ssn:Property

] ,

[ rdf:type owl:Restriction ;

owl:onProperty ssn:hasProperty ;

owl:allValuesFrom ssn:Property

] ;

dc:source """skos:exactMatch 'feature' [O&M]

http://www.opengeospatial.org/standards/om""" ;

rdfs:comment "A feature is an abstraction of real world phenomena (thing, person, event, etc)." ;

rdfs:isDefinedBy "http://purl.oclc.org/NET/ssnx/ssn" ;

rdfs:label "Feature of Interest" ;

rdfs:seeAlso "http://www.w3.org/2005/Incubator/ssn/wiki/SSN_Skeleton#Skeleton" .

### http://purl.oclc.org/NET/ssnx/ssn#Frequency

ssn:Frequency rdf:type owl:Class ;

owl:equivalentClass [ rdf:type owl:Restriction ;

owl:onProperty <http://www.co-ode.org/ontologies/uia/ont.owl#hasFrequency> ;

owl:maxQualifiedCardinality "1"^^xsd:nonNegativeInteger ;

owl:onDataRange xsd:string

] ;

rdfs:subClassOf ssn:MeasurementProperty ;

rdfs:comment "The smallest possible time between one observation and the next." ;

rdfs:isDefinedBy "http://purl.oclc.org/NET/ssnx/ssn" ;

rdfs:label "Frequency" ;

rdfs:seeAlso "http://www.w3.org/2005/Incubator/ssn/wiki/SSN_Sensor#MeasuringCapability" .

### http://purl.oclc.org/NET/ssnx/ssn#Latency

ssn:Latency rdf:type owl:Class ;

owl:equivalentClass [ rdf:type owl:Restriction ;

owl:onProperty <http://www.co-ode.org/ontologies/uia/ont.owl#hasLatency> ;

owl:maxQualifiedCardinality "1"^^xsd:nonNegativeInteger ;

owl:onDataRange xsd:string

] ;

rdfs:subClassOf ssn:MeasurementProperty ;

rdfs:comment "The time between a request for an observation and the sensor providing a result." ;

rdfs:isDefinedBy "http://purl.oclc.org/NET/ssnx/ssn" ;

rdfs:label "Latency" ;

rdfs:seeAlso "http://www.w3.org/2005/Incubator/ssn/wiki/SSN_Sensor#MeasuringCapability" .

### http://purl.oclc.org/NET/ssnx/ssn#MaintenanceSchedule

ssn:MaintenanceSchedule rdf:type owl:Class ;

rdfs:subClassOf ssn:OperatingProperty ,

[ rdf:type owl:Restriction ;

owl:onProperty <http://www.co-ode.org/ontologies/uia/ont.owl#hasTimeStamp> ;

owl:someValuesFrom <http://www.co-ode.org/ontologies/uia/ont.owl#TemporalEntity>

] ;

rdfs:comment "Schedule of maintenance for a system/sensor in the specified conditions." ;

rdfs:isDefinedBy "http://purl.oclc.org/NET/ssnx/ssn" ;

rdfs:label "Maintenance Schedule" ;

rdfs:seeAlso "http://www.w3.org/2005/Incubator/ssn/wiki/SSN_Deploy#OperatingRestriction" .

### http://purl.oclc.org/NET/ssnx/ssn#MeasurementCapability

ssn:MeasurementCapability rdf:type owl:Class ;

rdfs:subClassOf ssn:Property ,

[ rdf:type owl:Restriction ;

owl:onProperty ssn:forProperty ;

owl:allValuesFrom ssn:Property

] ,

[ rdf:type owl:Restriction ;

owl:onProperty ssn:hasMeasurementProperty ;

owl:allValuesFrom ssn:MeasurementProperty

] ,

[ rdf:type owl:Restriction ;

owl:onProperty ssn:inCondition ;

owl:allValuesFrom ssn:Condition

] ;

dc:source """Similar idea to MeasurementCapability in MMI Device Ontology

http://marinemetadata.org/community/teams/ontdevices

But the the two express the relationship between constraints and multiple measurement properties differently.

The conditions linked to a MeasurementCapability are skos:exactMatch to 'influence quantity' [VIM 2.52]

http://www.bipm.org/utils/common/documents/jcgm/JCGM_200_2008.pdf""" ;

rdfs:comment """Collects together measurement properties (accuracy, range, precision, etc) and the environmental conditions in which those properties hold, representing a specification of a sensor's capability in those conditions.

The conditions specified here are those that affect the measurement properties, while those in OperatingRange represent the sensor's standard operating conditions, including conditions that don't affect the observations.""" ;

rdfs:isDefinedBy "http://purl.oclc.org/NET/ssnx/ssn" ;

rdfs:label "Measurement Capability" ;

rdfs:seeAlso "http://www.w3.org/2005/Incubator/ssn/wiki/SSN_Sensor#MeasuringCapability" .

### http://purl.oclc.org/NET/ssnx/ssn#MeasurementProperty

ssn:MeasurementProperty rdf:type owl:Class ;

rdfs:subClassOf ssn:Property ;

rdfs:comment "An identifiable and observable characteristic of a sensor's observations or ability to make observations." ;

rdfs:isDefinedBy "http://purl.oclc.org/NET/ssnx/ssn" ;

rdfs:label "Measurement Property" ;

rdfs:seeAlso "http://www.w3.org/2005/Incubator/ssn/wiki/SSN_Sensor#MeasuringCapability" .

### http://purl.oclc.org/NET/ssnx/ssn#MeasurementRange

ssn:MeasurementRange rdf:type owl:Class ;

owl:equivalentClass [ rdf:type owl:Restriction ;

owl:onProperty <http://www.co-ode.org/ontologies/uia/ont.owl#hasMeasurementRange> ;

owl:maxQualifiedCardinality "1"^^xsd:nonNegativeInteger ;

owl:onDataRange xsd:string

] ;

rdfs:subClassOf ssn:MeasurementProperty ;

dc:source """skos:exactMatch 'measuring interval/measurement range' [VIM 4.7]

http://www.bipm.org/utils/common/documents/jcgm/JCGM_200_2008.pdf""" ;

rdfs:comment "The set of values that the sensor can return as the result of an observation under the defined conditions with the defined measurement properties. (If no conditions are specified or the conditions do not specify a range for the observed qualities, the measurement range is to be taken as the condition for the observed qualities.)" ;

rdfs:isDefinedBy "http://purl.oclc.org/NET/ssnx/ssn" ;

rdfs:label "Measurement Range" ;

rdfs:seeAlso "http://www.w3.org/2005/Incubator/ssn/wiki/SSN_Sensor#MeasuringCapability" .

### http://purl.oclc.org/NET/ssnx/ssn#Observation

ssn:Observation rdf:type owl:Class ;

rdfs:subClassOf DUL:Situation ,

[ rdf:type owl:Restriction ;

owl:onProperty DUL:includesEvent ;

owl:someValuesFrom ssn:Stimulus

] ,

[ rdf:type owl:Restriction ;

owl:onProperty ssn:featureOfInterest ;

owl:allValuesFrom ssn:FeatureOfInterest

] ,

[ rdf:type owl:Restriction ;

owl:onProperty ssn:observationResult ;

owl:allValuesFrom ssn:SensorOutput

] ,

[ rdf:type owl:Restriction ;

owl:onProperty ssn:observedBy ;

owl:allValuesFrom ssn:Sensor

] ,

[ rdf:type owl:Restriction ;

owl:onProperty ssn:observedProperty ;

owl:allValuesFrom ssn:Property

] ,

[ rdf:type owl:Restriction ;

owl:onProperty ssn:sensingMethodUsed ;

owl:allValuesFrom ssn:Sensing

] ,

[ rdf:type owl:Restriction ;

owl:onProperty ssn:observationResultTime ;

owl:minCardinality "0"^^xsd:nonNegativeInteger

] ,

[ rdf:type owl:Restriction ;

owl:onProperty ssn:observationSamplingTime ;

owl:minCardinality "0"^^xsd:nonNegativeInteger

] ,

[ rdf:type owl:Restriction ;

owl:onProperty ssn:qualityOfObservation ;

owl:minCardinality "0"^^xsd:nonNegativeInteger

] ,

[ rdf:type owl:Restriction ;

owl:onProperty ssn:featureOfInterest ;

owl:qualifiedCardinality "1"^^xsd:nonNegativeInteger ;

owl:onClass ssn:FeatureOfInterest

] ,

[ rdf:type owl:Restriction ;

owl:onProperty ssn:observedBy ;

owl:qualifiedCardinality "1"^^xsd:nonNegativeInteger ;

owl:onClass ssn:Sensor

] ,

[ rdf:type owl:Restriction ;

owl:onProperty ssn:observedProperty ;

owl:qualifiedCardinality "1"^^xsd:nonNegativeInteger ;

owl:onClass ssn:Property

] ,

[ rdf:type owl:Restriction ;

owl:onProperty ssn:sensingMethodUsed ;

owl:qualifiedCardinality "1"^^xsd:nonNegativeInteger ;

owl:onClass ssn:Sensing

] ;

dc:source """skos:closeMatch 'observation' [O&M]

http://www.opengeospatial.org/standards/om

Observation in this ontology and O&M are described differently (O&M records an observation as an act/event), but they record the same thing and are essentially interchangeable. The difference is in the ontological structure of the two, not the data or use.

Observation here records a Situation (the estimation of the value of a Property) and a description of the method that was used (along with the participants), while O&M interprets an Observation as the event itself; there must, however, have been an event that lead to our situation, so both are records of events. The distinction is between the event itself and the record of what happened in that event.

skos:closeMatch 'measurement result' [VIM 2.9] http://www.bipm.org/utils/common/documents/jcgm/JCGM_200_2008.pdf

Measurement result in VIM is the measured value plus any other relevant information, which means that measurement result and observation will often be associated to the same data (a value, a time, a property, etc.).""" ;

rdfs:comment "An Observation is a Situation in which a Sensing method has been used to estimate or calculate a value of a Property of a FeatureOfInterest. Links to Sensing and Sensor describe what made the Observation and how; links to Property and Feature detail what was sensed; the result is the output of a Sensor; other metadata details times etc." ;

rdfs:isDefinedBy "http://purl.oclc.org/NET/ssnx/ssn" ;

rdfs:label "Observation" ;

rdfs:seeAlso "http://www.w3.org/2005/Incubator/ssn/wiki/SSN_Skeleton#Skeleton" .

### http://purl.oclc.org/NET/ssnx/ssn#ObservationValue

ssn:ObservationValue rdf:type owl:Class ;

rdfs:subClassOf DUL:ObservableEntity ,

[ rdf:type owl:Restriction ;

owl:onProperty <http://www.co-ode.org/ontologies/uia/ont.owl#hasTimeStamp> ;

owl:someValuesFrom <http://www.co-ode.org/ontologies/uia/ont.owl#TemporalEntity>

] ,

[ rdf:type owl:Restriction ;

owl:onProperty DUL:isRegionFor ;

owl:someValuesFrom ssn:SensorOutput

] ;

dc:source """skos:exactMatch 'measured quantity value' [VIM 2.10]

http://www.bipm.org/utils/common/documents/jcgm/JCGM_200_2008.pdf

skos:exactMatch 'observed value' [SensorML OGC-0700]

http://www.opengeospatial.org/standards/sensorml

skos:closeMatch 'observation result' [O&M]

http://www.opengeospatial.org/standards/om

O&M conflates what we have as SensorOutput and ObservationValue into observation result, though the OGC standard does say \"result contains a value\" and \"a result which has a value\", which fits naturally with the model here.""" ;

rdfs:comment "The value of the result of an Observation. An Observation has a result which is the output of some sensor, the result is an information object that encodes some value for a Feature." ;

rdfs:isDefinedBy "http://purl.oclc.org/NET/ssnx/ssn" ;

rdfs:label "Observation Value" ;

rdfs:seeAlso "http://www.w3.org/2005/Incubator/ssn/wiki/SSN_Base#Data" .

### http://purl.oclc.org/NET/ssnx/ssn#OperatingPowerRange

ssn:OperatingPowerRange rdf:type owl:Class ;

rdfs:subClassOf ssn:OperatingProperty ,

[ rdf:type owl:Restriction ;

owl:onProperty <http://www.co-ode.org/ontologies/uia/ont.owl#hasOperatingPowerRange> ;

owl:qualifiedCardinality "1"^^xsd:nonNegativeInteger ;

owl:onDataRange xsd:string

] ;

rdfs:comment "Power range in which system/sensor is expected to operate." ;

rdfs:isDefinedBy "http://purl.oclc.org/NET/ssnx/ssn" ;

rdfs:label "Operating Power Range" ;

rdfs:seeAlso "http://www.w3.org/2005/Incubator/ssn/wiki/SSN_Energy#EnergyRestriction" .

### http://purl.oclc.org/NET/ssnx/ssn#OperatingProperty

ssn:OperatingProperty rdf:type owl:Class ;

rdfs:subClassOf ssn:Property ;

rdfs:comment "An identifiable characteristic of the environmental and other conditions in which the sensor is intended to operate. May include power ranges, power sources, standard configurations, attachments and the like." ;

rdfs:isDefinedBy "http://purl.oclc.org/NET/ssnx/ssn" ;

rdfs:label "Operating Property" ;

rdfs:seeAlso "http://www.w3.org/2005/Incubator/ssn/wiki/SSN_Deploy#OperatingRestriction" .

### http://purl.oclc.org/NET/ssnx/ssn#OperatingRange

ssn:OperatingRange rdf:type owl:Class ;

rdfs:subClassOf ssn:Property ,

[ rdf:type owl:Restriction ;

owl:onProperty ssn:hasOperatingProperty ;

owl:allValuesFrom ssn:OperatingProperty

] ,

[ rdf:type owl:Restriction ;

owl:onProperty ssn:inCondition ;

owl:allValuesFrom ssn:Condition

] ;

dc:source """skos:broaderMatch 'reference operating condition' [VIM 4.11]

http://www.bipm.org/utils/common/documents/jcgm/JCGM_200_2008.pdf

The difference is that here we also allow for qualities that aren't VIM influence quantities [VIM 2.52] - for example, a quantity that alters the power requirements, but doesn't affect the measurement properties - conditions specified in MeasurementCapability should be influence quantities.""" ;

rdfs:comment "The environmental conditions and characteristics of a system/sensor's normal operating environment. Can be used to specify for example the standard environmental conditions in which the sensor is expected to operate (a Condition with no OperatingProperty), or how the environmental and other operating properties relate: i.e., that the maintenance schedule or power requirements differ according to the conditions." ;

rdfs:isDefinedBy "http://purl.oclc.org/NET/ssnx/ssn" ;

rdfs:label "Operating Range" ;

rdfs:seeAlso "http://www.w3.org/2005/Incubator/ssn/wiki/SSN_Deploy#OperatingRestriction" .

### http://purl.oclc.org/NET/ssnx/ssn#Platform

ssn:Platform rdf:type owl:Class ;

rdfs:subClassOf DUL:PhysicalObject ,

[ rdf:type owl:Restriction ;

owl:onProperty ssn:attachedSystem ;

owl:allValuesFrom ssn:System

] ,

[ rdf:type owl:Restriction ;

owl:onProperty ssn:inDeployment ;

owl:allValuesFrom ssn:Deployment

] ;

dc:source """skos:exactMatch 'platform' [SensorML OGC-0700]

http://www.opengeospatial.org/standards/sensorml""" ;

rdfs:comment "An Entity to which other Entities can be attached - particuarly Sensors and other Platforms. For example, a post might act as the Platform, a bouy might act as a Platform, or a fish might act as a Platform for an attached sensor." ;

rdfs:isDefinedBy "http://purl.oclc.org/NET/ssnx/ssn" ;

rdfs:label "Platform" ;

rdfs:seeAlso "http://www.w3.org/2005/Incubator/ssn/wiki/SSN_Deploy#PlatformSite" .

### http://purl.oclc.org/NET/ssnx/ssn#Precision

ssn:Precision rdf:type owl:Class ;

owl:equivalentClass [ rdf:type owl:Restriction ;

owl:onProperty <http://www.co-ode.org/ontologies/uia/ont.owl#hasPrecisionValue> ;

owl:maxQualifiedCardinality "1"^^xsd:nonNegativeInteger ;

owl:onDataRange xsd:string

] ;

rdfs:subClassOf ssn:MeasurementProperty ;

dc:source """skos:exactMatch 'measurement precision/precision' [VIM 2.15]

http://www.bipm.org/utils/common/documents/jcgm/JCGM_200_2008.pdf""" ;

rdfs:comment "The closeness of agreement between replicate observations on an unchanged or similar quality value: i.e., a measure of a sensor's ability to consitently reproduce an observation." ;

rdfs:isDefinedBy "http://purl.oclc.org/NET/ssnx/ssn" ;

rdfs:label "Precision" ;

rdfs:seeAlso "http://www.w3.org/2005/Incubator/ssn/wiki/SSN_Sensor#MeasuringCapability" .

### http://purl.oclc.org/NET/ssnx/ssn#Process

ssn:Process rdf:type owl:Class ;

rdfs:subClassOf DUL:Method ;

dc:source "http://www.w3.org/2005/Incubator/ssn/" ;

rdfs:comment "A process has an output and possibly inputs and, for a composite process, describes the temporal and dataflow dependencies and relationships amongst its parts. [SSN XG]" ;

rdfs:isDefinedBy "http://purl.oclc.org/NET/ssnx/ssn" ;

rdfs:label "Process" ;

rdfs:seeAlso "http://www.w3.org/2005/Incubator/ssn/wiki/SSN_Model#Process" .

### http://purl.oclc.org/NET/ssnx/ssn#Property

ssn:Property rdf:type owl:Class ;

rdfs:subClassOf DUL:Quality ,

[ rdf:type owl:Restriction ;

owl:onProperty ssn:isPropertyOf ;

owl:someValuesFrom ssn:FeatureOfInterest

] ;

dc:source """skos:exactMatch 'property' [O&M]

http://www.opengeospatial.org/standards/om""" ;

rdfs:comment "An observable Quality of an Event or Object. That is, not a quality of an abstract entity as is also allowed by DUL's Quality, but rather an aspect of an entity that is intrinsic to and cannot exist without the entity and is observable by a sensor." ;

rdfs:isDefinedBy "http://purl.oclc.org/NET/ssnx/ssn" ;

rdfs:label "Property" ;

rdfs:seeAlso "http://www.w3.org/2005/Incubator/ssn/wiki/SSN_Skeleton#Skeleton" .

### http://purl.oclc.org/NET/ssnx/ssn#Resolution

ssn:Resolution rdf:type owl:Class ;

owl:equivalentClass [ rdf:type owl:Restriction ;

owl:onProperty <http://www.co-ode.org/ontologies/uia/ont.owl#hasResolution> ;

owl:maxQualifiedCardinality "1"^^xsd:nonNegativeInteger ;

owl:onDataRange xsd:string

] ;

rdfs:subClassOf ssn:MeasurementProperty ;

dc:source """skos:exactMatch 'resolution' [VIM 4.14]

http://www.bipm.org/utils/common/documents/jcgm/JCGM_200_2008.pdf""" ;

rdfs:comment "The smallest difference in the value of a quality being observed that would result in perceptably different values of observation results." ;

rdfs:isDefinedBy "http://purl.oclc.org/NET/ssnx/ssn" ;

rdfs:label "Resolution" ;

rdfs:seeAlso "http://www.w3.org/2005/Incubator/ssn/wiki/SSN_Sensor#MeasuringCapability" .

### http://purl.oclc.org/NET/ssnx/ssn#ResponseTime

ssn:ResponseTime rdf:type owl:Class ;

owl:equivalentClass [ rdf:type owl:Restriction ;

owl:onProperty <http://www.co-ode.org/ontologies/uia/ont.owl#hasResponseTime> ;

owl:maxQualifiedCardinality "1"^^xsd:nonNegativeInteger ;

owl:onDataRange xsd:string

] ;

rdfs:subClassOf ssn:MeasurementProperty ;

dc:source """skos:exactMatch 'step response time' [VIM 4.23]

http://www.bipm.org/utils/common/documents/jcgm/JCGM_200_2008.pdf""" ;

rdfs:comment "The time between a (step) change inthe value of an observed quality and a sensor (possibly with specified error) 'settling' on an observed value." ;

rdfs:isDefinedBy "http://purl.oclc.org/NET/ssnx/ssn" ;

rdfs:label "Response time" ;

rdfs:seeAlso "http://www.w3.org/2005/Incubator/ssn/wiki/SSN_Sensor#MeasuringCapability" .

### http://purl.oclc.org/NET/ssnx/ssn#Selectivity

ssn:Selectivity rdf:type owl:Class ;

owl:equivalentClass [ rdf:type owl:Restriction ;

owl:onProperty <http://www.co-ode.org/ontologies/uia/ont.owl#hasSelectivity> ;

owl:maxQualifiedCardinality "1"^^xsd:nonNegativeInteger ;

owl:onDataRange xsd:string

] ;

rdfs:subClassOf ssn:MeasurementProperty ;

dc:source """skos:exactMatch 'selectivity' [VIM 4.13]

http://www.bipm.org/utils/common/documents/jcgm/JCGM_200_2008.pdf""" ;

rdfs:comment "Selectivity is a property of a sensor whereby it provides observed values for one or more qualities such that the values of each quality are independent of other qualities in the phenomenon, body, or substance being investigated." ;

rdfs:isDefinedBy "http://purl.oclc.org/NET/ssnx/ssn" ;

rdfs:label "Selectivity" ;

rdfs:seeAlso "http://www.w3.org/2005/Incubator/ssn/wiki/SSN_Sensor#MeasuringCapability" .

### http://purl.oclc.org/NET/ssnx/ssn#Sensing

ssn:Sensing rdf:type owl:Class ;

rdfs:subClassOf ssn:Process ;

dc:source "http://www.w3.org/2005/Incubator/ssn/" ;

rdfs:comment "Sensing is a process that results in the estimation, or calculation, of the value of a phenomenon." ;

rdfs:isDefinedBy "http://purl.oclc.org/NET/ssnx/ssn" ;

rdfs:label "Sensing" ;

rdfs:seeAlso "http://www.w3.org/2005/Incubator/ssn/wiki/SSN_Skeleton#Skeleton" .

### http://purl.oclc.org/NET/ssnx/ssn#Sensitivity

ssn:Sensitivity rdf:type owl:Class ;

owl:equivalentClass [ rdf:type owl:Restriction ;

owl:onProperty <http://www.co-ode.org/ontologies/uia/ont.owl#hasSensitivity> ;

owl:maxQualifiedCardinality "1"^^xsd:nonNegativeInteger ;

owl:onDataRange xsd:string

] ;

rdfs:subClassOf ssn:MeasurementProperty ;

dc:source """skos:exactMatch 'sensitivity' [VIM 4.12]

http://www.bipm.org/utils/common/documents/jcgm/JCGM_200_2008.pdf""" ;

rdfs:comment "Sensitivity is the quotient of the change in a result of sensor and the corresponding change in a value of a quality being observed." ;

rdfs:isDefinedBy "http://purl.oclc.org/NET/ssnx/ssn" ;

rdfs:label "Sensitivity" ;

rdfs:seeAlso "http://www.w3.org/2005/Incubator/ssn/wiki/SSN_Sensor#MeasuringCapability" .

### http://purl.oclc.org/NET/ssnx/ssn#Sensor

ssn:Sensor rdf:type owl:Class ;

rdfs:subClassOf DUL:PhysicalObject ,

[ rdf:type owl:Restriction ;

owl:onProperty ssn:implements ;

owl:someValuesFrom ssn:Sensing

] ,

[ rdf:type owl:Restriction ;

owl:onProperty <http://www.co-ode.org/ontologies/uia/ont.owl#hasOutput> ;

owl:someValuesFrom ssn:SensorOutput

] ,

[ rdf:type owl:Restriction ;

owl:onProperty ssn:detects ;

owl:allValuesFrom ssn:Stimulus

] ,

[ rdf:type owl:Restriction ;

owl:onProperty ssn:hasMeasurementCapability ;

owl:allValuesFrom ssn:MeasurementCapability

] ,

[ rdf:type owl:Restriction ;

owl:onProperty ssn:observes ;

owl:allValuesFrom ssn:Property

] ;

dc:source """skos:exactMatch 'sensor' [SensorML OGC-0700]

http://www.opengeospatial.org/standards/sensorml

skos:closeMatch 'observation procedure' [O&M]

http://www.opengeospatial.org/standards/om

O&M allows sensors, methods, instruments, systems, algorithms and process chains as the processUsed of an observation; this ontology allows a similar range of things (any thing that can do sensing), just they are all grouped under the term sensor (which is thus wider than the O&M concept).""" ;

rdfs:comment "A sensor can do (implements) sensing: that is, a sensor is any entity that can follow a sensing method and thus observe some Property of a FeatureOfInterest. Sensors may be physical devices, computational methods, a laboratory setup with a person following a method, or any other thing that can follow a Sensing Method to observe a Property." ;

rdfs:isDefinedBy "http://purl.oclc.org/NET/ssnx/ssn" ;

rdfs:label "Sensor" ;

rdfs:seeAlso "http://www.w3.org/2005/Incubator/ssn/wiki/SSN_Skeleton#Skeleton" .

### http://purl.oclc.org/NET/ssnx/ssn#SensorDataSheet

ssn:SensorDataSheet rdf:type owl:Class ;

rdfs:subClassOf DUL:InformationObject ;

dc:source "http://www.w3.org/2005/Incubator/ssn/" ;

rdfs:comment """A data sheet records properties of a sensor. A data sheet might describe for example the accuracy in various conditions, the power use, the types of connectors that the sensor has, etc.

Generally a sensor's properties are recorded directly (with hasMeasurementCapability, for example), but the data sheet can be used for example to record the manufacturers specifications verses observed capabilites, or if more is known than the manufacturer specifies, etc. The data sheet is an information object about the sensor's properties, rather than a direct link to the actual properties themselves.""" ;

rdfs:isDefinedBy "http://purl.oclc.org/NET/ssnx/ssn" ;

rdfs:label "Sensor Data Sheet" ;

rdfs:seeAlso "http://www.w3.org/2005/Incubator/ssn/wiki/SSN_Sensor#Measuring" .

### http://purl.oclc.org/NET/ssnx/ssn#SensorInput

ssn:SensorInput rdf:type owl:Class ;

rdfs:subClassOf DUL:Event ,

[ rdf:type owl:Restriction ;

owl:onProperty ssn:isProxyFor ;

owl:allValuesFrom ssn:Property

] ;

dc:source "http://www.w3.org/2005/Incubator/ssn/" ;

rdfs:comment "An Event in the real world that 'triggers' the sensor. The properties associated to the stimulus may be different to eventual observed property. It is the event, not the object that triggers the sensor." ;

rdfs:isDefinedBy "http://purl.oclc.org/NET/ssnx/ssn" ;

rdfs:label "Sensor Input" ;

rdfs:seeAlso "http://www.w3.org/2005/Incubator/ssn/wiki/SSN_Skeleton#Skeleton" .

### http://purl.oclc.org/NET/ssnx/ssn#SensorOutput

ssn:SensorOutput rdf:type owl:Class ;

rdfs:subClassOf DUL:Event ,

[ rdf:type owl:Restriction ;

owl:onProperty ssn:hasValue ;

owl:someValuesFrom ssn:ObservationValue

] ,

[ rdf:type owl:Restriction ;

owl:onProperty ssn:isProducedBy ;

owl:someValuesFrom ssn:Sensor

] ;

dc:source """http://www.w3.org/2005/Incubator/ssn/

skos:closeMatch 'observation result' [O&M]

http://www.opengeospatial.org/standards/om

See comments at ObservationValue.""" ;

rdfs:comment "A sensor outputs a piece of information (an observed value), the value itself being represented by an ObservationValue." ;

rdfs:isDefinedBy "http://purl.oclc.org/NET/ssnx/ssn" ;

rdfs:label "Sensor Output" ;

rdfs:seeAlso "http://www.w3.org/2005/Incubator/ssn/wiki/SSN_Skeleton#Skeleton" .

### http://purl.oclc.org/NET/ssnx/ssn#Stimulus

ssn:Stimulus rdf:type owl:Class ;

rdfs:subClassOf DUL:Event ;

dc:source "http://www.w3.org/2005/Incubator/ssn/" ;

rdfs:comment "An Event in the real world that 'triggers' the sensor. The properties associated to the stimulus may be different to eventual observed property. It is the event, not the object that triggers the sensor." ;

rdfs:isDefinedBy "http://purl.oclc.org/NET/ssnx/ssn" ;

rdfs:label "Stimulus" ;

rdfs:seeAlso "http://www.w3.org/2005/Incubator/ssn/wiki/SSN_Skeleton#Skeleton" .

### http://purl.oclc.org/NET/ssnx/ssn#SurvivalProperty

ssn:SurvivalProperty rdf:type owl:Class ;

rdfs:subClassOf ssn:Property ,

[ rdf:type owl:Restriction ;

owl:onProperty <http://www.co-ode.org/ontologies/uia/ont.owl#hasTimeStamp> ;

owl:someValuesFrom <http://www.co-ode.org/ontologies/uia/ont.owl#TemporalEntity>

] ;

rdfs:comment "An identifiable characteristic that represents the extent of the sensors useful life. Might include for example total battery life or number of recharges, or, for sensors that are used only a fixed number of times, the number of observations that can be made before the sensing capability is depleted." ;

rdfs:isDefinedBy "http://purl.oclc.org/NET/ssnx/ssn" ;

rdfs:label "Survival Property" ;

rdfs:seeAlso "http://www.w3.org/2005/Incubator/ssn/wiki/SSN_Deploy#OperatingRestriction" .

### http://purl.oclc.org/NET/ssnx/ssn#SurvivalRange

ssn:SurvivalRange rdf:type owl:Class ;

rdfs:subClassOf ssn:Property ,

[ rdf:type owl:Restriction ;

owl:onProperty ssn:hasSurvivalProperty ;

owl:allValuesFrom ssn:SurvivalProperty

] ,

[ rdf:type owl:Restriction ;

owl:onProperty ssn:inCondition ;

owl:allValuesFrom ssn:Condition

] ;

dc:source """skos:narrowerMatch 'limiting operating condition' [VIM 4.10]

http://www.bipm.org/utils/common/documents/jcgm/JCGM_200_2008.pdf""" ;

rdfs:comment "The conditions a sensor can be exposed to without damage: i.e., the sensor continues to operate as defined using MeasurementCapability. If, however, the SurvivalRange is exceeded, the sensor is 'damaged' and MeasurementCapability specifications may no longer hold." ;

rdfs:isDefinedBy "http://purl.oclc.org/NET/ssnx/ssn" ;

rdfs:label "Survival Range" ;

rdfs:seeAlso "http://www.w3.org/2005/Incubator/ssn/wiki/SSN_Deploy#OperatingRestriction" .

### http://purl.oclc.org/NET/ssnx/ssn#System

ssn:System rdf:type owl:Class ;

rdfs:subClassOf DUL:PhysicalObject ,

[ rdf:type owl:Restriction ;

owl:onProperty ssn:hasSubSystem ;

owl:someValuesFrom ssn:System

] ,

[ rdf:type owl:Restriction ;

owl:onProperty ssn:hasDeployment ;

owl:allValuesFrom ssn:Deployment

] ,

[ rdf:type owl:Restriction ;

owl:onProperty ssn:hasOperatingRange ;

owl:allValuesFrom ssn:OperatingRange

] ,

[ rdf:type owl:Restriction ;

owl:onProperty ssn:hasSubSystem ;

owl:allValuesFrom ssn:System

] ,

[ rdf:type owl:Restriction ;

owl:onProperty ssn:hasSurvivalRange ;

owl:allValuesFrom ssn:SurvivalRange

] ,

[ rdf:type owl:Restriction ;

owl:onProperty ssn:onPlatform ;

owl:allValuesFrom ssn:Platform

] ;

dc:source "http://www.w3.org/2005/Incubator/ssn/" ;

rdfs:comment "System is a unit of abstraction for pieces of infrastructure (and we largely care that they are) for sensing. A system has components, its subsystems, which are other systems." ;

rdfs:isDefinedBy "http://purl.oclc.org/NET/ssnx/ssn" ;

rdfs:label "System" ;

rdfs:seeAlso "http://www.w3.org/2005/Incubator/ssn/wiki/SSN_Model#System" .

### http://purl.oclc.org/NET/ssnx/ssn#SystemLifetime

ssn:SystemLifetime rdf:type owl:Class ;

rdfs:subClassOf ssn:SurvivalProperty ;

rdfs:comment "Total useful life of a sensor/system (expressed as total life since manufacture, time in use, number of operations, etc.)." ;

rdfs:isDefinedBy "http://purl.oclc.org/NET/ssnx/ssn" ;

rdfs:label "System Lifetime" ;

rdfs:seeAlso "http://www.w3.org/2005/Incubator/ssn/wiki/SSN_Deploy#OperatingRestriction" .

### http://www.co-ode.org/ontologies/uia/ont.owl#Activity

<http://www.co-ode.org/ontologies/uia/ont.owl#Activity> rdf:type owl:Class ;

owl:equivalentClass [ rdf:type owl:Restriction ;

owl:onProperty <http://www.co-ode.org/ontologies/uia/ont.owl#hasSCTID> ;

owl:hasValue "257733005"^^xsd:long

] ;

rdfs:subClassOf <http://www.co-ode.org/ontologies/uia/ont.owl#Process> ,

[ rdf:type owl:Restriction ;

owl:onProperty ssn:isFoundBy ;

owl:qualifiedCardinality "1"^^xsd:nonNegativeInteger ;

owl:onClass <http://www.co-ode.org/ontologies/uia/ont.owl#Fit_for_activity_finding>

] ;

rdfs:comment """Activity (observable entity)

SCTID: 257733005

257733005 | Activity (observable entity) |

en Activity (observable entity)

en Activities

en Activity""" .

### http://www.co-ode.org/ontologies/uia/ont.owl#ActivityData

<http://www.co-ode.org/ontologies/uia/ont.owl#ActivityData> rdf:type owl:Class ;

rdfs:subClassOf <http://www.co-ode.org/ontologies/uia/ont.owl#ParticipantHealthRecord> ,

[ rdf:type owl:Restriction ;

owl:onProperty <http://www.co-ode.org/ontologies/uia/ont.owl#hasBeenCollectedBy> ;

owl:someValuesFrom ssn:DailyActivityData

] ,

[ rdf:type owl:Restriction ;

owl:onProperty <http://www.co-ode.org/ontologies/uia/ont.owl#hasBeenCollectedBy> ;

owl:someValuesFrom <http://www.co-ode.org/ontologies/uia/ont.owl#ActivityDataValue>

] .

### http://www.co-ode.org/ontologies/uia/ont.owl#ActivityDataValue

<http://www.co-ode.org/ontologies/uia/ont.owl#ActivityDataValue> rdf:type owl:Class ;

rdfs:subClassOf ssn:ObservationValue ,

[ rdf:type owl:Restriction ;

owl:onProperty <http://www.co-ode.org/ontologies/uia/ont.owl#hasActivityBouts> ;

owl:qualifiedCardinality "1"^^xsd:nonNegativeInteger ;

owl:onDataRange xsd:integer

] ,

[ rdf:type owl:Restriction ;

owl:onProperty <http://www.co-ode.org/ontologies/uia/ont.owl#hasCurrentHeartRate> ;

owl:qualifiedCardinality "1"^^xsd:nonNegativeInteger ;

owl:onDataRange xsd:integer

] ,

[ rdf:type owl:Restriction ;

owl:onProperty <http://www.co-ode.org/ontologies/uia/ont.owl#hasDistanceCovered> ;

owl:qualifiedCardinality "1"^^xsd:nonNegativeInteger ;

owl:onDataRange xsd:integer

] ,

[ rdf:type owl:Restriction ;

owl:onProperty <http://www.co-ode.org/ontologies/uia/ont.owl#hasMetabolicRate> ;

owl:qualifiedCardinality "1"^^xsd:nonNegativeInteger ;

owl:onDataRange xsd:integer

] ,

[ rdf:type owl:Restriction ;

owl:onProperty <http://www.co-ode.org/ontologies/uia/ont.owl#hasPhysicalActivityType> ;

owl:qualifiedCardinality "1"^^xsd:nonNegativeInteger ;

owl:onDataRange xsd:string

] ,

[ rdf:type owl:Restriction ;

owl:onProperty <http://www.co-ode.org/ontologies/uia/ont.owl#hasSedentaryBouts> ;

owl:qualifiedCardinality "1"^^xsd:nonNegativeInteger ;

owl:onDataRange xsd:integer

] ,

[ rdf:type owl:Restriction ;

owl:onProperty <http://www.co-ode.org/ontologies/uia/ont.owl#hasSteps> ;

owl:qualifiedCardinality "1"^^xsd:nonNegativeInteger ;

owl:onDataRange xsd:integer

] ,

[ rdf:type owl:Restriction ;

owl:onProperty <http://www.co-ode.org/ontologies/uia/ont.owl#hasTotalSleepTime> ;

owl:qualifiedCardinality "1"^^xsd:nonNegativeInteger ;

owl:onDataRange xsd:integer

] .

### http://www.co-ode.org/ontologies/uia/ont.owl#ActivityDevice

<http://www.co-ode.org/ontologies/uia/ont.owl#ActivityDevice> rdf:type owl:Class ;

rdfs:subClassOf ssn:Sensor .

### http://www.co-ode.org/ontologies/uia/ont.owl#ActivityRecommendation

<http://www.co-ode.org/ontologies/uia/ont.owl#ActivityRecommendation> rdf:type owl:Class ;

rdfs:subClassOf <http://www.co-ode.org/ontologies/uia/ont.owl#Recommendation> .

### http://www.co-ode.org/ontologies/uia/ont.owl#Admin

<http://www.co-ode.org/ontologies/uia/ont.owl#Admin> rdf:type owl:Class ;

rdfs:subClassOf <http://www.co-ode.org/ontologies/uia/ont.owl#Human> ,

[ rdf:type owl:Restriction ;

owl:onProperty <http://www.co-ode.org/ontologies/uia/ont.owl#hasOfficeAddress> ;

owl:someValuesFrom <http://www.co-ode.org/ontologies/uia/ont.owl#OfficeAddress>

] ,

[ rdf:type owl:Restriction ;

owl:onProperty <http://www.co-ode.org/ontologies/uia/ont.owl#hasPersonalData> ;

owl:someValuesFrom <http://www.co-ode.org/ontologies/uia/ont.owl#PersonalData>

] .

### http://www.co-ode.org/ontologies/uia/ont.owl#Alcohol_consumption_unknown

<http://www.co-ode.org/ontologies/uia/ont.owl#Alcohol_consumption_unknown> rdf:type owl:Class ;

owl:equivalentClass [ rdf:type owl:Restriction ;

owl:onProperty <http://www.co-ode.org/ontologies/uia/ont.owl#hasSCTID> ;

owl:hasValue "160580001"^^xsd:long

] ;

rdfs:subClassOf <http://www.co-ode.org/ontologies/uia/ont.owl#Finding_of_alcohol_intake> ;

rdfs:comment """Alcohol consumption unknown (finding)

SCTID: 160580001

160580001 | Alcohol consumption unknown (finding) |

en Alcohol consumption unknown

en Alcohol consumption unknown (finding)""" .

### http://www.co-ode.org/ontologies/uia/ont.owl#Balanced_diet

<http://www.co-ode.org/ontologies/uia/ont.owl#Balanced_diet> rdf:type owl:Class ;

owl:equivalentClass [ rdf:type owl:Restriction ;

owl:onProperty <http://www.co-ode.org/ontologies/uia/ont.owl#hasSCTID> ;

owl:hasValue "226229006"^^xsd:long

] ;

rdfs:subClassOf <http://www.co-ode.org/ontologies/uia/ont.owl#Dietary_finding> ;

rdfs:comment """Balanced diet (finding)

SCTID: 226229006

226229006 | Balanced diet (finding) |

en Balanced diet

en Balanced diet (finding)""" .

### http://www.co-ode.org/ontologies/uia/ont.owl#BaselineData

<http://www.co-ode.org/ontologies/uia/ont.owl#BaselineData> rdf:type owl:Class ;

rdfs:subClassOf <http://www.co-ode.org/ontologies/uia/ont.owl#ParticipantHealthRecord> ,

[ rdf:type owl:Restriction ;

owl:onProperty <http://www.co-ode.org/ontologies/uia/ont.owl#hasBeenCollectedBy> ;

owl:someValuesFrom <http://www.co-ode.org/ontologies/uia/ont.owl#Interview>

] ,

[ rdf:type owl:Restriction ;

owl:onProperty ssn:hasFastingBloodGlucose ;

owl:qualifiedCardinality "1"^^xsd:nonNegativeInteger ;

owl:onDataRange xsd:double

] ,

[ rdf:type owl:Restriction ;

owl:onProperty ssn:hasInitialBMI ;

owl:qualifiedCardinality "1"^^xsd:nonNegativeInteger ;

owl:onDataRange xsd:double

] ,

[ rdf:type owl:Restriction ;

owl:onProperty ssn:hasInitialWeight ;

owl:qualifiedCardinality "1"^^xsd:nonNegativeInteger ;

owl:onDataRange xsd:double

] ,

[ rdf:type owl:Restriction ;

owl:onProperty ssn:hasWaistHipRatio ;

owl:qualifiedCardinality "1"^^xsd:nonNegativeInteger ;

owl:onDataRange xsd:double

] ,

[ rdf:type owl:Restriction ;

owl:onProperty <http://www.co-ode.org/ontologies/uia/ont.owl#hasCurrentDiastolicBP> ;

owl:qualifiedCardinality "1"^^xsd:nonNegativeInteger ;

owl:onDataRange xsd:integer

] ,

[ rdf:type owl:Restriction ;

owl:onProperty <http://www.co-ode.org/ontologies/uia/ont.owl#hasCurrentHDLLevel> ;

owl:qualifiedCardinality "1"^^xsd:nonNegativeInteger ;

owl:onDataRange xsd:integer

] ,

[ rdf:type owl:Restriction ;

owl:onProperty <http://www.co-ode.org/ontologies/uia/ont.owl#hasCurrentLDLLevel> ;

owl:qualifiedCardinality "1"^^xsd:nonNegativeInteger ;

owl:onDataRange xsd:integer

] ,

[ rdf:type owl:Restriction ;

owl:onProperty <http://www.co-ode.org/ontologies/uia/ont.owl#hasCurrentSystolicBP> ;

owl:qualifiedCardinality "1"^^xsd:nonNegativeInteger ;

owl:onDataRange xsd:integer

] ,

[ rdf:type owl:Restriction ;

owl:onProperty <http://www.co-ode.org/ontologies/uia/ont.owl#hasHeight> ;

owl:qualifiedCardinality "1"^^xsd:nonNegativeInteger ;

owl:onDataRange xsd:integer

] .

### http://www.co-ode.org/ontologies/uia/ont.owl#Baseline_weight

<http://www.co-ode.org/ontologies/uia/ont.owl#Baseline_weight> rdf:type owl:Class ;

owl:equivalentClass [ rdf:type owl:Restriction ;

owl:onProperty <http://www.co-ode.org/ontologies/uia/ont.owl#hasSCTID> ;

owl:hasValue "400967004"^^xsd:long

] ;

rdfs:subClassOf <http://www.co-ode.org/ontologies/uia/ont.owl#Reference_weight> ;

rdfs:comment """Baseline weight (observable entity)

SCTID: 400967004

400967004 | Baseline weight (observable entity) |

en Baseline weight

en Baseline weight (observable entity)""" .

### http://www.co-ode.org/ontologies/uia/ont.owl#Behavior_observable

<http://www.co-ode.org/ontologies/uia/ont.owl#Behavior_observable> rdf:type owl:Class ;

owl:equivalentClass [ rdf:type owl:Restriction ;

owl:onProperty <http://www.co-ode.org/ontologies/uia/ont.owl#hasSCTID> ;

owl:hasValue "363896009"^^xsd:long

] ;

rdfs:subClassOf <http://www.co-ode.org/ontologies/uia/ont.owl#Mental_state_behavior_psychosocial_function_observable> ;

rdfs:comment """Behavior observable (observable entity)

SCTID: 363896009

363896009 | Behavior observable (observable entity) |

en Behavior observable (observable entity)

en Behaviour observable

en Behavior observable""" .

### http://www.co-ode.org/ontologies/uia/ont.owl#Blood_glucose_status

<http://www.co-ode.org/ontologies/uia/ont.owl#Blood_glucose_status> rdf:type owl:Class ;

owl:equivalentClass [ rdf:type owl:Restriction ;

owl:onProperty <http://www.co-ode.org/ontologies/uia/ont.owl#hasSCTID> ;

owl:hasValue "405176005"^^xsd:long

] ;

rdfs:subClassOf <http://www.co-ode.org/ontologies/uia/ont.owl#General_clinical_state> ,

[ rdf:type owl:Restriction ;

owl:onProperty ssn:isFoundBy ;

owl:qualifiedCardinality "1"^^xsd:nonNegativeInteger ;

owl:onClass <http://www.co-ode.org/ontologies/uia/ont.owl#Finding_of_glucose_level>

] ;

rdfs:comment """Blood glucose status (observable entity)

SCTID: 405176005

405176005 | Blood glucose status (observable entity) |

en Blood glucose status

en Blood glucose status (observable entity)""" .

### http://www.co-ode.org/ontologies/uia/ont.owl#Blood_pressure

<http://www.co-ode.org/ontologies/uia/ont.owl#Blood_pressure> rdf:type owl:Class ;

owl:equivalentClass [ rdf:type owl:Restriction ;

owl:onProperty <http://www.co-ode.org/ontologies/uia/ont.owl#hasSCTID> ;

owl:hasValue "75367002"^^xsd:long

] ;

rdfs:subClassOf <http://www.co-ode.org/ontologies/uia/ont.owl#Cardiovascular_measure> ;

rdfs:comment """Blood pressure (observable entity)

SCTID: 75367002

75367002 | Blood pressure (observable entity) |

en Blood pressure

en Blood pressure (observable entity)

en BP - Blood pressure""" .

### http://www.co-ode.org/ontologies/uia/ont.owl#Blood_pressure_finding

<http://www.co-ode.org/ontologies/uia/ont.owl#Blood_pressure_finding> rdf:type owl:Class ;

owl:equivalentClass [ rdf:type owl:Restriction ;

owl:onProperty <http://www.co-ode.org/ontologies/uia/ont.owl#hasSCTID> ;

owl:hasValue "392570002"^^xsd:long

] ;

rdfs:subClassOf <http://www.co-ode.org/ontologies/uia/ont.owl#Vital_sign_finding> ;

rdfs:comment """Blood pressure finding (finding)

SCTID: 392570002

392570002 | Blood pressure finding (finding) |

en Blood pressure finding (finding)

en Blood pressure finding""" .

### http://www.co-ode.org/ontologies/uia/ont.owl#Body_height_measure

<http://www.co-ode.org/ontologies/uia/ont.owl#Body_height_measure> rdf:type owl:Class ;

owl:equivalentClass [ rdf:type owl:Restriction ;

owl:onProperty <http://www.co-ode.org/ontologies/uia/ont.owl#hasSCTID> ;

owl:hasValue "50373000"^^xsd:long

] ;

rdfs:subClassOf <http://www.co-ode.org/ontologies/uia/ont.owl#Height_growth_measure> ;

rdfs:comment """Body height measure (observable entity)

SCTID: 50373000

50373000 | Body height measure (observable entity) |

en Body height measure

en Body height

en Body height measure (observable entity)""" .

### http://www.co-ode.org/ontologies/uia/ont.owl#Body_mass_index

<http://www.co-ode.org/ontologies/uia/ont.owl#Body_mass_index> rdf:type owl:Class ;

owl:equivalentClass [ rdf:type owl:Restriction ;

owl:onProperty <http://www.co-ode.org/ontologies/uia/ont.owl#hasSCTID> ;

owl:hasValue "60621009"^^xsd:long

] ;

rdfs:subClassOf <http://www.co-ode.org/ontologies/uia/ont.owl#Nutritional_status> ,

[ rdf:type owl:Restriction ;

owl:onProperty ssn:isFoundBy ;

owl:qualifiedCardinality "1"^^xsd:nonNegativeInteger ;

owl:onClass <http://www.co-ode.org/ontologies/uia/ont.owl#Weight_finding>

] ;

rdfs:comment """Body mass index (observable entity)

SCTID: 60621009

60621009 | Body mass index (observable entity) |

en Body mass index

en BMI - Body mass index

en Body mass index (observable entity)

en Weight: body mass

en Quetelet index""" .

### http://www.co-ode.org/ontologies/uia/ont.owl#Body_mass_index_25-29_overweight_finding

<http://www.co-ode.org/ontologies/uia/ont.owl#Body_mass_index_25-29_overweight_finding> rdf:type owl:Class ;

owl:equivalentClass [ rdf:type owl:Restriction ;

owl:onProperty <http://www.co-ode.org/ontologies/uia/ont.owl#hasSCTID> ;

owl:hasValue "162863004"^^xsd:long

] ;

rdfs:subClassOf <http://www.co-ode.org/ontologies/uia/ont.owl#Overweight_finding> ;

rdfs:comment """Body mass index 25-29 - overweight (finding)

SCTID: 162863004

162863004 | Body mass index 25-29 - overweight (finding) |

en Body mass index 25-29 - overweight (finding)

en Body mass index 25-29 - overweight

en BMI 25-29 - overweight""" .

### http://www.co-ode.org/ontologies/uia/ont.owl#Body_measure

<http://www.co-ode.org/ontologies/uia/ont.owl#Body_measure> rdf:type owl:Class ;

owl:equivalentClass [ rdf:type owl:Restriction ;

owl:onProperty <http://www.co-ode.org/ontologies/uia/ont.owl#hasSCTID> ;

owl:hasValue "248326004"^^xsd:long

] ;

rdfs:subClassOf <http://www.co-ode.org/ontologies/uia/ont.owl#General_characteristic_of_patient> ,

[ rdf:type owl:Restriction ;

owl:onProperty ssn:isFoundBy ;

owl:qualifiedCardinality "1"^^xsd:nonNegativeInteger ;

owl:onClass <http://www.co-ode.org/ontologies/uia/ont.owl#Weight_finding>

] ;

rdfs:comment """Body measure (observable entity)

SCTID: 248326004

248326004 | Body measure (observable entity) |

en Body measure (observable entity)

en Body measure

en Body measurements""" .

### http://www.co-ode.org/ontologies/uia/ont.owl#Body_measurement_finding

<http://www.co-ode.org/ontologies/uia/ont.owl#Body_measurement_finding> rdf:type owl:Class ;

owl:equivalentClass [ rdf:type owl:Restriction ;

owl:onProperty <http://www.co-ode.org/ontologies/uia/ont.owl#hasSCTID> ;

owl:hasValue "365605003"^^xsd:long

] ;

rdfs:subClassOf <http://www.co-ode.org/ontologies/uia/ont.owl#General_finding_of_observation_of_patient> ;

rdfs:comment """Body measurement finding (finding)

SCTID: 365605003

365605003 | Body measurement finding (finding) |

en Body measurement finding (finding)

en Body measurement finding""" .

### http://www.co-ode.org/ontologies/uia/ont.owl#Body_weight

<http://www.co-ode.org/ontologies/uia/ont.owl#Body_weight> rdf:type owl:Class ;

owl:equivalentClass [ rdf:type owl:Restriction ;

owl:onProperty <http://www.co-ode.org/ontologies/uia/ont.owl#hasSCTID> ;

owl:hasValue "27113001"^^xsd:long

] ;

rdfs:subClassOf <http://www.co-ode.org/ontologies/uia/ont.owl#Measured_body_weight> ;

rdfs:comment """Body weight (observable entity)

SCTID: 27113001

27113001 | Body weight (observable entity) |

en Body weight

en Body weight (observable entity)""" .

### http://www.co-ode.org/ontologies/uia/ont.owl#Body_weight_characteristic

<http://www.co-ode.org/ontologies/uia/ont.owl#Body_weight_characteristic> rdf:type owl:Class ;

owl:equivalentClass [ rdf:type owl:Restriction ;

owl:onProperty <http://www.co-ode.org/ontologies/uia/ont.owl#hasSCTID> ;

owl:hasValue "363804004"^^xsd:long

] ;

rdfs:subClassOf <http://www.co-ode.org/ontologies/uia/ont.owl#Body_measure> ;

rdfs:comment """Body weight characteristic (observable entity)

SCTID: 363804004

363804004 | Body weight characteristic (observable entity) |

en Body weight characteristic (observable entity)

en Body weight characteristic""" .

### http://www.co-ode.org/ontologies/uia/ont.owl#Cardiovascular_function

<http://www.co-ode.org/ontologies/uia/ont.owl#Cardiovascular_function> rdf:type owl:Class ;

owl:equivalentClass [ rdf:type owl:Restriction ;

owl:onProperty <http://www.co-ode.org/ontologies/uia/ont.owl#hasSCTID> ;

owl:hasValue "70337006"^^xsd:long

] ;

rdfs:subClassOf <http://www.co-ode.org/ontologies/uia/ont.owl#Function> ;

rdfs:comment """Cardiovascular function (observable entity)

SCTID: 70337006

70337006 | Cardiovascular function (observable entity) |

en Cardiovascular function

en Cardiovascular function (observable entity)""" .

### http://www.co-ode.org/ontologies/uia/ont.owl#Cardiovascular_measure

<http://www.co-ode.org/ontologies/uia/ont.owl#Cardiovascular_measure> rdf:type owl:Class ;

owl:equivalentClass [ rdf:type owl:Restriction ;

owl:onProperty <http://www.co-ode.org/ontologies/uia/ont.owl#hasSCTID> ;

owl:hasValue "310611001"^^xsd:long

] ;

rdfs:subClassOf <http://www.co-ode.org/ontologies/uia/ont.owl#Cardiovascular_observable> ;

rdfs:comment """Cardiovascular measure (observable entity)

SCTID: 310611001

310611001 | Cardiovascular measure (observable entity) |

en Cardiovascular measure

en Cardiovascular measurement

en Cardiovascular measure (observable entity)

en Cardiovascular measurement - observation""" .

### http://www.co-ode.org/ontologies/uia/ont.owl#Cardiovascular_observable

<http://www.co-ode.org/ontologies/uia/ont.owl#Cardiovascular_observable> rdf:type owl:Class ;

owl:equivalentClass [ rdf:type owl:Restriction ;

owl:onProperty <http://www.co-ode.org/ontologies/uia/ont.owl#hasSCTID> ;

owl:hasValue "364066008"^^xsd:long

] ;

rdfs:subClassOf <http://www.co-ode.org/ontologies/uia/ont.owl#Clinical_history_examination_observable> ;

rdfs:comment """Cardiovascular observable (observable entity)

SCTID: 364066008

364066008 | Cardiovascular observable (observable entity) |

en Cardiovascular observable

en Cardiovascular observable (observable entity)""" .

### http://www.co-ode.org/ontologies/uia/ont.owl#Circumference_measure

<http://www.co-ode.org/ontologies/uia/ont.owl#Circumference_measure> rdf:type owl:Class ;

owl:equivalentClass [ rdf:type owl:Restriction ;

owl:onProperty <http://www.co-ode.org/ontologies/uia/ont.owl#hasSCTID> ;

owl:hasValue "248365001"^^xsd:long

] ;

rdfs:subClassOf <http://www.co-ode.org/ontologies/uia/ont.owl#Body_measure> ;

rdfs:comment """Circumference measure (observable entity)

SCTID: 248365001

248365001 | Circumference measure (observable entity) |

en Circumference measure

en Circumference measure (observable entity)

en Circumference measurements""" .

### http://www.co-ode.org/ontologies/uia/ont.owl#ClinicalFinding

<http://www.co-ode.org/ontologies/uia/ont.owl#ClinicalFinding> rdf:type owl:Class ;

owl:equivalentClass [ rdf:type owl:Restriction ;

owl:onProperty <http://www.co-ode.org/ontologies/uia/ont.owl#hasSCTID> ;

owl:hasValue "404684003"^^xsd:long

] ;

rdfs:subClassOf <http://www.co-ode.org/ontologies/uia/ont.owl#SNOMED_CT_Concept> ;

rdfs:comment """Clinical finding (finding)

SCTID: 404684003

404684003 | Clinical finding (finding) |

en Clinical finding (finding)

en Clinical finding""" .

### http://www.co-ode.org/ontologies/uia/ont.owl#Clinical_history_and_observation_finding

<http://www.co-ode.org/ontologies/uia/ont.owl#Clinical_history_and_observation_finding> rdf:type owl:Class ;

owl:equivalentClass [ rdf:type owl:Restriction ;

owl:onProperty <http://www.co-ode.org/ontologies/uia/ont.owl#hasSCTID> ;

owl:hasValue "250171008"^^xsd:long

] ;

rdfs:subClassOf <http://www.co-ode.org/ontologies/uia/ont.owl#ClinicalFinding> ;

rdfs:comment """Clinical history and observation findings (finding)

SCTID: 250171008

250171008 | Clinical history and observation findings (finding) |

en Clinical history and observation findings

en Clinical history and observation findings (finding)

en Clinical history and observations""" .

### http://www.co-ode.org/ontologies/uia/ont.owl#Clinical_history_examination_observable

<http://www.co-ode.org/ontologies/uia/ont.owl#Clinical_history_examination_observable> rdf:type owl:Class ;

owl:equivalentClass [ rdf:type owl:Restriction ;

owl:onProperty <http://www.co-ode.org/ontologies/uia/ont.owl#hasSCTID> ;

owl:hasValue "363788007"^^xsd:long

] ;

rdfs:subClassOf <http://www.co-ode.org/ontologies/uia/ont.owl#ObservableEntity> ;

rdfs:comment """Clinical history/examination observable (observable entity)

SCTID: 363788007

363788007 | Clinical history/examination observable (observable entity) |

en Clinical history/examination observable (observable entity)

en Clinical history/examination observable""" .

### http://www.co-ode.org/ontologies/uia/ont.owl#CodedSimulatedInputData

<http://www.co-ode.org/ontologies/uia/ont.owl#CodedSimulatedInputData> rdf:type owl:Class ;

rdfs:subClassOf <http://www.co-ode.org/ontologies/uia/ont.owl#SimulatedInputData> .

### http://www.co-ode.org/ontologies/uia/ont.owl#ContextualData

<http://www.co-ode.org/ontologies/uia/ont.owl#ContextualData> rdf:type owl:Class ;

rdfs:subClassOf [ rdf:type owl:Restriction ;

owl:onProperty <http://www.co-ode.org/ontologies/uia/ont.owl#hasBeenCollectedBy> ;

owl:someValuesFrom <http://www.co-ode.org/ontologies/uia/ont.owl#ExternalWeatherValue>

] ,

[ rdf:type owl:Restriction ;

owl:onProperty <http://www.co-ode.org/ontologies/uia/ont.owl#hasTimeStamp> ;

owl:someValuesFrom <http://www.co-ode.org/ontologies/uia/ont.owl#TemporalEntity>

] .

### http://www.co-ode.org/ontologies/uia/ont.owl#Current_body_weight

<http://www.co-ode.org/ontologies/uia/ont.owl#Current_body_weight> rdf:type owl:Class ;

owl:equivalentClass [ rdf:type owl:Restriction ;

owl:onProperty <http://www.co-ode.org/ontologies/uia/ont.owl#hasSCTID> ;

owl:hasValue "735395000"^^xsd:long

] ;

rdfs:subClassOf <http://www.co-ode.org/ontologies/uia/ont.owl#Reference_weight> ;

rdfs:comment """Current body weight (observable entity)

SCTID: 735395000

735395000 | Current body weight (observable entity) |

en Current body weight

en Current body weight (observable entity)""" .

### http://www.co-ode.org/ontologies/uia/ont.owl#Diastolic_blood_pressure

<http://www.co-ode.org/ontologies/uia/ont.owl#Diastolic_blood_pressure> rdf:type owl:Class ;

owl:equivalentClass [ rdf:type owl:Restriction ;

owl:onProperty <http://www.co-ode.org/ontologies/uia/ont.owl#hasSCTID> ;

owl:hasValue "271650006"^^xsd:long

] ;

rdfs:subClassOf <http://www.co-ode.org/ontologies/uia/ont.owl#Blood_pressure> ,

[ rdf:type owl:Restriction ;

owl:onProperty ssn:isFoundBy ;

owl:qualifiedCardinality "1"^^xsd:nonNegativeInteger ;

owl:onClass <http://www.co-ode.org/ontologies/uia/ont.owl#On_examination_Diastolic_blood_pressure_reading>

] .

### http://www.co-ode.org/ontologies/uia/ont.owl#DietaryRecommendation

<http://www.co-ode.org/ontologies/uia/ont.owl#DietaryRecommendation> rdf:type owl:Class ;

rdfs:subClassOf <http://www.co-ode.org/ontologies/uia/ont.owl#Recommendation> .

### http://www.co-ode.org/ontologies/uia/ont.owl#Dietary_finding

<http://www.co-ode.org/ontologies/uia/ont.owl#Dietary_finding> rdf:type owl:Class ;

owl:equivalentClass [ rdf:type owl:Restriction ;

owl:onProperty <http://www.co-ode.org/ontologies/uia/ont.owl#hasSCTID> ;

owl:hasValue "41829006"^^xsd:long

] ;

rdfs:subClassOf <http://www.co-ode.org/ontologies/uia/ont.owl#Nutritional_finding> ;

rdfs:comment """Dietary finding (finding)

SCTID: 41829006

41829006 | Dietary finding (finding) |

en Diets

en Diet

en Dietary finding (finding)

en Dietary finding""" .

### http://www.co-ode.org/ontologies/uia/ont.owl#Endocrine_nutritional_and_metabolic_observable

<http://www.co-ode.org/ontologies/uia/ont.owl#Endocrine_nutritional_and_metabolic_observable> rdf:type owl:Class ;

owl:equivalentClass [ rdf:type owl:Restriction ;

owl:onProperty <http://www.co-ode.org/ontologies/uia/ont.owl#hasSCTID> ;

owl:hasValue "364386000"^^xsd:long

] ;

rdfs:subClassOf <http://www.co-ode.org/ontologies/uia/ont.owl#Clinical_history_examination_observable> ;

rdfs:comment """Endocrine, nutritional and metabolic observable (observable entity)

SCTID: 364386000

364386000 | Endocrine, nutritional and metabolic observable (observable entity) |

en Endocrine, nutritional and metabolic observable (observable entity)

en Endocrine, nutritional and metabolic observable""" .

### http://www.co-ode.org/ontologies/uia/ont.owl#Evaluation_finding

<http://www.co-ode.org/ontologies/uia/ont.owl#Evaluation_finding> rdf:type owl:Class ;

owl:equivalentClass [ rdf:type owl:Restriction ;

owl:onProperty <http://www.co-ode.org/ontologies/uia/ont.owl#hasSCTID> ;

owl:hasValue "441742003"^^xsd:long

] ;

rdfs:subClassOf <http://www.co-ode.org/ontologies/uia/ont.owl#ClinicalFinding> ;

rdfs:comment """Evaluation finding (finding)

SCTID: 441742003

441742003 | Evaluation finding (finding) |

en Evaluation finding

en Evaluation finding (finding)""" .

### http://www.co-ode.org/ontologies/uia/ont.owl#Exercise

<http://www.co-ode.org/ontologies/uia/ont.owl#Exercise> rdf:type owl:Class ;

owl:equivalentClass [ rdf:type owl:Restriction ;

owl:onProperty <http://www.co-ode.org/ontologies/uia/ont.owl#hasSCTID> ;

owl:hasValue "256235009"^^xsd:long

] ;

rdfs:subClassOf <http://www.co-ode.org/ontologies/uia/ont.owl#Activity> ;

rdfs:comment """Exercise (observable entity)

SCTID: 256235009

256235009 | Exercise (observable entity) |

en Exercise (observable entity)

en Exercise""" .

### http://www.co-ode.org/ontologies/uia/ont.owl#ExternalWeatherSensor

<http://www.co-ode.org/ontologies/uia/ont.owl#ExternalWeatherSensor> rdf:type owl:Class ;

rdfs:subClassOf ssn:Sensor .

### http://www.co-ode.org/ontologies/uia/ont.owl#ExternalWeatherValue

<http://www.co-ode.org/ontologies/uia/ont.owl#ExternalWeatherValue> rdf:type owl:Class ;

rdfs:subClassOf ssn:ObservationValue ,

[ rdf:type owl:Restriction ;

owl:onProperty <http://www.co-ode.org/ontologies/uia/ont.owl#hasFoggyForeCast> ;

owl:qualifiedCardinality "1"^^xsd:nonNegativeInteger ;

owl:onDataRange xsd:string

] ,

[ rdf:type owl:Restriction ;

owl:onProperty <http://www.co-ode.org/ontologies/uia/ont.owl#hasHighTemperatureForeCast> ;

owl:qualifiedCardinality "1"^^xsd:nonNegativeInteger ;

owl:onDataRange xsd:string

] ,

[ rdf:type owl:Restriction ;

owl:onProperty <http://www.co-ode.org/ontologies/uia/ont.owl#hasLowTemperatureForeCast> ;

owl:qualifiedCardinality "1"^^xsd:nonNegativeInteger ;

owl:onDataRange xsd:string

] ,

[ rdf:type owl:Restriction ;

owl:onProperty <http://www.co-ode.org/ontologies/uia/ont.owl#hasRainingForeCast> ;

owl:qualifiedCardinality "1"^^xsd:nonNegativeInteger ;

owl:onDataRange xsd:string

] ,

[ rdf:type owl:Restriction ;

owl:onProperty <http://www.co-ode.org/ontologies/uia/ont.owl#hasSnowingForeCast> ;

owl:qualifiedCardinality "1"^^xsd:nonNegativeInteger ;

owl:onDataRange xsd:string

] ,

[ rdf:type owl:Restriction ;

owl:onProperty <http://www.co-ode.org/ontologies/uia/ont.owl#hasSunnyForeCast> ;

owl:qualifiedCardinality "1"^^xsd:nonNegativeInteger ;

owl:onDataRange xsd:string

] ,

[ rdf:type owl:Restriction ;

owl:onProperty <http://www.co-ode.org/ontologies/uia/ont.owl#hasTemperature> ;

owl:qualifiedCardinality "1"^^xsd:nonNegativeInteger ;

owl:onDataRange xsd:string

] ,

[ rdf:type owl:Restriction ;

owl:onProperty <http://www.co-ode.org/ontologies/uia/ont.owl#hasWeatherSatus> ;

owl:qualifiedCardinality "1"^^xsd:nonNegativeInteger ;

owl:onDataRange xsd:string

] .

### http://www.co-ode.org/ontologies/uia/ont.owl#Finding_of_alcohol_intake

<http://www.co-ode.org/ontologies/uia/ont.owl#Finding_of_alcohol_intake> rdf:type owl:Class ;

owl:equivalentClass [ rdf:type owl:Restriction ;

owl:onProperty <http://www.co-ode.org/ontologies/uia/ont.owl#hasSCTID> ;

owl:hasValue "365967005"^^xsd:long

] ;

rdfs:subClassOf <http://www.co-ode.org/ontologies/uia/ont.owl#Finding_of_food_and_drink_intake> ;

rdfs:comment """Finding of alcohol intake (finding)

SCTID: 365967005

365967005 | Finding of alcohol intake (finding) |

en Finding of alcohol intake

en Finding of alcohol intake (finding)

en Alcohol intake - finding""" .

### http://www.co-ode.org/ontologies/uia/ont.owl#Finding_of_food_and_drink_intake

<http://www.co-ode.org/ontologies/uia/ont.owl#Finding_of_food_and_drink_intake> rdf:type owl:Class ;

owl:equivalentClass [ rdf:type owl:Restriction ;

owl:onProperty <http://www.co-ode.org/ontologies/uia/ont.owl#hasSCTID> ;

owl:hasValue "366356005"^^xsd:long

] ;

rdfs:subClassOf <http://www.co-ode.org/ontologies/uia/ont.owl#Nutritional_finding> ;

rdfs:comment """Finding of food and drink intake (finding)

SCTID: 366356005

366356005 | Finding of food and drink intake (finding) |

en Finding of food and drink intake

en Finding of food and drink intake (finding)

en Food and drink intake - finding""" .

### http://www.co-ode.org/ontologies/uia/ont.owl#Finding_of_glucose_level

<http://www.co-ode.org/ontologies/uia/ont.owl#Finding_of_glucose_level> rdf:type owl:Class ;

owl:equivalentClass [ rdf:type owl:Restriction ;

owl:onProperty <http://www.co-ode.org/ontologies/uia/ont.owl#hasSCTID> ;

owl:hasValue "365811003"^^xsd:long

] ;

rdfs:subClassOf <http://www.co-ode.org/ontologies/uia/ont.owl#Finding_of_substance_level> ;

rdfs:comment """Finding of glucose level (finding)

SCTID: 365811003

365811003 | Finding of glucose level (finding) |

en Finding of glucose level

en Glucose level - finding

en Glucose level

en Finding of glucose level (finding)""" .

### http://www.co-ode.org/ontologies/uia/ont.owl#Finding_of_rate_of_respiration

<http://www.co-ode.org/ontologies/uia/ont.owl#Finding_of_rate_of_respiration> rdf:type owl:Class ;

owl:equivalentClass [ rdf:type owl:Restriction ;

owl:onProperty <http://www.co-ode.org/ontologies/uia/ont.owl#hasSCTID> ;

owl:hasValue "301283003"^^xsd:long

] ;

rdfs:subClassOf <http://www.co-ode.org/ontologies/uia/ont.owl#Respiratory_rate_AND/OR_rhythm_finding> ;

rdfs:comment """Finding of rate of respiration (finding)

SCTID: 301283003

301283003 | Finding of rate of respiration (finding) |

en Finding of rate of respiration

en Finding of rate of respiration (finding)

en Observation of rate of respiration""" .

### http://www.co-ode.org/ontologies/uia/ont.owl#Finding_of_substance_level

<http://www.co-ode.org/ontologies/uia/ont.owl#Finding_of_substance_level> rdf:type owl:Class ;

owl:equivalentClass [ rdf:type owl:Restriction ;

owl:onProperty <http://www.co-ode.org/ontologies/uia/ont.owl#hasSCTID> ;

owl:hasValue "785671009"^^xsd:long

] ;

rdfs:subClassOf <http://www.co-ode.org/ontologies/uia/ont.owl#Measurement_finding> ;

rdfs:comment """Finding of substance level (finding)

SCTID: 785671009

785671009 | Finding of substance level (finding) |

en Finding of substance level (finding)

en Finding of substance level""" .

### http://www.co-ode.org/ontologies/uia/ont.owl#Finding_of_systemic_arterial_pressure

<http://www.co-ode.org/ontologies/uia/ont.owl#Finding_of_systemic_arterial_pressure> rdf:type owl:Class ;

owl:equivalentClass [ rdf:type owl:Restriction ;

owl:onProperty <http://www.co-ode.org/ontologies/uia/ont.owl#hasSCTID> ;

owl:hasValue "301140001"^^xsd:long

] ;

rdfs:subClassOf <http://www.co-ode.org/ontologies/uia/ont.owl#Blood_pressure_finding> ;

rdfs:comment """Finding of systemic arterial pressure (finding)

SCTID: 301140001

301140001 | Finding of systemic arterial pressure (finding) |

en Finding of systemic arterial pressure

en Finding of systemic arterial pressure (finding)

en Observation of systemic arterial pressure""" .

### http://www.co-ode.org/ontologies/uia/ont.owl#Fit_for_activity_finding

<http://www.co-ode.org/ontologies/uia/ont.owl#Fit_for_activity_finding> rdf:type owl:Class ;

owl:equivalentClass [ rdf:type owl:Restriction ;

owl:onProperty <http://www.co-ode.org/ontologies/uia/ont.owl#hasSCTID> ;

owl:hasValue "314958004"^^xsd:long

] ;

rdfs:subClassOf <http://www.co-ode.org/ontologies/uia/ont.owl#Finding_of_substance_level> ;

rdfs:comment """Fit for activity (finding)

SCTID: 314958004

314958004 | Fit for activity (finding) |

en Fit for activity (finding)

en Fit for activity""" .

### http://www.co-ode.org/ontologies/uia/ont.owl#Function

<http://www.co-ode.org/ontologies/uia/ont.owl#Function> rdf:type owl:Class ;

owl:equivalentClass [ rdf:type owl:Restriction ;

owl:onProperty <http://www.co-ode.org/ontologies/uia/ont.owl#hasSCTID> ;

owl:hasValue "246464006"^^xsd:long

] ;

rdfs:subClassOf <http://www.co-ode.org/ontologies/uia/ont.owl#ObservableEntity> ;

rdfs:comment """Function (observable entity)

SCTID: 246464006

246464006 | Function (observable entity) |

en Function (observable entity)

en Function

en Any function or property that is not mainly morphologic or structural, including both measurable and observable features and physiologic actions""" .

### http://www.co-ode.org/ontologies/uia/ont.owl#General_body_state_finding

<http://www.co-ode.org/ontologies/uia/ont.owl#General_body_state_finding> rdf:type owl:Class ;

owl:equivalentClass [ rdf:type owl:Restriction ;

owl:onProperty <http://www.co-ode.org/ontologies/uia/ont.owl#hasSCTID> ;

owl:hasValue "82832008"^^xsd:long

] ;

rdfs:subClassOf <http://www.co-ode.org/ontologies/uia/ont.owl#General_finding_of_observation_of_patient> ;

rdfs:comment """General body state finding (finding)

SCTID: 82832008

82832008 | General body state finding (finding) |

en General body state finding

en General body state finding (finding)

en General well-being""" .

### http://www.co-ode.org/ontologies/uia/ont.owl#General_characteristic_of_patient

<http://www.co-ode.org/ontologies/uia/ont.owl#General_characteristic_of_patient> rdf:type owl:Class ;

owl:equivalentClass [ rdf:type owl:Restriction ;

owl:onProperty <http://www.co-ode.org/ontologies/uia/ont.owl#hasSCTID> ;

owl:hasValue "363789004"^^xsd:long

] ;

rdfs:subClassOf <http://www.co-ode.org/ontologies/uia/ont.owl#Clinical_history_examination_observable> ;

rdfs:comment """General characteristic of patient (observable entity)

SCTID: 363789004

363789004 | General characteristic of patient (observable entity) |

en General characteristic of patient

en General characteristic of patient (observable entity)""" .

### http://www.co-ode.org/ontologies/uia/ont.owl#General_clinical_state

<http://www.co-ode.org/ontologies/uia/ont.owl#General_clinical_state> rdf:type owl:Class ;

owl:equivalentClass [ rdf:type owl:Restriction ;

owl:onProperty <http://www.co-ode.org/ontologies/uia/ont.owl#hasSCTID> ;

owl:hasValue "278844005"^^xsd:long

] ;

rdfs:subClassOf <http://www.co-ode.org/ontologies/uia/ont.owl#ObservableEntity> ;

rdfs:comment """General clinical state (observable entity)

SCTID: 278844005

278844005 | General clinical state (observable entity) |

en General clinical state

en General clinical state (observable entity)

en General clinical states""" .

### http://www.co-ode.org/ontologies/uia/ont.owl#General_finding_of_observation_of_patient

<http://www.co-ode.org/ontologies/uia/ont.owl#General_finding_of_observation_of_patient> rdf:type owl:Class ;

owl:equivalentClass [ rdf:type owl:Restriction ;

owl:onProperty <http://www.co-ode.org/ontologies/uia/ont.owl#hasSCTID> ;

owl:hasValue "118222006"^^xsd:long

] ;

rdfs:subClassOf <http://www.co-ode.org/ontologies/uia/ont.owl#Clinical_history_and_observation_finding> ;

rdfs:comment """General finding of observation of patient (finding)

SCTID: 118222006

118222006 | General finding of observation of patient (finding) |

en General finding of observation of patient

en General observation of patient

en General condition of patient

en General finding of observation of patient (finding)""" .

### http://www.co-ode.org/ontologies/uia/ont.owl#HabitData

<http://www.co-ode.org/ontologies/uia/ont.owl#HabitData> rdf:type owl:Class ;

rdfs:subClassOf <http://www.co-ode.org/ontologies/uia/ont.owl#ParticipantHealthRecord> ,

[ rdf:type owl:Restriction ;

owl:onProperty <http://www.co-ode.org/ontologies/uia/ont.owl#hasBeenCollectedBy> ;

owl:someValuesFrom ssn:DailyHabitData

] .

### http://www.co-ode.org/ontologies/uia/ont.owl#Health_related_behavior

<http://www.co-ode.org/ontologies/uia/ont.owl#Health_related_behavior> rdf:type owl:Class ;

owl:equivalentClass [ rdf:type owl:Restriction ;

owl:onProperty <http://www.co-ode.org/ontologies/uia/ont.owl#hasSCTID> ;

owl:hasValue "228272008"^^xsd:long

] ;

rdfs:subClassOf <http://www.co-ode.org/ontologies/uia/ont.owl#Behavior_observable> ;

rdfs:comment """Health-related behavior (observable entity)

SCTID: 228272008

228272008 | Health-related behavior (observable entity) |

en Health-related behavior

en Health-related behaviour

en Health-related behavior (observable entity)""" .

### http://www.co-ode.org/ontologies/uia/ont.owl#Healthy_diet

<http://www.co-ode.org/ontologies/uia/ont.owl#Healthy_diet> rdf:type owl:Class ;

owl:equivalentClass [ rdf:type owl:Restriction ;

owl:onProperty <http://www.co-ode.org/ontologies/uia/ont.owl#hasSCTID> ;

owl:hasValue "226234005"^^xsd:long

] ;

rdfs:subClassOf <http://www.co-ode.org/ontologies/uia/ont.owl#Dietary_finding> ;

rdfs:comment """Healthy diet (finding)

SCTID: 226234005

226234005 | Healthy diet (finding) |

en Healthy diet

en Healthy diet (finding)""" .

### http://www.co-ode.org/ontologies/uia/ont.owl#Healthy_diet_adherence_behavior

<http://www.co-ode.org/ontologies/uia/ont.owl#Healthy_diet_adherence_behavior> rdf:type owl:Class ;

owl:equivalentClass [ rdf:type owl:Restriction ;

owl:onProperty <http://www.co-ode.org/ontologies/uia/ont.owl#hasSCTID> ;

owl:hasValue "439066001"^^xsd:long

] ;

rdfs:subClassOf <http://www.co-ode.org/ontologies/uia/ont.owl#Personal_health_management_behavior> ,

[ rdf:type owl:Restriction ;

owl:onProperty ssn:isFoundBy ;

owl:qualifiedCardinality "1"^^xsd:nonNegativeInteger ;

owl:onClass <http://www.co-ode.org/ontologies/uia/ont.owl#Nutritional_finding>

] ;

rdfs:comment """Healthy diet adherence behavior (observable entity)

SCTID: 439066001

439066001 | Healthy diet adherence behavior (observable entity) |

en Healthy diet adherence behaviour

en Healthy diet adherence behavior (observable entity)

en Healthy diet adherence behavior""" .

### http://www.co-ode.org/ontologies/uia/ont.owl#Height_growth_measure

<http://www.co-ode.org/ontologies/uia/ont.owl#Height_growth_measure> rdf:type owl:Class ;

owl:equivalentClass [ rdf:type owl:Restriction ;

owl:onProperty <http://www.co-ode.org/ontologies/uia/ont.owl#hasSCTID> ;

owl:hasValue "271603002"^^xsd:long

] ;

rdfs:subClassOf <http://www.co-ode.org/ontologies/uia/ont.owl#Body_measure> ;

rdfs:comment """Height / growth measure (observable entity)

SCTID: 271603002

271603002 | Height / growth measure (observable entity) |

en Height / growth measure

en Height AND/OR growth measure

en Height / growth measure (observable entity)""" .

### http://www.co-ode.org/ontologies/uia/ont.owl#Human

<http://www.co-ode.org/ontologies/uia/ont.owl#Human> rdf:type owl:Class ;

rdfs:subClassOf [ rdf:type owl:Restriction ;

owl:onProperty ssn:hasUserId ;

owl:qualifiedCardinality "1"^^xsd:nonNegativeInteger ;

owl:onDataRange xsd:string

] ,

[ rdf:type owl:Restriction ;

owl:onProperty <http://www.co-ode.org/ontologies/uia/ont.owl#hasPassword> ;

owl:qualifiedCardinality "1"^^xsd:nonNegativeInteger ;

owl:onDataRange xsd:string

] ,

[ rdf:type owl:Restriction ;

owl:onProperty <http://www.co-ode.org/ontologies/uia/ont.owl#hasRole> ;

owl:qualifiedCardinality "1"^^xsd:nonNegativeInteger ;

owl:onDataRange xsd:string

] .

### http://www.co-ode.org/ontologies/uia/ont.owl#Interview

<http://www.co-ode.org/ontologies/uia/ont.owl#Interview> rdf:type owl:Class ;

rdfs:subClassOf ssn:Process ,

[ rdf:type owl:Restriction ;

owl:onProperty <http://www.co-ode.org/ontologies/uia/ont.owl#hasConductedBy> ;

owl:someValuesFrom <http://www.co-ode.org/ontologies/uia/ont.owl#Nurse>

] ,

[ rdf:type owl:Restriction ;

owl:onProperty <http://www.co-ode.org/ontologies/uia/ont.owl#hasTimeStamp> ;

owl:someValuesFrom <http://www.co-ode.org/ontologies/uia/ont.owl#TemporalEntity>

] .

### http://www.co-ode.org/ontologies/uia/ont.owl#InterviewedPersonalData

<http://www.co-ode.org/ontologies/uia/ont.owl#InterviewedPersonalData> rdf:type owl:Class ;

rdfs:subClassOf [ rdf:type owl:Restriction ;

owl:onProperty <http://www.co-ode.org/ontologies/uia/ont.owl#hasBeenCollectedBy> ;

owl:someValuesFrom <http://www.co-ode.org/ontologies/uia/ont.owl#Interview>

] ,

[ rdf:type owl:Restriction ;

owl:onProperty <http://www.co-ode.org/ontologies/uia/ont.owl#hasAge> ;

owl:qualifiedCardinality "1"^^xsd:nonNegativeInteger ;

owl:onDataRange xsd:integer

] ,

[ rdf:type owl:Restriction ;

owl:onProperty <http://www.co-ode.org/ontologies/uia/ont.owl#hasEducationalLevel> ;

owl:qualifiedCardinality "1"^^xsd:nonNegativeInteger ;

owl:onDataRange xsd:string

] ,

[ rdf:type owl:Restriction ;

owl:onProperty <http://www.co-ode.org/ontologies/uia/ont.owl#hasEmail> ;

owl:qualifiedCardinality "1"^^xsd:nonNegativeInteger ;

owl:onDataRange xsd:string

] ,

[ rdf:type owl:Restriction ;

owl:onProperty <http://www.co-ode.org/ontologies/uia/ont.owl#hasGender> ;

owl:qualifiedCardinality "1"^^xsd:nonNegativeInteger ;

owl:onDataRange xsd:string

] ,

[ rdf:type owl:Restriction ;

owl:onProperty <http://www.co-ode.org/ontologies/uia/ont.owl#hasIncomeGroup> ;

owl:qualifiedCardinality "1"^^xsd:nonNegativeInteger ;

owl:onDataRange xsd:string

] ,

[ rdf:type owl:Restriction ;

owl:onProperty <http://www.co-ode.org/ontologies/uia/ont.owl#hasMobile> ;

owl:qualifiedCardinality "1"^^xsd:nonNegativeInteger ;

owl:onDataRange xsd:string

] ,

[ rdf:type owl:Restriction ;

owl:onProperty <http://www.co-ode.org/ontologies/uia/ont.owl#hasSmokingHabit> ;

owl:qualifiedCardinality "1"^^xsd:nonNegativeInteger ;

owl:onDataRange xsd:string

] ,

[ rdf:type owl:Restriction ;

owl:onProperty <http://www.co-ode.org/ontologies/uia/ont.owl#hasSnusHabit> ;

owl:qualifiedCardinality "1"^^xsd:nonNegativeInteger ;

owl:onDataRange xsd:string

] ,

[ rdf:type owl:Restriction ;

owl:onProperty <http://www.co-ode.org/ontologies/uia/ont.owl#hasSocialParticipationType> ;

owl:qualifiedCardinality "1"^^xsd:nonNegativeInteger ;

owl:onDataRange xsd:string

] ,

[ rdf:type owl:Restriction ;

owl:onProperty <http://www.co-ode.org/ontologies/uia/ont.owl#hasSoialParticipationFrequency> ;

owl:qualifiedCardinality "1"^^xsd:nonNegativeInteger ;

owl:onDataRange xsd:string

] .

### http://www.co-ode.org/ontologies/uia/ont.owl#Measured_body_weight

<http://www.co-ode.org/ontologies/uia/ont.owl#Measured_body_weight> rdf:type owl:Class ;

owl:equivalentClass [ rdf:type owl:Restriction ;

owl:onProperty <http://www.co-ode.org/ontologies/uia/ont.owl#hasSCTID> ;

owl:hasValue "363808001"^^xsd:long

] ;

rdfs:subClassOf <http://www.co-ode.org/ontologies/uia/ont.owl#Body_weight_characteristic> ;

rdfs:comment """Measured body weight (observable entity)

SCTID: 363808001

363808001 | Measured body weight (observable entity) |

en Measured body weight (observable entity)

en Body weight measure

en Measured body weight""" .

### http://www.co-ode.org/ontologies/uia/ont.owl#Measurement_finding

<http://www.co-ode.org/ontologies/uia/ont.owl#Measurement_finding> rdf:type owl:Class ;

owl:equivalentClass [ rdf:type owl:Restriction ;

owl:onProperty <http://www.co-ode.org/ontologies/uia/ont.owl#hasSCTID> ;

owl:hasValue "118245000"^^xsd:long

] ;

rdfs:subClassOf <http://www.co-ode.org/ontologies/uia/ont.owl#Evaluation_finding> ;

rdfs:comment """Measurement finding (finding)

SCTID: 118245000

118245000 | Measurement finding (finding) |

en Measurement finding (finding)

en Measurement finding""" .

### http://www.co-ode.org/ontologies/uia/ont.owl#Mental_state_behavior_psychosocial_function_observable

<http://www.co-ode.org/ontologies/uia/ont.owl#Mental_state_behavior_psychosocial_function_observable> rdf:type owl:Class ;

owl:equivalentClass [ rdf:type owl:Restriction ;

owl:onProperty <http://www.co-ode.org/ontologies/uia/ont.owl#hasSCTID> ;

owl:hasValue "363870007"^^xsd:long

] ;

rdfs:subClassOf <http://www.co-ode.org/ontologies/uia/ont.owl#Clinical_history_examination_observable> ;

rdfs:comment """Mental state, behavior / psychosocial function observable (observable entity)

SCTID: 363870007

363870007 | Mental state, behavior / psychosocial function observable (observable entity) |

en Mental state, behavior / psychosocial function observable

en Mental state, behaviour / psychosocial function observable

en Mental state, behavior / psychosocial function observable (observable entity)""" .

### http://www.co-ode.org/ontologies/uia/ont.owl#Normal_weight_finding

<http://www.co-ode.org/ontologies/uia/ont.owl#Normal_weight_finding> rdf:type owl:Class ;

owl:equivalentClass [ rdf:type owl:Restriction ;

owl:onProperty <http://www.co-ode.org/ontologies/uia/ont.owl#hasSCTID> ;

owl:hasValue "43664005"^^xsd:long

] ;

rdfs:subClassOf <http://www.co-ode.org/ontologies/uia/ont.owl#Weight_finding> ;

rdfs:comment """Normal weight (finding)

SCTID: 43664005

43664005 | Normal weight (finding) |

en Normal weight

en Normal weight (finding)""" .

### http://www.co-ode.org/ontologies/uia/ont.owl#Nurse

<http://www.co-ode.org/ontologies/uia/ont.owl#Nurse> rdf:type owl:Class ;

rdfs:subClassOf <http://www.co-ode.org/ontologies/uia/ont.owl#Human> ,

[ rdf:type owl:Restriction ;

owl:onProperty <http://www.co-ode.org/ontologies/uia/ont.owl#hasOfficeAddress> ;

owl:someValuesFrom <http://www.co-ode.org/ontologies/uia/ont.owl#OfficeAddress>

] ,

[ rdf:type owl:Restriction ;

owl:onProperty <http://www.co-ode.org/ontologies/uia/ont.owl#hasPersonalData> ;

owl:someValuesFrom <http://www.co-ode.org/ontologies/uia/ont.owl#PersonalData>

] .

### http://www.co-ode.org/ontologies/uia/ont.owl#NutritionData

<http://www.co-ode.org/ontologies/uia/ont.owl#NutritionData> rdf:type owl:Class ;

rdfs:subClassOf <http://www.co-ode.org/ontologies/uia/ont.owl#ParticipantHealthRecord> ,

[ rdf:type owl:Restriction ;

owl:onProperty <http://www.co-ode.org/ontologies/uia/ont.owl#hasBeenCollectedBy> ;

owl:someValuesFrom ssn:DailyNutritionData

] .

### http://www.co-ode.org/ontologies/uia/ont.owl#Nutritional_finding

<http://www.co-ode.org/ontologies/uia/ont.owl#Nutritional_finding> rdf:type owl:Class ;

owl:equivalentClass [ rdf:type owl:Restriction ;

owl:onProperty <http://www.co-ode.org/ontologies/uia/ont.owl#hasSCTID> ;

owl:hasValue "300893006"^^xsd:long

] ;

rdfs:subClassOf <http://www.co-ode.org/ontologies/uia/ont.owl#Body_measurement_finding> ;

rdfs:comment """Nutritional finding (finding)

SCTID: 300893006

300893006 | Nutritional finding (finding) |

en Nutritional finding (finding)

en Nutritional observation

en Nutritional finding""" .

### http://www.co-ode.org/ontologies/uia/ont.owl#Nutritional_observable

<http://www.co-ode.org/ontologies/uia/ont.owl#Nutritional_observable> rdf:type owl:Class ;

owl:equivalentClass [ rdf:type owl:Restriction ;

owl:onProperty <http://www.co-ode.org/ontologies/uia/ont.owl#hasSCTID> ;

owl:hasValue "364393001"^^xsd:long

] ;

rdfs:subClassOf <http://www.co-ode.org/ontologies/uia/ont.owl#Endocrine_nutritional_and_metabolic_observable> ;

rdfs:comment """Nutritional observable (observable entity)

SCTID: 364393001

364393001 | Nutritional observable (observable entity) |

en Nutritional observable

en Nutritional observable (observable entity)""" .

### http://www.co-ode.org/ontologies/uia/ont.owl#Nutritional_status

<http://www.co-ode.org/ontologies/uia/ont.owl#Nutritional_status> rdf:type owl:Class ;

owl:equivalentClass [ rdf:type owl:Restriction ;

owl:onProperty <http://www.co-ode.org/ontologies/uia/ont.owl#hasSCTID> ;

owl:hasValue "87276001"^^xsd:long

] ;

rdfs:subClassOf <http://www.co-ode.org/ontologies/uia/ont.owl#Nutritional_observable> ;

rdfs:comment """Nutritional status (observable entity)

SCTID: 87276001

87276001 | Nutritional status (observable entity) |

en Nutritional status (observable entity)

en Dietary status

en Nutritional status""" .

### http://www.co-ode.org/ontologies/uia/ont.owl#Obese_finding

<http://www.co-ode.org/ontologies/uia/ont.owl#Obese_finding> rdf:type owl:Class ;

owl:equivalentClass [ rdf:type owl:Restriction ;

owl:onProperty <http://www.co-ode.org/ontologies/uia/ont.owl#hasSCTID> ;

owl:hasValue "414915002"^^xsd:long

] ;

rdfs:subClassOf <http://www.co-ode.org/ontologies/uia/ont.owl#Overweight_finding> ;

rdfs:comment """Obese (finding)

SCTID: 414915002

414915002 | Obese (finding) |

en Obese (finding)

en Obese""" .

### http://www.co-ode.org/ontologies/uia/ont.owl#ObservableEntity

<http://www.co-ode.org/ontologies/uia/ont.owl#ObservableEntity> rdf:type owl:Class ;

owl:equivalentClass [ rdf:type owl:Restriction ;

owl:onProperty <http://www.co-ode.org/ontologies/uia/ont.owl#hasSCTID> ;

owl:hasValue "363787002"^^xsd:long

] ;

rdfs:subClassOf <http://www.co-ode.org/ontologies/uia/ont.owl#SNOMED_CT_Concept> ;

rdfs:comment """Observable entity (observable entity)

SCTID: 363787002

363787002 | Observable entity (observable entity) |

en Observable entity

en Observable entity (observable entity)""" .

### http://www.co-ode.org/ontologies/uia/ont.owl#OfficeAddress

<http://www.co-ode.org/ontologies/uia/ont.owl#OfficeAddress> rdf:type owl:Class ;

rdfs:subClassOf [ rdf:type owl:Restriction ;

owl:onProperty <http://www.co-ode.org/ontologies/uia/ont.owl#hasOfficePhone> ;

owl:qualifiedCardinality "1"^^xsd:nonNegativeInteger ;

owl:onDataRange xsd:string

] ,

[ rdf:type owl:Restriction ;

owl:onProperty <http://www.co-ode.org/ontologies/uia/ont.owl#hasOfficePostCode> ;

owl:qualifiedCardinality "1"^^xsd:nonNegativeInteger ;

owl:onDataRange xsd:integer

] ,

[ rdf:type owl:Restriction ;

owl:onProperty <http://www.co-ode.org/ontologies/uia/ont.owl#hasRoomNo> ;

owl:qualifiedCardinality "1"^^xsd:nonNegativeInteger ;

owl:onDataRange xsd:string

] .

### http://www.co-ode.org/ontologies/uia/ont.owl#On_examination_Diastolic_blood_pressure_reading

<http://www.co-ode.org/ontologies/uia/ont.owl#On_examination_Diastolic_blood_pressure_reading> rdf:type owl:Class ;

owl:equivalentClass [ rdf:type owl:Restriction ;

owl:onProperty <http://www.co-ode.org/ontologies/uia/ont.owl#hasSCTID> ;

owl:hasValue "163031004"^^xsd:long

] ;

rdfs:subClassOf <http://www.co-ode.org/ontologies/uia/ont.owl#Finding_of_systemic_arterial_pressure> ;

rdfs:comment """On examination - Diastolic blood pressure reading (finding)

SCTID: 163031004

163031004 | On examination - Diastolic blood pressure reading (finding) |

en O/E - Diastolic BP reading

en On examination - Diastolic blood pressure reading (finding)

en On examination - Diastolic blood pressure reading

en On examination - Diastolic BP reading""" .

### http://www.co-ode.org/ontologies/uia/ont.owl#On_examination_Systolic_blood_pressure_reading

<http://www.co-ode.org/ontologies/uia/ont.owl#On_examination_Systolic_blood_pressure_reading> rdf:type owl:Class ;

owl:equivalentClass [ rdf:type owl:Restriction ;

owl:onProperty <http://www.co-ode.org/ontologies/uia/ont.owl#hasSCTID> ;

owl:hasValue "163030003"^^xsd:long

] ;

rdfs:subClassOf <http://www.co-ode.org/ontologies/uia/ont.owl#Finding_of_systemic_arterial_pressure> ;

rdfs:comment """On examination - Systolic blood pressure reading (finding)

SCTID: 163030003

163030003 | On examination - Systolic blood pressure reading (finding) |

en O/E - Systolic BP reading

en On examination - Systolic BP reading

en On examination - Systolic blood pressure reading (finding)

en On examination - Systolic blood pressure reading""" .

### http://www.co-ode.org/ontologies/uia/ont.owl#On_examination_weight

<http://www.co-ode.org/ontologies/uia/ont.owl#On_examination_weight> rdf:type owl:Class ;

owl:equivalentClass [ rdf:type owl:Restriction ;

owl:onProperty <http://www.co-ode.org/ontologies/uia/ont.owl#hasSCTID> ;

owl:hasValue "162766004"^^xsd:long

] ;

rdfs:subClassOf <http://www.co-ode.org/ontologies/uia/ont.owl#Normal_weight_finding> ;

rdfs:comment """On examination - weight within 10% ideal (finding)

SCTID: 162766004

162766004 | On examination - weight within 10% ideal (finding) |

en On examination - weight within 10% ideal

en On examination - weight within 10% ideal (finding)

en O/E - weight within 10% ideal""" .

### http://www.co-ode.org/ontologies/uia/ont.owl#Overweight_finding

<http://www.co-ode.org/ontologies/uia/ont.owl#Overweight_finding> rdf:type owl:Class ;

owl:equivalentClass [ rdf:type owl:Restriction ;

owl:onProperty <http://www.co-ode.org/ontologies/uia/ont.owl#hasSCTID> ;

owl:hasValue "238131007"^^xsd:long

] ;

rdfs:subClassOf <http://www.co-ode.org/ontologies/uia/ont.owl#Weight_finding> ;

rdfs:comment """Overweight (finding)

SCTID: 238131007

238131007 | Overweight (finding) |

en Patient overweight

en Overweight

en Overweight (finding)""" .

### http://www.co-ode.org/ontologies/uia/ont.owl#Participant

<http://www.co-ode.org/ontologies/uia/ont.owl#Participant> rdf:type owl:Class ;

rdfs:subClassOf <http://www.co-ode.org/ontologies/uia/ont.owl#Human> ,

[ rdf:type owl:Restriction ;

owl:onProperty <http://www.co-ode.org/ontologies/uia/ont.owl#hasHealthRecord> ;

owl:someValuesFrom <http://www.co-ode.org/ontologies/uia/ont.owl#ParticipantHealthRecord>

] ,

[ rdf:type owl:Restriction ;

owl:onProperty <http://www.co-ode.org/ontologies/uia/ont.owl#hasInterviewPersonalData> ;

owl:someValuesFrom <http://www.co-ode.org/ontologies/uia/ont.owl#InterviewedPersonalData>

] ,

[ rdf:type owl:Restriction ;

owl:onProperty <http://www.co-ode.org/ontologies/uia/ont.owl#hasReceivedRecommendation> ;

owl:someValuesFrom <http://www.co-ode.org/ontologies/uia/ont.owl#Recommendation>

] ,

[ rdf:type owl:Restriction ;

owl:onProperty <http://www.co-ode.org/ontologies/uia/ont.owl#hasStatus> ;

owl:someValuesFrom <http://www.co-ode.org/ontologies/uia/ont.owl#ParticipantStatus>

] .

### http://www.co-ode.org/ontologies/uia/ont.owl#ParticipantHealthRecord

<http://www.co-ode.org/ontologies/uia/ont.owl#ParticipantHealthRecord> rdf:type owl:Class ;

rdfs:subClassOf [ rdf:type owl:Restriction ;

owl:onProperty ssn:hasObservables ;

owl:someValuesFrom <http://www.co-ode.org/ontologies/uia/ont.owl#ObservableEntity>

] .

### http://www.co-ode.org/ontologies/uia/ont.owl#ParticipantState

<http://www.co-ode.org/ontologies/uia/ont.owl#ParticipantState> rdf:type owl:Class ;

rdfs:subClassOf [ rdf:type owl:Restriction ;

owl:onProperty ssn:hasClinicalFinding ;

owl:someValuesFrom <http://www.co-ode.org/ontologies/uia/ont.owl#ClinicalFinding>

] ,

[ rdf:type owl:Restriction ;

owl:onProperty ssn:hasVitalSignFinding ;

owl:someValuesFrom <http://www.co-ode.org/ontologies/uia/ont.owl#Vital_sign_finding>

] .

### http://www.co-ode.org/ontologies/uia/ont.owl#ParticipantStatus

<http://www.co-ode.org/ontologies/uia/ont.owl#ParticipantStatus> rdf:type owl:Class ;

rdfs:subClassOf [ rdf:type owl:Restriction ;

owl:onProperty <http://www.co-ode.org/ontologies/uia/ont.owl#hasParticipantState> ;

owl:qualifiedCardinality "1"^^xsd:nonNegativeInteger ;

owl:onClass <http://www.co-ode.org/ontologies/uia/ont.owl#ParticipantState>

] ,

[ rdf:type owl:Restriction ;

owl:onProperty <http://www.co-ode.org/ontologies/uia/ont.owl#hasParticipationStatus> ;

owl:qualifiedCardinality "1"^^xsd:nonNegativeInteger ;

owl:onDataRange xsd:string

] .

### http://www.co-ode.org/ontologies/uia/ont.owl#PersonalData

<http://www.co-ode.org/ontologies/uia/ont.owl#PersonalData> rdf:type owl:Class ;

rdfs:subClassOf [ rdf:type owl:Restriction ;

owl:onProperty <http://www.co-ode.org/ontologies/uia/ont.owl#hasAge> ;

owl:qualifiedCardinality "1"^^xsd:nonNegativeInteger ;

owl:onDataRange xsd:integer

] ,

[ rdf:type owl:Restriction ;

owl:onProperty <http://www.co-ode.org/ontologies/uia/ont.owl#hasDesignation> ;

owl:qualifiedCardinality "1"^^xsd:nonNegativeInteger ;

owl:onDataRange xsd:string

] ,

[ rdf:type owl:Restriction ;

owl:onProperty <http://www.co-ode.org/ontologies/uia/ont.owl#hasEmail> ;

owl:qualifiedCardinality "1"^^xsd:nonNegativeInteger ;

owl:onDataRange xsd:string

] ,

[ rdf:type owl:Restriction ;

owl:onProperty <http://www.co-ode.org/ontologies/uia/ont.owl#hasFirstName> ;

owl:qualifiedCardinality "1"^^xsd:nonNegativeInteger ;

owl:onDataRange xsd:string

] ,

[ rdf:type owl:Restriction ;

owl:onProperty <http://www.co-ode.org/ontologies/uia/ont.owl#hasGender> ;

owl:qualifiedCardinality "1"^^xsd:nonNegativeInteger ;

owl:onDataRange xsd:string

] ,

[ rdf:type owl:Restriction ;

owl:onProperty <http://www.co-ode.org/ontologies/uia/ont.owl#hasLastName> ;

owl:qualifiedCardinality "1"^^xsd:nonNegativeInteger ;

owl:onDataRange xsd:string

] ,

[ rdf:type owl:Restriction ;

owl:onProperty <http://www.co-ode.org/ontologies/uia/ont.owl#hasMobile> ;

owl:qualifiedCardinality "1"^^xsd:nonNegativeInteger ;

owl:onDataRange xsd:string

] .

### http://www.co-ode.org/ontologies/uia/ont.owl#Personal_health_management_behavior

<http://www.co-ode.org/ontologies/uia/ont.owl#Personal_health_management_behavior> rdf:type owl:Class ;

owl:equivalentClass [ rdf:type owl:Restriction ;

owl:onProperty <http://www.co-ode.org/ontologies/uia/ont.owl#hasSCTID> ;

owl:hasValue "406211006"^^xsd:long

] ;

rdfs:subClassOf <http://www.co-ode.org/ontologies/uia/ont.owl#Health_related_behavior> ;

rdfs:comment """Personal health management behavior (observable entity)

SCTID: 406211006

406211006 | Personal health management behavior (observable entity) |

en Personal health management behaviour

en Personal health management behavior

en Personal health management behavior (observable entity)""" .

### http://www.co-ode.org/ontologies/uia/ont.owl#PhysicalDeviceThing

<http://www.co-ode.org/ontologies/uia/ont.owl#PhysicalDeviceThing> rdf:type owl:Class .

### http://www.co-ode.org/ontologies/uia/ont.owl#PhysiologicalData

<http://www.co-ode.org/ontologies/uia/ont.owl#PhysiologicalData> rdf:type owl:Class ;

rdfs:subClassOf <http://www.co-ode.org/ontologies/uia/ont.owl#ParticipantHealthRecord> ,

[ rdf:type owl:Restriction ;

owl:onProperty <http://www.co-ode.org/ontologies/uia/ont.owl#hasBeenCollectedBy> ;

owl:someValuesFrom ssn:DailyPhysiologicalData

] ,

[ rdf:type owl:Restriction ;

owl:onProperty <http://www.co-ode.org/ontologies/uia/ont.owl#hasBeenCollectedBy> ;

owl:someValuesFrom <http://www.co-ode.org/ontologies/uia/ont.owl#ActivityDataValue>

] .

### http://www.co-ode.org/ontologies/uia/ont.owl#Process

<http://www.co-ode.org/ontologies/uia/ont.owl#Process> rdf:type owl:Class ;

owl:equivalentClass [ rdf:type owl:Restriction ;

owl:onProperty <http://www.co-ode.org/ontologies/uia/ont.owl#hasSCTID> ;

owl:hasValue "415178003"^^xsd:long

] ;

rdfs:subClassOf <http://www.co-ode.org/ontologies/uia/ont.owl#ObservableEntity> ;

rdfs:comment """Process (observable entity)

SCTID: 415178003

415178003 | Process (observable entity) |

en Process (observable entity)

en Process""" .

### http://www.co-ode.org/ontologies/uia/ont.owl#Pulse_finding

<http://www.co-ode.org/ontologies/uia/ont.owl#Pulse_finding> rdf:type owl:Class ;

owl:equivalentClass [ rdf:type owl:Restriction ;

owl:onProperty <http://www.co-ode.org/ontologies/uia/ont.owl#hasSCTID> ;

owl:hasValue "366199006"^^xsd:long

] ;

rdfs:subClassOf <http://www.co-ode.org/ontologies/uia/ont.owl#Vital_sign_finding> ;

rdfs:comment """Pulse finding (finding)

SCTID: 366199006

366199006 | Pulse finding (finding) |

en Pulse finding

en Pulse finding (finding)""" .

### http://www.co-ode.org/ontologies/uia/ont.owl#Pulse_rate_finding

<http://www.co-ode.org/ontologies/uia/ont.owl#Pulse_rate_finding> rdf:type owl:Class ;

owl:equivalentClass [ rdf:type owl:Restriction ;

owl:onProperty <http://www.co-ode.org/ontologies/uia/ont.owl#hasSCTID> ;

owl:hasValue "301147003"^^xsd:long

] ;

rdfs:subClassOf <http://www.co-ode.org/ontologies/uia/ont.owl#Pulse_finding> ,

[ rdf:type owl:Restriction ;

owl:onProperty <http://www.co-ode.org/ontologies/uia/ont.owl#hasHeartRate> ;

owl:qualifiedCardinality "1"^^xsd:nonNegativeInteger ;

owl:onClass <http://www.co-ode.org/ontologies/uia/ont.owl#Pulse,function>

] ;

rdfs:comment """Pulse rate finding (finding)

SCTID: 301147003

301147003 | Pulse rate finding (finding) |

en Pulse rate finding

en Pulse rate finding (finding)

en Observation of pulse rate""" .

### http://www.co-ode.org/ontologies/uia/ont.owl#Questionnaire

<http://www.co-ode.org/ontologies/uia/ont.owl#Questionnaire> rdf:type owl:Class ;

rdfs:subClassOf ssn:Process ,

[ rdf:type owl:Restriction ;

owl:onProperty <http://www.co-ode.org/ontologies/uia/ont.owl#hasTimeStamp> ;

owl:someValuesFrom <http://www.co-ode.org/ontologies/uia/ont.owl#TemporalEntity>

] .

### http://www.co-ode.org/ontologies/uia/ont.owl#Recommendation

<http://www.co-ode.org/ontologies/uia/ont.owl#Recommendation> rdf:type owl:Class ;

rdfs:subClassOf [ rdf:type owl:Restriction ;

owl:onProperty ssn:hasContextData ;

owl:someValuesFrom <http://www.co-ode.org/ontologies/uia/ont.owl#ContextualData>

] ,

[ rdf:type owl:Restriction ;

owl:onProperty <http://www.co-ode.org/ontologies/uia/ont.owl#hasMessages> ;

owl:someValuesFrom <http://www.co-ode.org/ontologies/uia/ont.owl#RecommendationMessages>

] ,

[ rdf:type owl:Restriction ;

owl:onProperty <http://www.co-ode.org/ontologies/uia/ont.owl#hasParticipantStatus> ;

owl:someValuesFrom <http://www.co-ode.org/ontologies/uia/ont.owl#ParticipantStatus>

] ,

[ rdf:type owl:Restriction ;

owl:onProperty <http://www.co-ode.org/ontologies/uia/ont.owl#hasTimeStamp> ;

owl:someValuesFrom <http://www.co-ode.org/ontologies/uia/ont.owl#TemporalEntity>

] ,

[ rdf:type owl:Restriction ;

owl:onProperty <http://www.co-ode.org/ontologies/uia/ont.owl#hasUsedHealthRecord> ;

owl:someValuesFrom <http://www.co-ode.org/ontologies/uia/ont.owl#ParticipantHealthRecord>

] .

### http://www.co-ode.org/ontologies/uia/ont.owl#RecommendationMessages

<http://www.co-ode.org/ontologies/uia/ont.owl#RecommendationMessages> rdf:type owl:Class ;

rdfs:subClassOf [ rdf:type owl:Restriction ;

owl:onProperty ssn:hasActivityMessages ;

owl:someValuesFrom xsd:string

] ,

[ rdf:type owl:Restriction ;

owl:onProperty ssn:hasContextualMessages ;

owl:someValuesFrom xsd:string

] ,

[ rdf:type owl:Restriction ;

owl:onProperty ssn:hasDietaryMessages ;

owl:someValuesFrom xsd:string

] ,

[ rdf:type owl:Restriction ;

owl:onProperty ssn:hasHabitRelatedMessages ;

owl:someValuesFrom xsd:string

] .

### http://www.co-ode.org/ontologies/uia/ont.owl#Reference_weight

<http://www.co-ode.org/ontologies/uia/ont.owl#Reference_weight> rdf:type owl:Class ;

owl:equivalentClass [ rdf:type owl:Restriction ;

owl:onProperty <http://www.co-ode.org/ontologies/uia/ont.owl#hasSCTID> ;

owl:hasValue "248350002"^^xsd:long

] ;

rdfs:subClassOf <http://www.co-ode.org/ontologies/uia/ont.owl#Body_weight> ;

rdfs:comment """Reference weight (observable entity)

SCTID: 248350002

248350002 | Reference weight (observable entity) |

en Reference weight (observable entity)

en Reference weight""" .

### http://www.co-ode.org/ontologies/uia/ont.owl#Regular_exercise

<http://www.co-ode.org/ontologies/uia/ont.owl#Regular_exercise> rdf:type owl:Class ;

owl:equivalentClass [ rdf:type owl:Restriction ;

owl:onProperty <http://www.co-ode.org/ontologies/uia/ont.owl#hasSCTID> ;

owl:hasValue "225925001"^^xsd:long

] ;

rdfs:subClassOf <http://www.co-ode.org/ontologies/uia/ont.owl#Exercise> ;

rdfs:comment """Regular exercise (observable entity)

SCTID: 225925001

225925001 | Regular exercise (observable entity) |

en Regular exercise (observable entity)

en Regular exercise""" .

### http://www.co-ode.org/ontologies/uia/ont.owl#Researcher

<http://www.co-ode.org/ontologies/uia/ont.owl#Researcher> rdf:type owl:Class ;

rdfs:subClassOf <http://www.co-ode.org/ontologies/uia/ont.owl#Human> ,

[ rdf:type owl:Restriction ;

owl:onProperty <http://www.co-ode.org/ontologies/uia/ont.owl#hasOfficeAddress> ;

owl:someValuesFrom <http://www.co-ode.org/ontologies/uia/ont.owl#OfficeAddress>

] ,

[ rdf:type owl:Restriction ;

owl:onProperty <http://www.co-ode.org/ontologies/uia/ont.owl#hasPersonalData> ;

owl:someValuesFrom <http://www.co-ode.org/ontologies/uia/ont.owl#PersonalData>

] .

### http://www.co-ode.org/ontologies/uia/ont.owl#Respiration_observable

<http://www.co-ode.org/ontologies/uia/ont.owl#Respiration_observable> rdf:type owl:Class ;

owl:equivalentClass [ rdf:type owl:Restriction ;

owl:onProperty <http://www.co-ode.org/ontologies/uia/ont.owl#hasSCTID> ;

owl:hasValue "364062005"^^xsd:long

] ;

rdfs:subClassOf <http://www.co-ode.org/ontologies/uia/ont.owl#Respiratory_observable> ;

rdfs:comment """Respiration observable (observable entity)

SCTID: 364062005

364062005 | Respiration observable (observable entity) |

en Respiration observable

en Respiration observable (observable entity)""" .

### http://www.co-ode.org/ontologies/uia/ont.owl#Respiratory_measure

<http://www.co-ode.org/ontologies/uia/ont.owl#Respiratory_measure> rdf:type owl:Class ;

owl:equivalentClass [ rdf:type owl:Restriction ;

owl:onProperty <http://www.co-ode.org/ontologies/uia/ont.owl#hasSCTID> ;

owl:hasValue "251880004"^^xsd:long

] ;

rdfs:subClassOf <http://www.co-ode.org/ontologies/uia/ont.owl#Respiration_observable> ;

rdfs:comment """Respiratory measure (observable entity)

SCTID: 251880004

251880004 | Respiratory measure (observable entity) |

en Respiratory measure

en Respiratory measurements

en Respiratory measure (observable entity)

en Observation of measures of respiratory function""" .

### http://www.co-ode.org/ontologies/uia/ont.owl#Respiratory_observable

<http://www.co-ode.org/ontologies/uia/ont.owl#Respiratory_observable> rdf:type owl:Class ;

owl:equivalentClass [ rdf:type owl:Restriction ;

owl:onProperty <http://www.co-ode.org/ontologies/uia/ont.owl#hasSCTID> ;

owl:hasValue "364048003"^^xsd:long

] ;

rdfs:subClassOf <http://www.co-ode.org/ontologies/uia/ont.owl#Clinical_history_examination_observable> ;

rdfs:comment """Respiratory observable (observable entity)

SCTID: 364048003

364048003 | Respiratory observable (observable entity) |

en Respiratory observable

en Respiratory observable (observable entity)""" .

### http://www.co-ode.org/ontologies/uia/ont.owl#Respiratory_rate

<http://www.co-ode.org/ontologies/uia/ont.owl#Respiratory_rate> rdf:type owl:Class ;

owl:equivalentClass [ rdf:type owl:Restriction ;

owl:onProperty <http://www.co-ode.org/ontologies/uia/ont.owl#hasSCTID> ;

owl:hasValue "86290005"^^xsd:long

] ;

rdfs:subClassOf <http://www.co-ode.org/ontologies/uia/ont.owl#Respiratory_measure> ,

[ rdf:type owl:Restriction ;

owl:onProperty ssn:isFoundBy ;

owl:qualifiedCardinality "1"^^xsd:nonNegativeInteger ;

owl:onClass <http://www.co-ode.org/ontologies/uia/ont.owl#Finding_of_rate_of_respiration>

] ;

rdfs:comment """Respiratory rate (observable entity)

SCTID: 86290005

86290005 | Respiratory rate (observable entity) |

en Respiratory rate

en Respiratory rate (observable entity)

en BR - Breathing rate

en Respiratory frequency

en Breathing rate

en RR - Respiratory rate

en Rate of respiration""" .

### http://www.co-ode.org/ontologies/uia/ont.owl#SNOMED_CT_Concept

<http://www.co-ode.org/ontologies/uia/ont.owl#SNOMED_CT_Concept> rdf:type owl:Class ;

owl:equivalentClass [ rdf:type owl:Restriction ;

owl:onProperty <http://www.co-ode.org/ontologies/uia/ont.owl#hasSCTID> ;

owl:hasValue "138875005"^^xsd:long

] ;

rdfs:comment """rdfs:comment \"SNOMED CT Concept (SNOMED RT+CTV3)

SCTID: 138875005

138875005 | SNOMED CT Concept (SNOMED RT+CTV3) |

en SNOMED CT Concept

en SNOMED CT has been created by combining SNOMED RT and a computer-based nomenclature and classification known as Read Codes Version 3, which was created on behalf of the U.K. Department of Health.

en SNOMED CT Concept (SNOMED RT+CTV3)

en © 2002-2019 International Health Terminology Standards Development Organisation (IHTSDO). All rights reserved. SNOMED CT®, was originally created by The College of American Pathologists. \"SNOMED\" and \"SNOMED CT\" are registered trademarks of the IHTSDO.

en SNOMED Clinical Terms version: 20190731 [R] (July 2019 Release)""" ;

rdfs:seeAlso "https://browser.ihtsdotools.org/?perspective=full&conceptId1=138875005&edition=MAIN/2019-07-31&release=&languages=en" ;

owl:versionInfo "July 2019 Release" .

### http://www.co-ode.org/ontologies/uia/ont.owl#SNOWMEDCTSimulatedinput

<http://www.co-ode.org/ontologies/uia/ont.owl#SNOWMEDCTSimulatedinput> rdf:type owl:Class ;

rdfs:subClassOf <http://www.co-ode.org/ontologies/uia/ont.owl#CodedSimulatedInputData> ,

[ rdf:type owl:Restriction ;

owl:onProperty <http://www.co-ode.org/ontologies/uia/ont.owl#sCTID> ;

owl:qualifiedCardinality "1"^^xsd:nonNegativeInteger ;

owl:onDataRange xsd:string

] .

### http://www.co-ode.org/ontologies/uia/ont.owl#SimulatedInputData

<http://www.co-ode.org/ontologies/uia/ont.owl#SimulatedInputData> rdf:type owl:Class .

### http://www.co-ode.org/ontologies/uia/ont.owl#SimulatedInputDataSequence

<http://www.co-ode.org/ontologies/uia/ont.owl#SimulatedInputDataSequence> rdf:type owl:Class ;

rdfs:subClassOf [ rdf:type owl:Restriction ;

owl:onProperty <http://www.co-ode.org/ontologies/uia/ont.owl#hasSimulatedInputData> ;

owl:minQualifiedCardinality "0"^^xsd:nonNegativeInteger ;

owl:onClass <http://www.co-ode.org/ontologies/uia/ont.owl#SimulatedInputData>

] ,

[ rdf:type owl:Restriction ;

owl:onProperty <http://www.co-ode.org/ontologies/uia/ont.owl#hasName> ;

owl:qualifiedCardinality "1"^^xsd:nonNegativeInteger ;

owl:onDataRange xsd:string

] .

### http://www.co-ode.org/ontologies/uia/ont.owl#Standing_height

<http://www.co-ode.org/ontologies/uia/ont.owl#Standing_height> rdf:type owl:Class ;

owl:equivalentClass [ rdf:type owl:Restriction ;

owl:onProperty <http://www.co-ode.org/ontologies/uia/ont.owl#hasSCTID> ;

owl:hasValue "248333004"^^xsd:long

] ;

rdfs:subClassOf <http://www.co-ode.org/ontologies/uia/ont.owl#Body_height_measure> ;

rdfs:comment """Standing height (observable entity)

SCTID: 248333004

248333004 | Standing height (observable entity) |

en Standing height (observable entity)

en Standing height""" .

### http://www.co-ode.org/ontologies/uia/ont.owl#Systolic_blood_pressure

<http://www.co-ode.org/ontologies/uia/ont.owl#Systolic_blood_pressure> rdf:type owl:Class ;

owl:equivalentClass [ rdf:type owl:Restriction ;

owl:onProperty <http://www.co-ode.org/ontologies/uia/ont.owl#hasSCTID> ;

owl:hasValue "271649006"^^xsd:long

] ;

rdfs:subClassOf <http://www.co-ode.org/ontologies/uia/ont.owl#Blood_pressure> ,

[ rdf:type owl:Restriction ;

owl:onProperty ssn:isFoundBy ;

owl:qualifiedCardinality "1"^^xsd:nonNegativeInteger ;

owl:onClass <http://www.co-ode.org/ontologies/uia/ont.owl#On_examination_Systolic_blood_pressure_reading>

] .

### http://www.co-ode.org/ontologies/uia/ont.owl#TemporalEntity

<http://www.co-ode.org/ontologies/uia/ont.owl#TemporalEntity> rdf:type owl:Class ;

rdfs:subClassOf [ rdf:type owl:Restriction ;

owl:onProperty ssn:hasObservationTime ;

owl:maxQualifiedCardinality "1"^^xsd:nonNegativeInteger ;

owl:onDataRange xsd:dateTimeStamp

] ,

[ rdf:type owl:Restriction ;

owl:onProperty <http://www.co-ode.org/ontologies/uia/ont.owl#hadLastAppointment> ;

owl:maxQualifiedCardinality "1"^^xsd:nonNegativeInteger ;

owl:onDataRange xsd:dateTimeStamp

] ,

[ rdf:type owl:Restriction ;

owl:onProperty <http://www.co-ode.org/ontologies/uia/ont.owl#hasDateTime> ;

owl:maxQualifiedCardinality "1"^^xsd:nonNegativeInteger ;

owl:onDataRange xsd:dateTimeStamp

] ,

[ rdf:type owl:Restriction ;

owl:onProperty <http://www.co-ode.org/ontologies/uia/ont.owl#hasNextAppointment> ;

owl:maxQualifiedCardinality "1"^^xsd:nonNegativeInteger ;

owl:onDataRange xsd:dateTimeStamp

] .

### http://www.co-ode.org/ontologies/uia/ont.owl#Vital_sign_finding

<http://www.co-ode.org/ontologies/uia/ont.owl#Vital_sign_finding> rdf:type owl:Class ;

owl:equivalentClass [ rdf:type owl:Restriction ;

owl:onProperty <http://www.co-ode.org/ontologies/uia/ont.owl#hasSCTID> ;

owl:hasValue "118227000"^^xsd:long

] ;

rdfs:subClassOf <http://www.co-ode.org/ontologies/uia/ont.owl#General_body_state_finding> ;

rdfs:comment """Vital signs finding (finding)

SCTID: 118227000

118227000 | Vital signs finding (finding) |

en Vital signs finding

en Vital signs finding (finding)""" .

### http://www.co-ode.org/ontologies/uia/ont.owl#Waist_circumference

<http://www.co-ode.org/ontologies/uia/ont.owl#Waist_circumference> rdf:type owl:Class ;

owl:equivalentClass [ rdf:type owl:Restriction ;

owl:onProperty <http://www.co-ode.org/ontologies/uia/ont.owl#hasSCTID> ;

owl:hasValue "276361009"^^xsd:long

] ;

rdfs:subClassOf <http://www.co-ode.org/ontologies/uia/ont.owl#Circumference_measure> ;

rdfs:comment """Waist circumference (observable entity)

SCTID: 276361009

276361009 | Waist circumference (observable entity) |

en Waist circumference (observable entity)

en Waist circumference""" .

### http://www.co-ode.org/ontologies/uia/ont.owl#Waist_hip_ratio

<http://www.co-ode.org/ontologies/uia/ont.owl#Waist_hip_ratio> rdf:type owl:Class ;

owl:equivalentClass [ rdf:type owl:Restriction ;

owl:onProperty <http://www.co-ode.org/ontologies/uia/ont.owl#hasSCTID> ;

owl:hasValue "248367009"^^xsd:long

] ;

rdfs:subClassOf <http://www.co-ode.org/ontologies/uia/ont.owl#Body_measure> ;

rdfs:comment """Waist/hip ratio (observable entity)

SCTID: 248367009

248367009 | Waist/hip ratio (observable entity) |

en Waist/hip ratio (observable entity)

en Waist/hip ratio""" .

### http://www.co-ode.org/ontologies/uia/ont.owl#Weight_finding

<http://www.co-ode.org/ontologies/uia/ont.owl#Weight_finding> rdf:type owl:Class ;

owl:equivalentClass [ rdf:type owl:Restriction ;

owl:onProperty <http://www.co-ode.org/ontologies/uia/ont.owl#hasSCTID> ;

owl:hasValue "107647005"^^xsd:long

] ;

rdfs:subClassOf <http://www.co-ode.org/ontologies/uia/ont.owl#Body_measurement_finding> ;

rdfs:comment """Weight finding (finding)

SCTID: 107647005

107647005 | Weight finding (finding) |

en Weight finding

en Weight finding (finding)""" .

### http://www.co-ode.org/ontologies/uia/ont.owl#Weight_for_height

<http://www.co-ode.org/ontologies/uia/ont.owl#Weight_for_height> rdf:type owl:Class ;

owl:equivalentClass [ rdf:type owl:Restriction ;

owl:onProperty <http://www.co-ode.org/ontologies/uia/ont.owl#hasSCTID> ;

owl:hasValue "248358009"^^xsd:long

] ;

rdfs:subClassOf <http://www.co-ode.org/ontologies/uia/ont.owl#Body_mass_index> ;

rdfs:comment """Weight for height (observable entity)

SCTID: 248358009

248358009 | Weight for height (observable entity) |

en Weight for height

en Weight for height (observable entity)

en Ponderal index""" .

### http://www.co-ode.org/ontologies/uia/ont.owl#Body_mass_index_30+_obesity_finding

<http://www.co-ode.org/ontologies/uia/ont.owl#Body_mass_index_30+_obesity_finding> rdf:type owl:Class ;

owl:equivalentClass [ rdf:type owl:Restriction ;

owl:onProperty <http://www.co-ode.org/ontologies/uia/ont.owl#hasSCTID> ;

owl:hasValue "162864005"^^xsd:long

] ;

rdfs:subClassOf <http://www.co-ode.org/ontologies/uia/ont.owl#Obese_finding> ;

rdfs:comment """Body mass index 30+ - obesity (finding)

SCTID: 162864005

162864005 | Body mass index 30+ - obesity (finding) |

en Body mass index 30+ - obesity

en Body mass index 30+ - obesity (finding)

en BMI 30+ - obesity""" .

### http://www.co-ode.org/ontologies/uia/ont.owl#Body_mass_index_40+_severely_obese_finding

<http://www.co-ode.org/ontologies/uia/ont.owl#Body_mass_index_40+_severely_obese_finding> rdf:type owl:Class ;

owl:equivalentClass [ rdf:type owl:Restriction ;

owl:onProperty <http://www.co-ode.org/ontologies/uia/ont.owl#hasSCTID> ;

owl:hasValue "408512008"^^xsd:long

] ;

rdfs:subClassOf <http://www.co-ode.org/ontologies/uia/ont.owl#Obese_finding> ;

rdfs:comment """Body mass index 40+ - severely obese (finding)

SCTID: 408512008

408512008 | Body mass index 40+ - severely obese (finding) |

en Body mass index 40+ - morbidly obese

en Body mass index 40+ - severely obese

en Body mass index 40+ - severely obese (finding)""" .

### http://www.co-ode.org/ontologies/uia/ont.owl#Pulse,function

<http://www.co-ode.org/ontologies/uia/ont.owl#Pulse,function> rdf:type owl:Class ;

owl:equivalentClass [ rdf:type owl:Restriction ;

owl:onProperty <http://www.co-ode.org/ontologies/uia/ont.owl#hasSCTID> ;

owl:hasValue "8499008"^^xsd:long

] ;

rdfs:subClassOf <http://www.co-ode.org/ontologies/uia/ont.owl#Cardiovascular_function> ,

[ rdf:type owl:Restriction ;

owl:onProperty ssn:isFoundBy ;

owl:qualifiedCardinality "1"^^xsd:nonNegativeInteger ;

owl:onClass <http://www.co-ode.org/ontologies/uia/ont.owl#Pulse_rate_finding>

] ;

rdfs:comment """Pulse, function (observable entity)

SCTID: 8499008

8499008 | Pulse, function (observable entity) |

en Pulse

en Pulse, function (observable entity)

en Pulse, function""" .

### http://www.co-ode.org/ontologies/uia/ont.owl#Respiratory_rate_AND/OR_rhythm_finding

<http://www.co-ode.org/ontologies/uia/ont.owl#Respiratory_rate_AND/OR_rhythm_finding> rdf:type owl:Class ;

owl:equivalentClass [ rdf:type owl:Restriction ;

owl:onProperty <http://www.co-ode.org/ontologies/uia/ont.owl#hasSCTID> ;

owl:hasValue "106049001"^^xsd:long

] ;

rdfs:subClassOf <http://www.co-ode.org/ontologies/uia/ont.owl#Vital_sign_finding> ;

rdfs:comment """Respiratory rate AND/OR rhythm finding (finding)

SCTID: 106049001

106049001 | Respiratory rate AND/OR rhythm finding (finding) |

en Respiratory rate AND/OR rhythm finding

en Respiratory rate AND/OR rhythm finding (finding)""" .

### http://www.loa-cnr.it/ontologies/DUL.owl#Event

DUL:Event rdf:type owl:Class ;

rdfs:subClassOf <http://www.co-ode.org/ontologies/uia/ont.owl#PhysicalDeviceThing> .

### http://www.loa-cnr.it/ontologies/DUL.owl#InformationObject

DUL:InformationObject rdf:type owl:Class ;

rdfs:subClassOf <http://www.co-ode.org/ontologies/uia/ont.owl#PhysicalDeviceThing> .

### http://www.loa-cnr.it/ontologies/DUL.owl#Method

DUL:Method rdf:type owl:Class .

### http://www.loa-cnr.it/ontologies/DUL.owl#ObservableEntity

DUL:ObservableEntity rdf:type owl:Class ;

rdfs:subClassOf <http://www.co-ode.org/ontologies/uia/ont.owl#PhysicalDeviceThing> .

### http://www.loa-cnr.it/ontologies/DUL.owl#PhysicalObject

DUL:PhysicalObject rdf:type owl:Class ;

rdfs:subClassOf <http://www.co-ode.org/ontologies/uia/ont.owl#PhysicalDeviceThing> .

### http://www.loa-cnr.it/ontologies/DUL.owl#Process

DUL:Process rdf:type owl:Class ;

rdfs:subClassOf <http://www.co-ode.org/ontologies/uia/ont.owl#PhysicalDeviceThing> .

### http://www.loa-cnr.it/ontologies/DUL.owl#Quality

DUL:Quality rdf:type owl:Class ;

rdfs:subClassOf <http://www.co-ode.org/ontologies/uia/ont.owl#PhysicalDeviceThing> .

### http://www.loa-cnr.it/ontologies/DUL.owl#Situation

DUL:Situation rdf:type owl:Class ;

rdfs:subClassOf <http://www.co-ode.org/ontologies/uia/ont.owl#PhysicalDeviceThing> .

#################################################################

# Individuals

#################################################################

### http://purl.oclc.org/NET/ssnx/ssn#ActivityDataValue_1

ssn:ActivityDataValue_1 rdf:type owl:NamedIndividual ,

<http://www.co-ode.org/ontologies/uia/ont.owl#ActivityDataValue> ;

<http://www.co-ode.org/ontologies/uia/ont.owl#hasTimeStamp> ssn:TemporalEntiity_1 ;

<http://www.co-ode.org/ontologies/uia/ont.owl#hasActivityBouts> 300 ;

<http://www.co-ode.org/ontologies/uia/ont.owl#hasCurrentHeartRate> 70 ;

<http://www.co-ode.org/ontologies/uia/ont.owl#hasDistanceCovered> 9500 ;

<http://www.co-ode.org/ontologies/uia/ont.owl#hasPhysicalActivityType> "Active" ;

<http://www.co-ode.org/ontologies/uia/ont.owl#hasSedentaryBouts> 120 ;

<http://www.co-ode.org/ontologies/uia/ont.owl#hasSteps> 13000 ;

<http://www.co-ode.org/ontologies/uia/ont.owl#hasTotalSleepTime> 480 .

### http://purl.oclc.org/NET/ssnx/ssn#ActivityData_1

ssn:ActivityData_1 rdf:type owl:NamedIndividual ,

<http://www.co-ode.org/ontologies/uia/ont.owl#ActivityData> ;

<http://www.co-ode.org/ontologies/uia/ont.owl#hasBeenCollectedBy> ssn:ActivityDataValue_1 ,

ssn:DailyActivityData_1 .

### http://purl.oclc.org/NET/ssnx/ssn#ActivityRecommendation_1

ssn:ActivityRecommendation_1 rdf:type owl:NamedIndividual ,

<http://www.co-ode.org/ontologies/uia/ont.owl#ActivityRecommendation> .

### http://purl.oclc.org/NET/ssnx/ssn#ContextualData_1

ssn:ContextualData_1 rdf:type owl:NamedIndividual ,

<http://www.co-ode.org/ontologies/uia/ont.owl#ContextualData> ;

<http://www.co-ode.org/ontologies/uia/ont.owl#hasBeenCollectedBy> ssn:ExternalWeatherValue_1 ;

<http://www.co-ode.org/ontologies/uia/ont.owl#hasTimeStamp> ssn:TemporalEntiity_1 .

### http://purl.oclc.org/NET/ssnx/ssn#DailyActivityData_1

ssn:DailyActivityData_1 rdf:type owl:NamedIndividual ,

ssn:DailyActivityData ;

<http://www.co-ode.org/ontologies/uia/ont.owl#hasTimeStamp> ssn:TemporalEntiity_1 ;

<http://www.co-ode.org/ontologies/uia/ont.owl#hasDailyWeight> "63.2"^^xsd:double ;

<http://www.co-ode.org/ontologies/uia/ont.owl#hasDurationOfIntensiveActivity> 20 ;

<http://www.co-ode.org/ontologies/uia/ont.owl#hasNonWearDeviceTime> 600 .

### http://purl.oclc.org/NET/ssnx/ssn#DailyHabitData_1

ssn:DailyHabitData_1 rdf:type owl:NamedIndividual ,

ssn:DailyHabitData ;

<http://www.co-ode.org/ontologies/uia/ont.owl#hasTimeStamp> ssn:TemporalEntiity_1 ;

ssn:hasAlcoholQuantity 0 ;

ssn:hasConsumedAlcohol "no" ;

ssn:hasSmoked "no" ;

ssn:hasSnusQuantity 8 ;

ssn:hasTakenSnus "yes" ;

ssn:hasTobaccoCount 0 .

### http://purl.oclc.org/NET/ssnx/ssn#DailyNutritionData_1

ssn:DailyNutritionData_1 rdf:type owl:NamedIndividual ,

ssn:DailyNutritionData ;

<http://www.co-ode.org/ontologies/uia/ont.owl#hasTimeStamp> ssn:TemporalEntiity_1 ;

ssn:hasFriedorProcessedFood "yes" ;

ssn:hasFriedorProcessedFoodFrequency 3 ;

ssn:hasFruitAmount 100 ;

ssn:hasSweetBakeries "yes" ;

ssn:hasSweetBakeriesFrequency 1 ;

ssn:hasSweetBeverages "yes" ;

ssn:hasSweetBeveragesAmount 100 ;

ssn:hasSweetFoodorMilkProduct "no" ;

ssn:hasSweetFoodorMilkProductFrequency 0 ;

ssn:hasVegetableAmount 400 ;

<http://www.co-ode.org/ontologies/uia/ont.owl#hasFruits> "yes" ;

<http://www.co-ode.org/ontologies/uia/ont.owl#hasVegetables> "yes" .

### http://purl.oclc.org/NET/ssnx/ssn#DailyPhysiologicalData_1

ssn:DailyPhysiologicalData_1 rdf:type owl:NamedIndividual ,

ssn:DailyPhysiologicalData ;

<http://www.co-ode.org/ontologies/uia/ont.owl#hasTimeStamp> ssn:TemporalEntiity_1 ;

<http://www.co-ode.org/ontologies/uia/ont.owl#hasCurrentBMIValue> "22.4"^^xsd:double .

### http://purl.oclc.org/NET/ssnx/ssn#DietaryRecommendation_1

ssn:DietaryRecommendation_1 rdf:type owl:NamedIndividual ,

<http://www.co-ode.org/ontologies/uia/ont.owl#DietaryRecommendation> .

### http://purl.oclc.org/NET/ssnx/ssn#ExternalWeatherValue_1

ssn:ExternalWeatherValue_1 rdf:type owl:NamedIndividual ,

<http://www.co-ode.org/ontologies/uia/ont.owl#ExternalWeatherValue> ;

<http://www.co-ode.org/ontologies/uia/ont.owl#hasFoggyForeCast> "no" ;

<http://www.co-ode.org/ontologies/uia/ont.owl#hasHighTemperatureForeCast> 19 ;

<http://www.co-ode.org/ontologies/uia/ont.owl#hasLowTemperatureForeCast> 12 ;

<http://www.co-ode.org/ontologies/uia/ont.owl#hasRainingForeCast> "no" ;

<http://www.co-ode.org/ontologies/uia/ont.owl#hasSnowingForeCast> "no" ;

<http://www.co-ode.org/ontologies/uia/ont.owl#hasSunnyForeCast> "yes" ;

<http://www.co-ode.org/ontologies/uia/ont.owl#hasTemperature> 14 ;

<http://www.co-ode.org/ontologies/uia/ont.owl#hasWeatherSatus> "Mostly Sunny" .

### http://purl.oclc.org/NET/ssnx/ssn#HabitData_1

ssn:HabitData_1 rdf:type owl:NamedIndividual ,

<http://www.co-ode.org/ontologies/uia/ont.owl#HabitData> ;

<http://www.co-ode.org/ontologies/uia/ont.owl#hasBeenCollectedBy> ssn:DailyHabitData_1 .

### http://purl.oclc.org/NET/ssnx/ssn#Individual_1

ssn:Individual_1 rdf:type owl:NamedIndividual ,

<http://www.co-ode.org/ontologies/uia/ont.owl#Participant> ;

<http://www.co-ode.org/ontologies/uia/ont.owl#hasHealthRecord> ssn:ParticipantHealthRecord_1 ;

<http://www.co-ode.org/ontologies/uia/ont.owl#hasReceivedRecommendation> ssn:Recommendation_1 ;

<http://www.co-ode.org/ontologies/uia/ont.owl#hasStatus> ssn:ParticipantStatus_1 ;

ssn:hasUserId "Individual_1" ;

<http://www.co-ode.org/ontologies/uia/ont.owl#hasPassword> "Password_1" ;

<http://www.co-ode.org/ontologies/uia/ont.owl#hasRole> "Participant" .

### http://purl.oclc.org/NET/ssnx/ssn#Individual_2

ssn:Individual_2 rdf:type owl:NamedIndividual ,

<http://www.co-ode.org/ontologies/uia/ont.owl#Participant> .

### http://purl.oclc.org/NET/ssnx/ssn#Individual_3

ssn:Individual_3 rdf:type owl:NamedIndividual ,

<http://www.co-ode.org/ontologies/uia/ont.owl#Participant> .

### http://purl.oclc.org/NET/ssnx/ssn#Individual_4

ssn:Individual_4 rdf:type owl:NamedIndividual ,

<http://www.co-ode.org/ontologies/uia/ont.owl#Participant> .

### http://purl.oclc.org/NET/ssnx/ssn#NutritionData_1

ssn:NutritionData_1 rdf:type owl:NamedIndividual ,

<http://www.co-ode.org/ontologies/uia/ont.owl#NutritionData> ;

<http://www.co-ode.org/ontologies/uia/ont.owl#hasBeenCollectedBy> ssn:DailyNutritionData_1 .

### http://purl.oclc.org/NET/ssnx/ssn#ObservableEntity_1

ssn:ObservableEntity_1 rdf:type owl:NamedIndividual ,

DUL:ObservableEntity .

### http://purl.oclc.org/NET/ssnx/ssn#ParticipantHealthRecord_1

ssn:ParticipantHealthRecord_1 rdf:type owl:NamedIndividual ,

<http://www.co-ode.org/ontologies/uia/ont.owl#ParticipantHealthRecord> ;

ssn:hasObservables ssn:ObservableEntity_1 .

### http://purl.oclc.org/NET/ssnx/ssn#ParticipantState_1

ssn:ParticipantState_1 rdf:type owl:NamedIndividual ,

<http://www.co-ode.org/ontologies/uia/ont.owl#ParticipantState> .

### http://purl.oclc.org/NET/ssnx/ssn#ParticipantStatus_1

ssn:ParticipantStatus_1 rdf:type owl:NamedIndividual ,

<http://www.co-ode.org/ontologies/uia/ont.owl#ParticipantStatus> ;

<http://www.co-ode.org/ontologies/uia/ont.owl#hasParticipantState> ssn:ParticipantState_1 ;

<http://www.co-ode.org/ontologies/uia/ont.owl#hasParticipationStatus> "Active" .

### http://purl.oclc.org/NET/ssnx/ssn#PhysiologicalData_1

ssn:PhysiologicalData_1 rdf:type owl:NamedIndividual ,

<http://www.co-ode.org/ontologies/uia/ont.owl#PhysiologicalData> ;

<http://www.co-ode.org/ontologies/uia/ont.owl#hasBeenCollectedBy> ssn:ActivityDataValue_1 ,

ssn:DailyPhysiologicalData_1 .

### http://purl.oclc.org/NET/ssnx/ssn#Questionnaire_1

ssn:Questionnaire_1 rdf:type owl:NamedIndividual ,

<http://www.co-ode.org/ontologies/uia/ont.owl#Questionnaire> ;

<http://www.co-ode.org/ontologies/uia/ont.owl#hasTimeStamp> ssn:TemporalEntiity_1 .

### http://purl.oclc.org/NET/ssnx/ssn#RecommendationMessages_1

ssn:RecommendationMessages_1 rdf:type owl:NamedIndividual ,

<http://www.co-ode.org/ontologies/uia/ont.owl#RecommendationMessages> ;

ssn:hasActivityMessages "A-4: Good work. Please keep it up. You are active." ;

ssn:hasContextualMessages "C-1: Tomorrow the weather is favorable for outdoor activities." ;

ssn:hasDietaryMessages "D-2: Avoid consuming discretionary food items. Try to eat core-healthy foods." ,

"D-3: Avoid sugary soft drinks, energy drinks, and fruit juices. Drink plenty of water." ;

ssn:hasHabitRelatedMessages "H-1: Avoid tobacco consumption. Please, try to reduce it." .

### http://purl.oclc.org/NET/ssnx/ssn#Recommendation_1

ssn:Recommendation_1 rdf:type owl:NamedIndividual ,

<http://www.co-ode.org/ontologies/uia/ont.owl#Recommendation> ;

ssn:hasContextData ssn:ContextualData_1 ;

<http://www.co-ode.org/ontologies/uia/ont.owl#hasMessages> ssn:RecommendationMessages_1 ;

<http://www.co-ode.org/ontologies/uia/ont.owl#hasParticipantStatus> ssn:ParticipantStatus_1 ;

<http://www.co-ode.org/ontologies/uia/ont.owl#hasTimeStamp> ssn:TemporalEntiity_1 ;

<http://www.co-ode.org/ontologies/uia/ont.owl#hasUsedHealthRecord> ssn:ParticipantHealthRecord_1 .

### http://purl.oclc.org/NET/ssnx/ssn#TemporalEntiity_1

ssn:TemporalEntiity_1 rdf:type owl:NamedIndividual ,

<http://www.co-ode.org/ontologies/uia/ont.owl#TemporalEntity> ;

<http://www.co-ode.org/ontologies/uia/ont.owl#hasDateTime> "2020-08-03T13:20:00-05:00"^^xsd:dateTimeStamp .

### http://www.co-ode.org/ontologies/uia/ont.owl#ADMIN

<http://www.co-ode.org/ontologies/uia/ont.owl#ADMIN> rdf:type owl:NamedIndividual ,

<http://www.co-ode.org/ontologies/uia/ont.owl#Admin> .

### http://www.co-ode.org/ontologies/uia/ont.owl#Ayan

<http://www.co-ode.org/ontologies/uia/ont.owl#Ayan> rdf:type owl:NamedIndividual ,

<http://www.co-ode.org/ontologies/uia/ont.owl#Researcher> .

### http://www.co-ode.org/ontologies/uia/ont.owl#Kent

<http://www.co-ode.org/ontologies/uia/ont.owl#Kent> rdf:type owl:NamedIndividual ,

<http://www.co-ode.org/ontologies/uia/ont.owl#Nurse> .
